# Supplementary material for: Jujube Fruit Metabolomic Profiles Reveal Cultivar Differences and Function as Cultivar Fingerprints
Source: Plants (Basel). 2023 Jun 14;12(12):2313. doi: 10.3390/plants12122313 (PMC10301335; doi:10.3390/plants12122313)
Supplement: Supplementary file 1 [file plants-12-02313-s001.zip › plants-2452724-supplementary.pdf]

## Supplementary Information

# Jujube fruit metabolomic profiles reveal cultivar differences and function as cultivar fingerprints

Shengrui Yao [1,3]*, Dikshya Sapkota[1], Jordan Hungerford[2], and Roland Kersten[2]

1. Department of Plant and Environmental Sciences, New Mexico State University, Las Cruces, NM 88003, USA;
2. Department of Medicinal Chemistry, College of Pharmacy, University of Michigan, Ann Arbor, MI, 48109, USA;
3. Sustainable Agriculture Sciences Center, New Mexico State University, Alcalde, NM 87511, USA.

| Figure/Table | Description | Page |
|---|---|---|
| Table S1 | Significant differential metabolites and corresponding metabolic pathways between cultivars | 2 |
| Figure S1 | Heatmap of all cultivars, locations and metabolites with clustering for both locations and metabolites | 3 |
| Figure S2 | PCA eclipse figures of 11 jujube cultivars at three locations - AL: Alcalde, NM; LK - Leyendecker/Las Cruces, NM, and LL-Los Lunas, NM. | 5 |
| Figure S3 | All cultivars and all metabolites heatmap | 6 |
| Figure S4 | Heatmaps of differential metabolites of Li vs Shanxi Li and JS vs JKW. | 8 |
| Figure S5 | Pairwise differential metabolite analysis between *Ziziphus jujuba* cultivars | 9 |
| Figure S6 | MS analysis of a sanjoinine A | 64 |
| Figure S7 | Significant metabolites of 11 jujube cultivars in different categories | 65 |

**Table S1 | Significant differential metabolites and corresponding metabolic pathways between cultivars.** A vs B, if down-regulated, A has more metabolite than B; if up-regulated, B has more metabolite than A. Abbreviations: MP – multipurpose.

| Cultivar groups (fruit types) | All significant differential metabolites | Up-regulated metabolites | Down-regulated metabolites | Up-regulated metabolic pathways | Down-regulated metabolic pathways | Differential secondary metabolites [%] |
|---|---|---|---|---|---|---|
| Alcalde1/ Dongzao | 475 | 246 | 229 | Stilbenoid, Flavone & Flavonol, Glutathione, Sulfur | Arginine, Linoleic acid, Alanine/Aspartate/Glutamate, Proline, Porphyrin, Nitrogen, Cyanoamino acid, Valine/Leucine/Isoleucine, D-amino acid, Pyrimidine, Glyoxylate and decarboxylate, Butanoate, Propanoate | 41.4 |
| Alcalde1/Jinsi | 375 | 192 | 183 | Arginine & Proline, Purine alkaloids, Phenylpropanoid, Pyrimidine, Nucleotides, Tropane/piperidine/pyridine alkaloids, Betalain, Fatty acid, alpha-linoleic acid, glutathione, Purine, Stilbenoid | Sphingolipid, Flavonoid | 40.5 |
| Alcalde1/Jixin | 415 | 173 | 242 | Flavonoid, Phenylpropanoid, Tryptophan, Arginine & Proline | Nucleotide, Pyrimidine, Purine, Arginine, D-amino acid, Linoleic acid | 41.1 |
| Alcalde1/JKW | 422 | 171 | 251 | Phenylpropanoid, Arginine & Proline, Flavonoid, Flavone & Flavonol | Pyrimidine, Nucleotide | 40.5 |
| Alcalde1/KFC | 483 | 365 | 118 | Flavonoid, Flavone & flavonol, Phenylpropanoid, Stilbenoid | Tryptophan | 53.7 |
| Alcalde1/Lang | 330 | 212 | 118 | Flavonoid, Phenylpropanoid, Tryptophan, Phenylalanine, Glutathione | | 38.5 |
| Alcalde1/Li | 336 | 206 | 130 | Flavonoid, Sphingolipid, Quinone | Tyrosine, Isoquinoline alkaloids, Nucleotide | 42.6 |
| Alcalde1/Maya | 489 | 368 | 121 | Phenylalanine, Phenylpropanoid, Flavonoid, Arginine & Proline | Flavone & Flavonol | 42.5 |
| Alcalde1/ Shanxi Li | 378 | 103 | 275 | Flavonoid, Quinone, Flavone & Flavonol, Purine, Nucleotide | Tyrosine, Isoquinoline alkaloids | 55.6 |
| Alcalde1/ZCW | 404 | 203 | 201 | Arginine & Proline, Phenylpropanoid | Nucleotide | 43.7 |
| Dongzao/Jinsi | 544 | 278 | 266 | Linoleic acid, Cyanoamino acid, Amino acid, Tropane/piperidine/pyridine alkaloid, Quinone, 2-oxocarboxylic acid, glucosinolate | Sphingolipid | 45.8 |
| Dongzao/Jixin | 511 | 230 | 281 | Arginine & Proline, Nucleotide, Purine | Glutathione, Flavone & Flavonol | 42.6 |
| Dongzao/JKW | 564 | 316 | 248 | Linoleic acid, Nucleotide, Purine, Arginine & Proline, Phenylalanine, D-amino acid, | Flavone & Flavonol | 39.4 |
| Dongzao/KFC | 531 | 403 | 128 | Arginine & Proline, Flavonoid, Isoquinoline alkaloid, Linoleic acid, Flavone & flavonol, Tyrosine, Phenylalanine | Glutathione | 42.4 |
| Dongzao/Lang | 520 | 209 | 311 | Amino acid, Cyanoamino acid, D-amino acid, Glutathione, Nucleotide | Phenylpropanoid | 48.2 |
| Dongzao/Li | 487 | 260 | 227 | Amino acid, Flavonoid, Linoleic acid, Sphingolipid | Phenylpropanoid, Flavone & flavonol, Sulfur | 50 |
| Dongzao/Maya | 575 | 400 | 175 | Linoleic acid, Quinone, Tyrosine, Arginine & proline, Tropane/piperidine/pyridine alkaloid, Isoquinoline alkaloid, Cyanoamino acid, Purine, Pyrimidine, Alanine/Aspartate/Glutamate, Nucleotide | | 42.4 |
| Dongzao/ Shanxi Li | 528 | 188 | 340 | Amino acids, Linoleic acid, Flavonoid, Isoquinoline alkaloid, Phenylalanine, Nucleotide | Phenylpropanoid | 46.7 |
| Dongzao/ZCW | 571 | 302 | 269 | Linoleic acid, Purine, Nucelotide, Pyrimidine, Arginine & proline | | 35.3 |
| Jinsi/Jixin | 473 | 195 | 278 | Vitamin B6 | Linoleic acid, Amino acids, Betalain, Phenylpropanoid, Glutathione | 50.7 |
| Jinsi/JKW | 47 | 36 | 11 | | Amino acids, Tropane/piperidine/pyridine alkaloid, glucosinolate, 2-oxocarboxylic acid | 62.5 |
| Jinsi/KFC | 494 | 379 | 115 | Sphingolipid, Flavonoid, Flavone & Flavonol | Cyanoamino acid, Thiamine, Amino acids, Nucleotide, Pantothenate & CoA, Purine | 46.8 |
| Jinsi/Lang | 420 | 270 | 150 | Fructose & Mannose, Nucleotide, | Linoleic acid, Phenylpropanoid, D-amino acid | 43.5 |
| Jinsi/Li | 403 | 195 | 208 | Sphingolipid, Flavonoid, Flavone & Flavonol, Lysine | Linoleic acid, Tropane/piperidine/pyridine alkaloid, zeatin, phenylpropanoid, arginine & proline, glutathione, Nucleotide | 39.8 |
| Jinsi/Maya | 533 | 394 | 139 | Phenylalanine, Tyrosine, Arginine & proline | Purine, Nucleotide, Glutathione | 39.2 |
| Jinsi/Shanxi Li | 473 | 178 | 295 | Flavonoid, Flavone & flavonol | Linoleic acid, Tropane/piperidine/pyridine alkaloid, phenylpropanoid | 40.2 |
| Jinsi/ZCW | 339 | 158 | 181 | | Tropane/Piperidine/Pyridine alkaloid, Glucosinolate, Amino acids, 2-oxocarboxylic acid | 45.5 |
| Jixin/JKW | 498 | 267 | 231 | Linoleic acid, Phenylpropanoid | Flavonoid | 43.9 |
| Jixin/KFC | 531 | 377 | 154 | Flavone & flavonol, Flavonoid, Phenylpropanoid, Tropane/piperidine/pyridine alkaloid, phenylalanine | Tryptophan, Purine | 56.7 |
| Jixin/Lang | 466 | 215 | 251 | Nicotinate/Nicotinamide, Glutathione, Nucleotide, Pyrimidine, Linoleic acid | Phenylpropanoid | 40.9 |
| Jixin/Li | 481 | 256 | 225 | Flavonoid, Sphingolipid, Arginine, Flavone & Flavonol | Tyrosine, Phenylpropanoid | 44.8 |
| Jixin/Maya | 574 | 192 | 382 | Phenylalanine, Tryptophan, Arginine & proline, Tropane/piperidine/pyridine alkaloid, linoleic acid, | Sulfur, flavonoid | 45.2 |
| Jixin/Shanxi Li | 553 | 245 | 308 | Flavonoid, Sphingolipid, Flavone & flavonol, Linoleic acid, Glutathione | Phenylpropanoid | 39.7 |
| Jixin/ZCW | 510 | 286 | 224 | Glutathione, Pantothenate and CoA, Linoleic acid, Flavone & flavonol, Nuceotide, | Purine | 37.1 |
| JKW/KFC | 517 | 132 | 385 | Flavonoid, Flavone & flavonol, Sphingolipid, phenylpropanoid, arginine, phenylalanine | Nucleotide, Purine | 49.2 |
| JKW/Lang | 436 | 177 | 259 | Flavonoid | Purine | 40 |
| JKW/Li | 452 | 207 | 245 | Flavonoid, Flavone & flavonol | Linoleic acid | 44.3 |
| JKW/Maya | 561 | 392 | 169 | Phenylalanine, Sphingolipid, Tyrosine, Tryptophan, Arginine & proline | Purine, Phenylpropanoid | 40 |
| JKW/Shanxi Li | 510 | 232 | 278 | Flavonoid, Flavone & Flavonols, Sphingolipid | Linoleic acid, unsaturated fatty acids, tropane/piperidine/pyridine alkaloids, phenylalanine | 43.1 |
| JKW/ZCW | 384 | 160 | 224 | Sphingolipid, Flavonoid, Flavone & flavonol | Phenylalanine, Purine | 37 |
| KFC/Lang | 498 | 178 | 320 | Glutathione | Phenylpropanoid, Flavone & flavonol, Tyrosine | 45.5 |
| KFC/Li | 492 | 154 | 338 | Glutathione, D-amino acid, | Phenylpropanoid, Flavonoid, Tropane/piperidine/pyridine alkaloid, Flavone & flavonol | 50.8 |
| KFC/Maya | 474 | 222 | 252 | Linoleic acid, Tryptophan | Purine, Nucleotide, Flavonoid, Flavone & flavonol | 52.2 |
| KFC/Shanxi Li | 504 | 293 | 211 | D-amino acid, glutathione, cofactors, tryptophan, sucrose, cyanoamino acid, cysteine & methionine, purine | phenylpropanoid, linoleic acid, betalain | 51.7 |
| KFC/ZCW | 498 | 381 | 117 | arginine & proline | flavonoid, flavone & flavonol, purine, phenylpropanoid, stilbenoid, nucleotide | 49.2 |
| Lang/Li | 355 | 149 | 206 | Purine, Nucleotide | Flavonoid, Isoquinoline alkaloid, Phenylpropanoid, Tyrosine, Phenylalanine | 50 |
| Lang/Maya | 437 | 265 | 172 | Tyrosine, Phenylpropanoid, Linoleic acid, Arginine & proline | Phenylalanine, Glutathione, Purine, Flavonoid, Nucleotide | 41.8 |
| Lang/Shanxi Li | 392 | 193 | 199 | Purine, Nucleotide | Phenylalanine, Flavonoid, Phenylalanine, Phenylpropanoid, Cysteine & methionine, Pyrimidine | 50 |
| Lang/ZCW | 445 | 181 | 264 | Arginine & proline, Tyrosine, Glutathione, Tropane/piperidine/pyridine alkaloid, phenylpropanoid | Nucleotide, Purine, D-amino acid, Flavonoid | 42.2 |
| Li/Maya | 486 | 337 | 149 | Linoleic acid, Tryptophan, Phenylpropanoid | Purine, Glutathione, Nucleotide | 44.6 |
| Li/Shanxi Li | 42 | 36 | 6 | | Glucosinolate, Amino acids, Phenylpropanoid, 2-oxocarboxylic acid | 75 |
| Li/ZCW | 436 | 198 | 238 | | Glucosinolate, Amino acids, 2-Oxocarboxylic acid | 42 |
| Maya/Shanxi Li | 500 | 297 | 203 | Purine, Nucleotide, Glutathione, Sulfur | Tryptophan, Linoleic acid, Quinones, Tyrosine, Phenylalanine, Isoquinoline alkaloid | 42.9 |
| Maya/ZCW | 519 | 130 | 389 | Tropane/piperidine/pyridine alkaloids | Phenylalanine, Tryptophan, Arginine & proline, Flavonoid | 53.1 |
| Shanxi Li/ZCW | 494 | 312 | 182 | Phenylpropanoid, Arginine & Proline, Linoleic acid | Flavonoid, Purine, D-amino acid, Pyrimidine, Nucleotide | 47.8 |

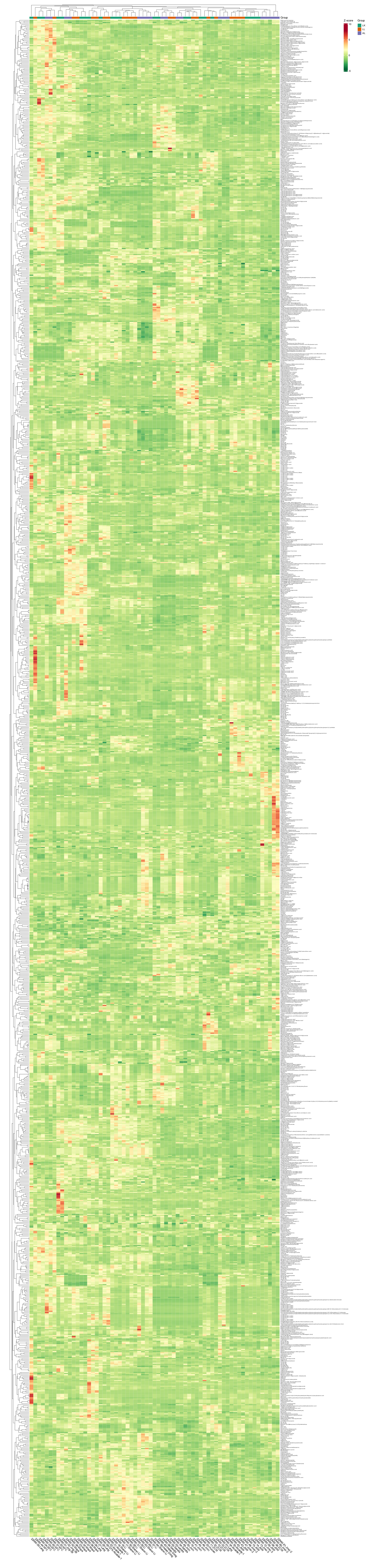

Group
Z-score
10
5
0
-5
LK
LL
AL

**Figure S1.** Heatmap of all cultivars, locations and metabolites with clustering for both locations (top) and metabolites (left).

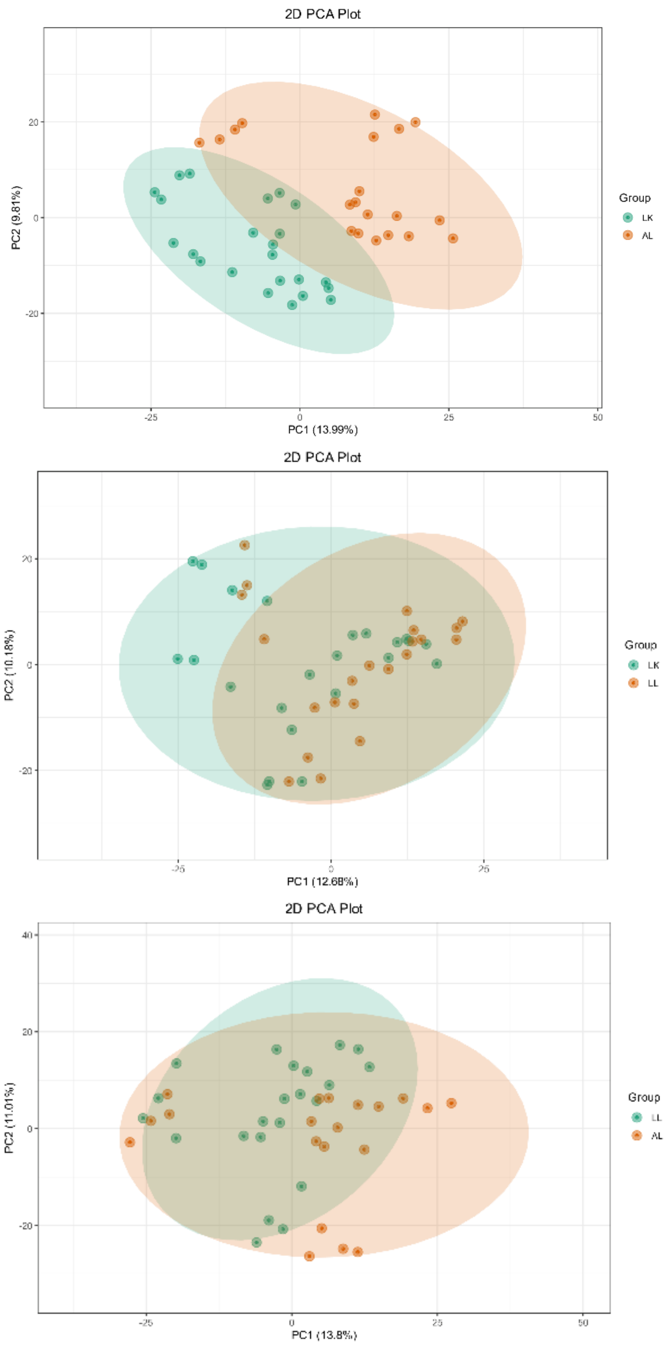


Figure S2. PCA eclipse figures of 11 jujube cultivars at three locations: left: LK vs AL (a), middle: LK vs LL, and right: LL vs AL. AL: Alcalde, NM; LK: Leyendecker/Las Cruces, NM, and LL-Los Lunas, NM.

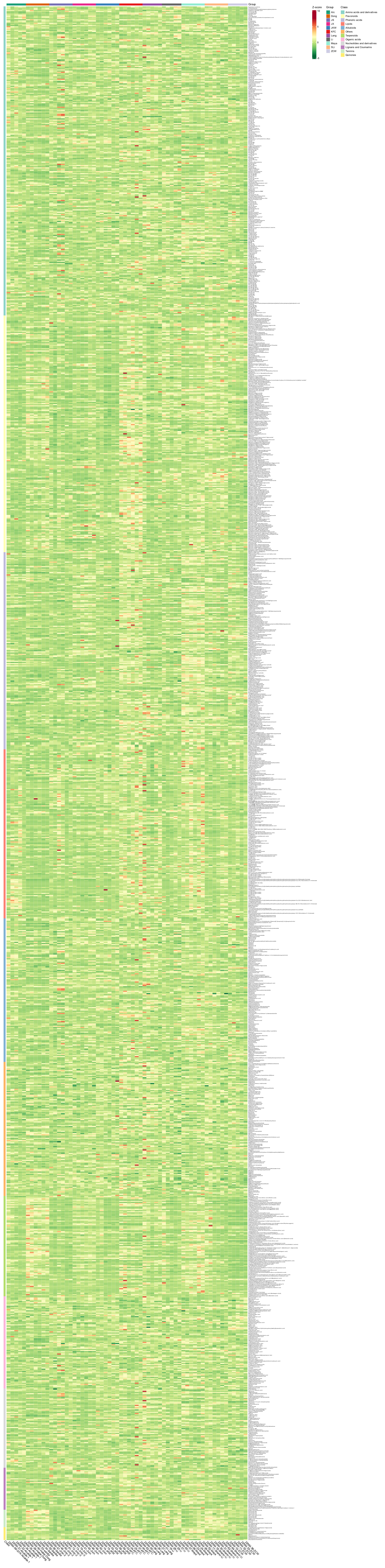

Group
Z-score
10
5
0
-5
Group
Alc
Dong
JS
JX
JKW
KFC
Lang
Li
Maya
SLi
ZCW
Class
Amino acids and derivatives
Flavonoids
Phenolic acids
Lipids
Alkaloids
Others
Terpenoids
Organic acids
Nucleotides and derivatives
Lignans and Coumarins
Tannins
Quinones

**Figure S3.** All cultivars and all metabolites heatmap. The X axis indicates cultivar names and Y axis is metabolite classes.

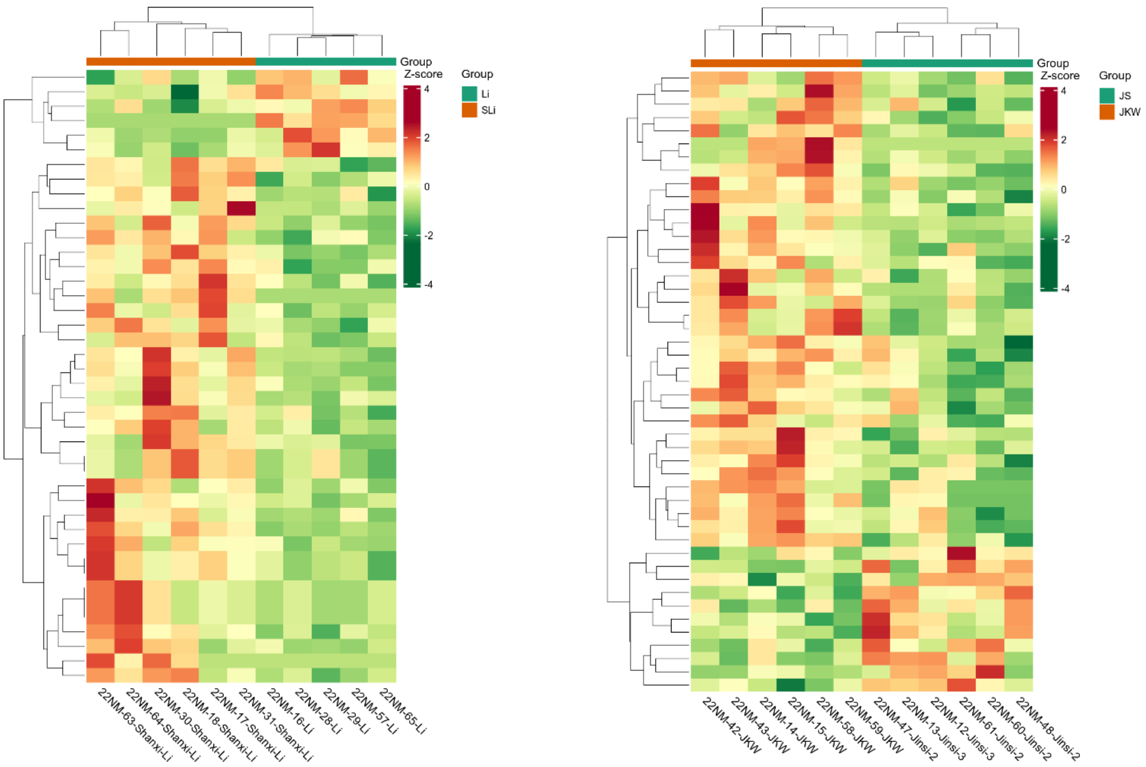


**Figure S4.** Heatmaps of differential metabolites of Li vs Shanxi Li and JS vs JKW.

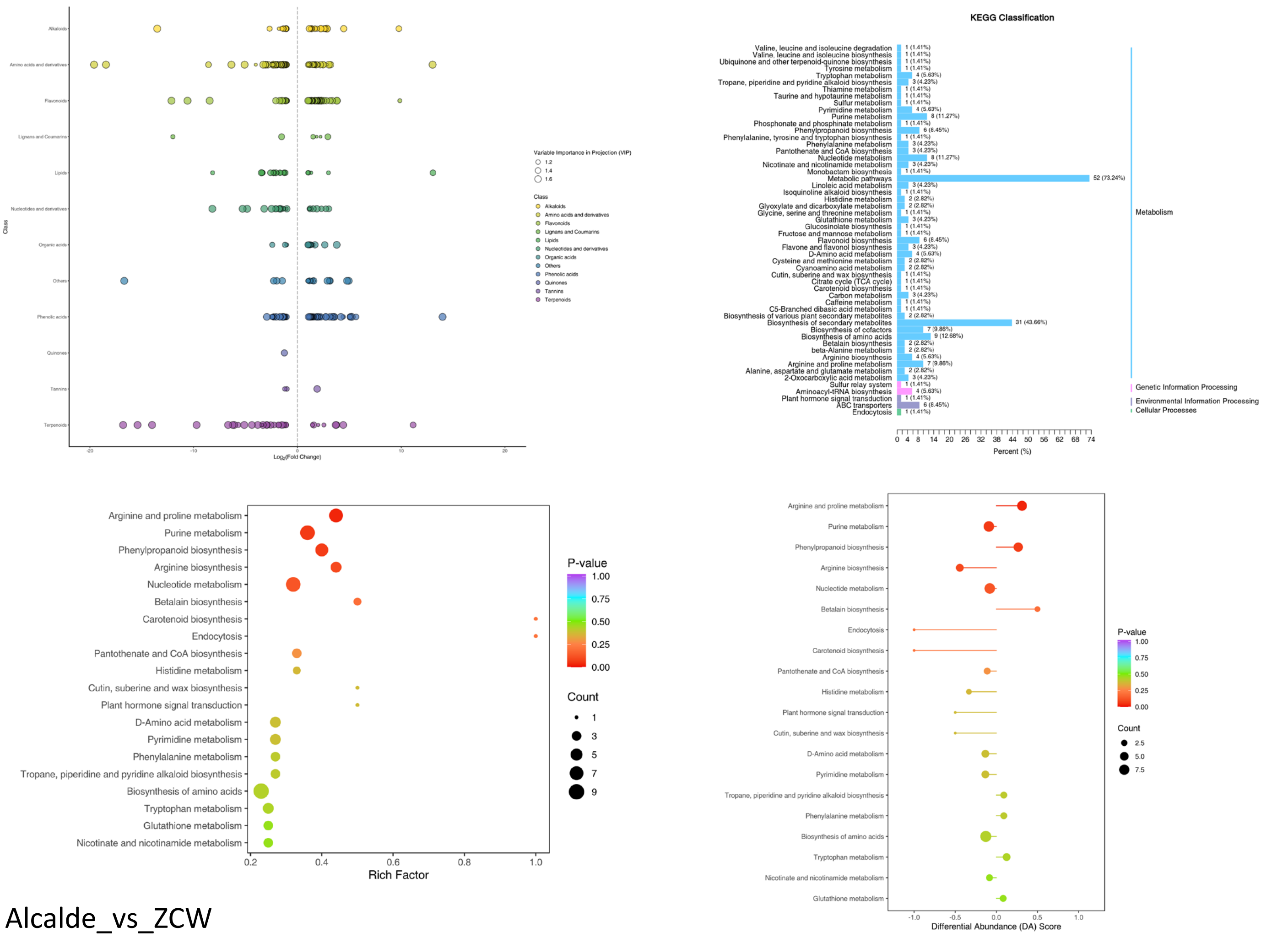


**Figure S5 | Pairwise differential metabolite analysis between *Ziziphus jujuba* cultivars. Scatter plot of differential metabolites shown on top, left**. Each dot in the graph indicates a metabolite, and different colors indicate different metabolite subclasses; the horizontal coordinate indicates the logarithmic value of the multiplicative difference in the content of a substance in two groups of samples (log22FC), the larger the absolute value of the horizontal coordinate, the greater the difference in the content of the substance between the two groups of samples, and the size of the dot represents the VIP value. **KEGG classification of differential metabolites on the top right**. The Y-axis shows the name of the KEGG pathway. The number of significant differential metabolites and the proportion of the total significant differential metabolites are shown next to the bar plot. **KEGG enrichment diagram of differential metabolites on bottom left.** The X-axis represents the Rich Factor and the Y-axis represents the pathway. The color of points reflects the p-value. The darker the red, the more significant the enrichment. The size of the dot represents the number of enriched differential metabolites. **Analysis of overall changes in KEGG metabolic pathway on bottom right.** The Y-axis represents the name of differential pathway, and the X-axis represents DA Score. DA Score reflects the overall change of all metabolites in the metabolic pathway. A Score of 1 indicates that the expression trend of all identified metabolites in this pathway is up-regulated, and -1 indicates that the expression trend of all identified metabolites in this pathway is down-regulated. The length of the line represent the absolute value of DA-score while the size of the dot at the end of the line represent the number of differential metabolites. A dot on the left of the line represent the pathway is down-regulated; a dot on the right of the line represents the pathway is up-regulated. The color of the line and dot represent the p-value. The darker the red, the smaller the p-value and the darker the purple, the larger the p-value.

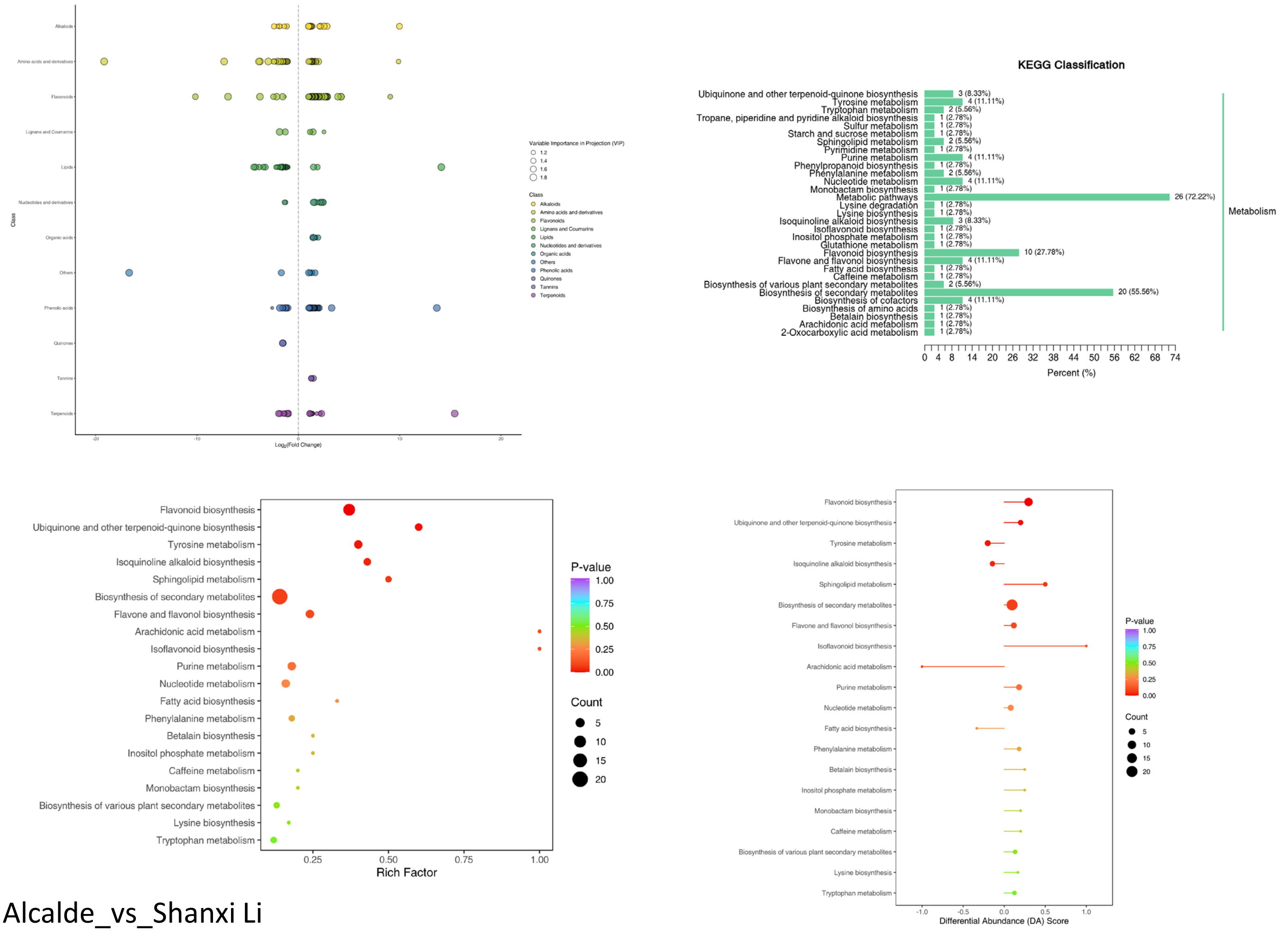


Alcalde_vs_Shanxi Li

**Figure S5 | Pairwise differential metabolite analysis between *Ziziphus jujuba* cultivars. Scatter plot of differential metabolites shown on top, left**. Each dot in the graph indicates a metabolite, and different colors indicate different metabolite subclasses; the horizontal coordinate indicates the logarithmic value of the multiplicative difference in the content of a substance in two groups of samples ($log_2 2FC$), the larger the absolute value of the horizontal coordinate, the greater the difference in the content of the substance between the two groups of samples, and the size of the dot represents the VIP value. **KEGG classification of differential metabolites on the top right**. The Y-axis shows the name of the KEGG pathway. The number of significant differential metabolites and the proportion of the total significant differential metabolites are shown next to the bar plot. **KEGG enrichment diagram of differential metabolites on bottom left.** The X-axis represents the Rich Factor and the Y-axis represents the pathway. The color of points reflects the p-value. The darker the red, the more significant the enrichment. The size of the dot represents the number of enriched differential metabolites. **Analysis of overall changes in KEGG metabolic pathway on bottom right.** The Y-axis represents the name of differential pathway, and the X-axis represents DA Score. DA Score reflects the overall change of all metabolites in the metabolic pathway. A Score of 1 indicates that the expression trend of all identified metabolites in this pathway is up-regulated, and -1 indicates that the expression trend of all identified metabolites in this pathway is down-regulated. The length of the line represent the absolute value of DA-score while the size of the dot at the end of the line represent the number of differential metabolites. A dot on the left of the line represent the pathway is down-regulated; a dot on the right of the line represents the pathway is up-regulated. The color of the line and dot represent the p-value. The darker the red, the smaller the p-value and the darker the purple, the larger the p-value.

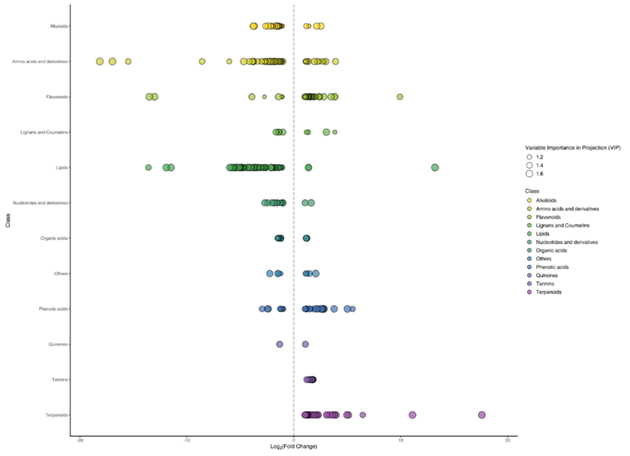

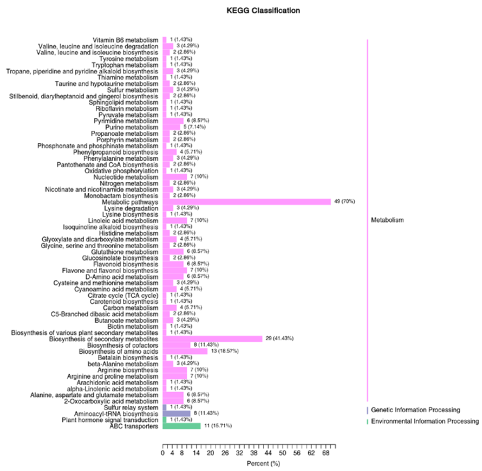

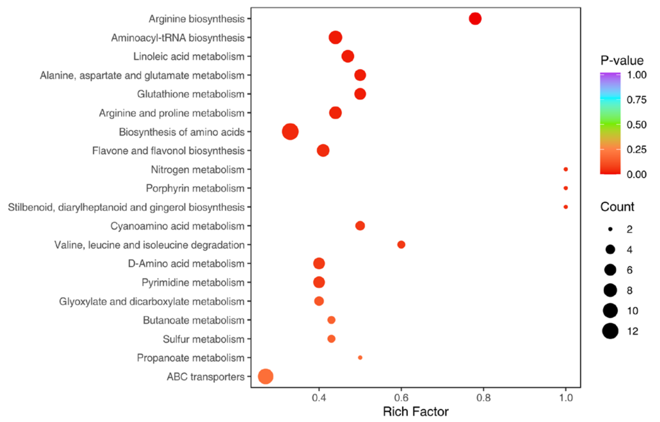


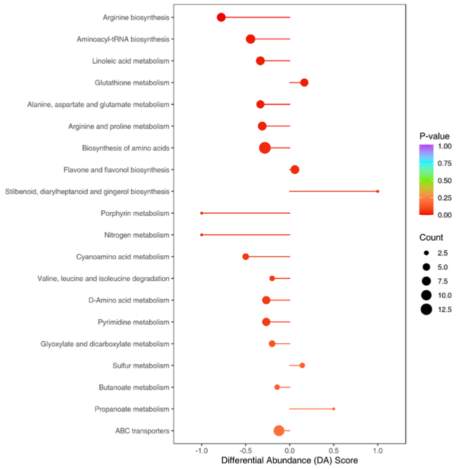


Alcalde_vs_Sandia (Hebei Dongzao)

**Figure S5 | Pairwise differential metabolite analysis between *Ziziphus jujuba* cultivars. Scatter plot of differential metabolites shown on top, left**. Each dot in the graph indicates a metabolite, and different colors indicate different metabolite subclasses; the horizontal coordinate indicates the logarithmic value of the multiplicative difference in the content of a substance in two groups of samples (log22FC), the larger the absolute value of the horizontal coordinate, the greater the difference in the content of the substance between the two groups of samples, and the size of the dot represents the VIP value. **KEGG classification of differential metabolites on the top right**. The Y-axis shows the name of the KEGG pathway. The number of significant differential metabolites and the proportion of the total significant differential metabolites are shown next to the bar plot. **KEGG enrichment diagram of differential metabolites on bottom left.** The X-axis represents the Rich Factor and the Y-axis represents the pathway. The color of points reflects the p-value. The darker the red, the more significant the enrichment. The size of the dot represents the number of enriched differential metabolites. **Analysis of overall changes in KEGG metabolic pathway on bottom right.** The Y-axis represents the name of differential pathway, and the X-axis represents DA Score. DA Score reflects the overall change of all metabolites in the metabolic pathway. A Score of 1 indicates that the expression trend of all identified metabolites in this pathway is up-regulated, and -1 indicates that the expression trend of all identified metabolites in this pathway is down-regulated. The length of the line represent the absolute value of DA-score while the size of the dot at the end of the line represent the number of differential metabolites. A dot on the left of the line represent the pathway is down-regulated; a dot on the right of the line represents the pathway is up-regulated. The color of the line and dot represent the p-value. The darker the red, the smaller the p-value and the darker the purple, the larger the p-value.

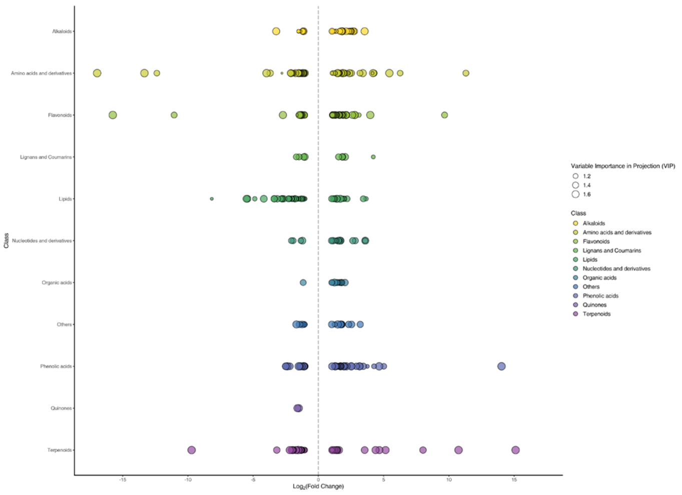


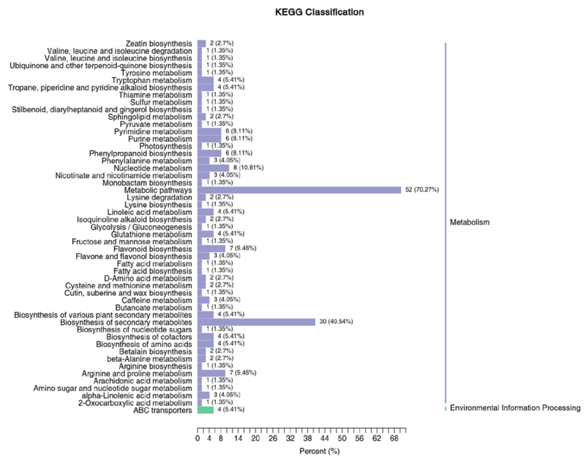


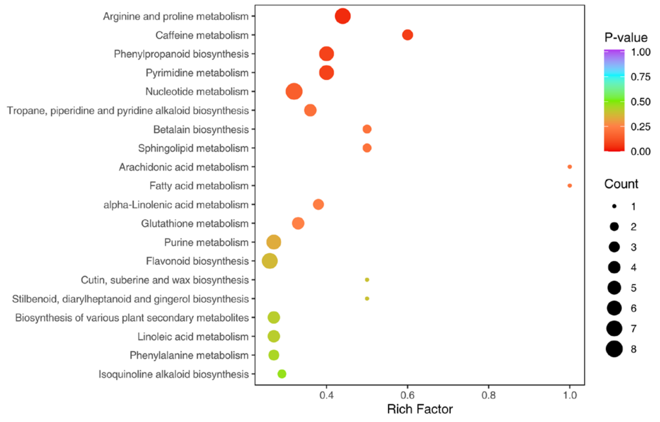


Alcalde_vs_Jinsi

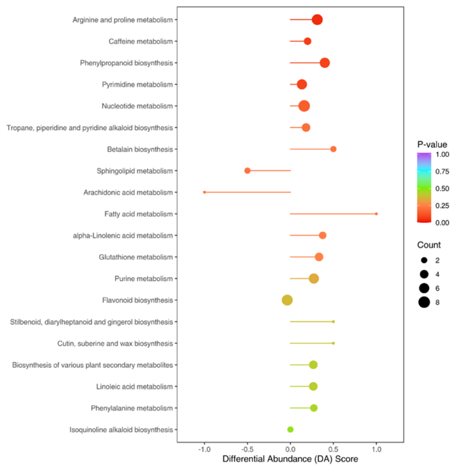


**Figure S5 | Pairwise differential metabolite analysis between *Ziziphus jujuba* cultivars. Scatter plot of differential metabolites shown on top, left.** Each dot in the graph indicates a metabolite, and different colors indicate different metabolite subclasses; the horizontal coordinate indicates the logarithmic value of the multiplicative difference in the content of a substance in two groups of samples (log22FC), the larger the absolute value of the horizontal coordinate, the greater the difference in the content of the substance between the two groups of samples, and the size of the dot represents the VIP value. **KEGG classification of differential metabolites on the top right**. The Y-axis shows the name of the KEGG pathway. The number of significant differential metabolites and the proportion of the total significant differential metabolites are shown next to the bar plot. **KEGG enrichment diagram of differential metabolites on bottom left.** The X-axis represents the Rich Factor and the Y-axis represents the pathway. The color of points reflects the p-value. The darker the red, the more significant the enrichment. The size of the dot represents the number of enriched differential metabolites. **Analysis of overall changes in KEGG metabolic pathway on bottom right.** The Y-axis represents the name of differential pathway, and the X-axis represents DA Score. DA Score reflects the overall change of all metabolites in the metabolic pathway. A Score of 1 indicates that the expression trend of all identified metabolites in this pathway is up-regulated, and -1 indicates that the expression trend of all identified metabolites in this pathway is down-regulated. The length of the line represent the absolute value of DA-score while the size of the dot at the end of the line represent the number of differential metabolites. A dot on the left of the line represent the pathway is down-regulated; a dot on the right of the line represents the pathway is up-regulated. The color of the line and dot represent the p-value. The darker the red, the smaller the p-value and the darker the purple, the larger the p-value.

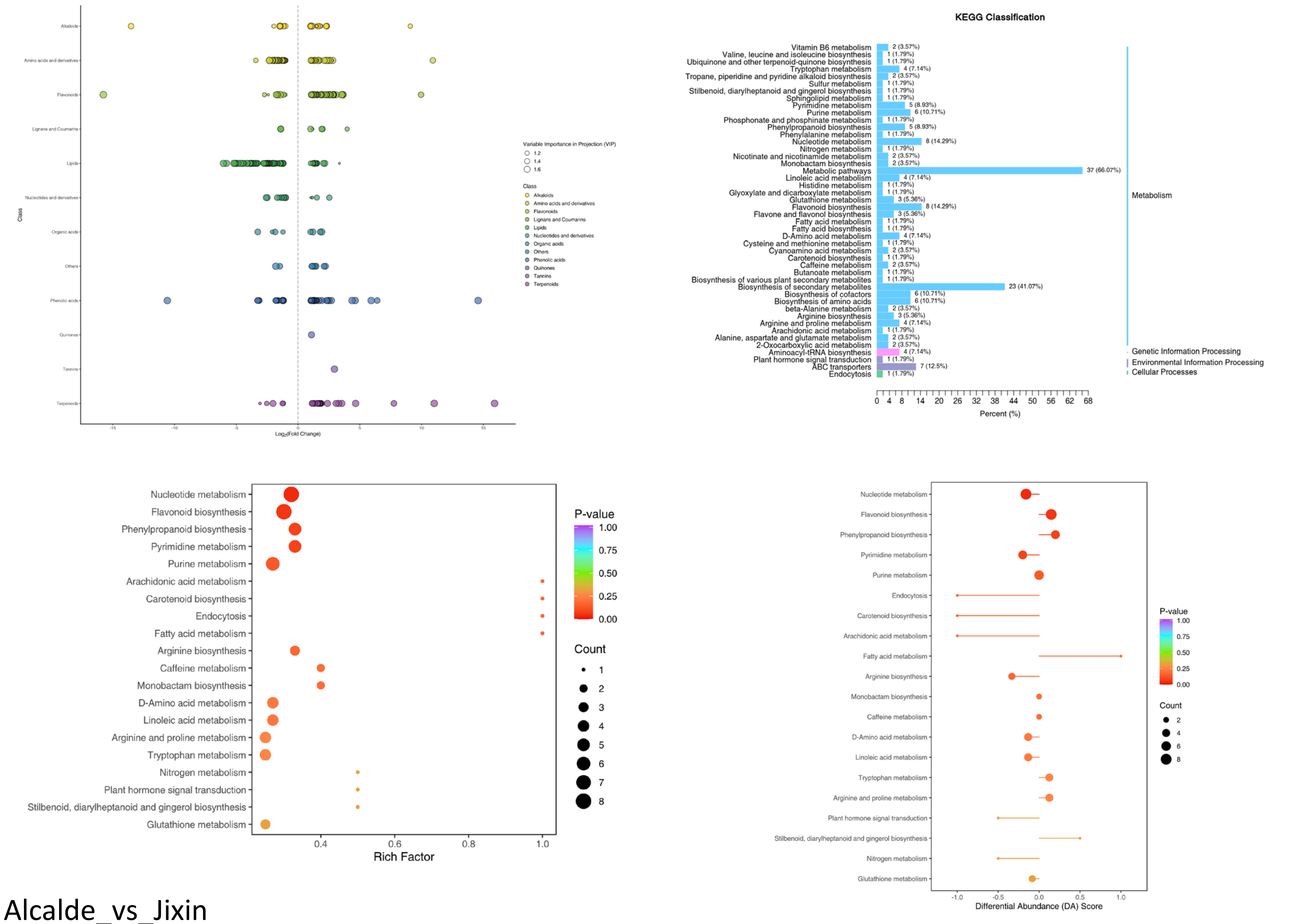


Alcalde_vs_Jixin

**Figure S5 | Pairwise differential metabolite analysis between *Ziziphus jujuba* cultivars. Scatter plot of differential metabolites shown on top, left**. Each dot in the graph indicates a metabolite, and different colors indicate different metabolite subclasses; the horizontal coordinate indicates the logarithmic value of the multiplicative difference in the content of a substance in two groups of samples (log22FC), the larger the absolute value of the horizontal coordinate, the greater the difference in the content of the substance between the two groups of samples, and the size of the dot represents the VIP value. **KEGG classification of differential metabolites on the top right**. The Y-axis shows the name of the KEGG pathway. The number of significant differential metabolites and the proportion of the total significant differential metabolites are shown next to the bar plot. **KEGG enrichment diagram of differential metabolites on bottom left.** The X-axis represents the Rich Factor and the Y-axis represents the pathway. The color of points reflects the p-value. The darker the red, the more significant the enrichment. The size of the dot represents the number of enriched differential metabolites. **Analysis of overall changes in KEGG metabolic pathway on bottom right.** The Y-axis represents the name of differential pathway, and the X-axis represents DA Score. DA Score reflects the overall change of all metabolites in the metabolic pathway. A Score of 1 indicates that the expression trend of all identified metabolites in this pathway is up-regulated, and -1 indicates that the expression trend of all identified metabolites in this pathway is down-regulated. The length of the line represent the absolute value of DA-score while the size of the dot at the end of the line represent the number of differential metabolites. A dot on the left of the line represent the pathway is down-regulated; a dot on the right of the line represents the pathway is up-regulated. The color of the line and dot represent the p-value. The darker the red, the smaller the p-value and the darker the purple, the larger the p-value.

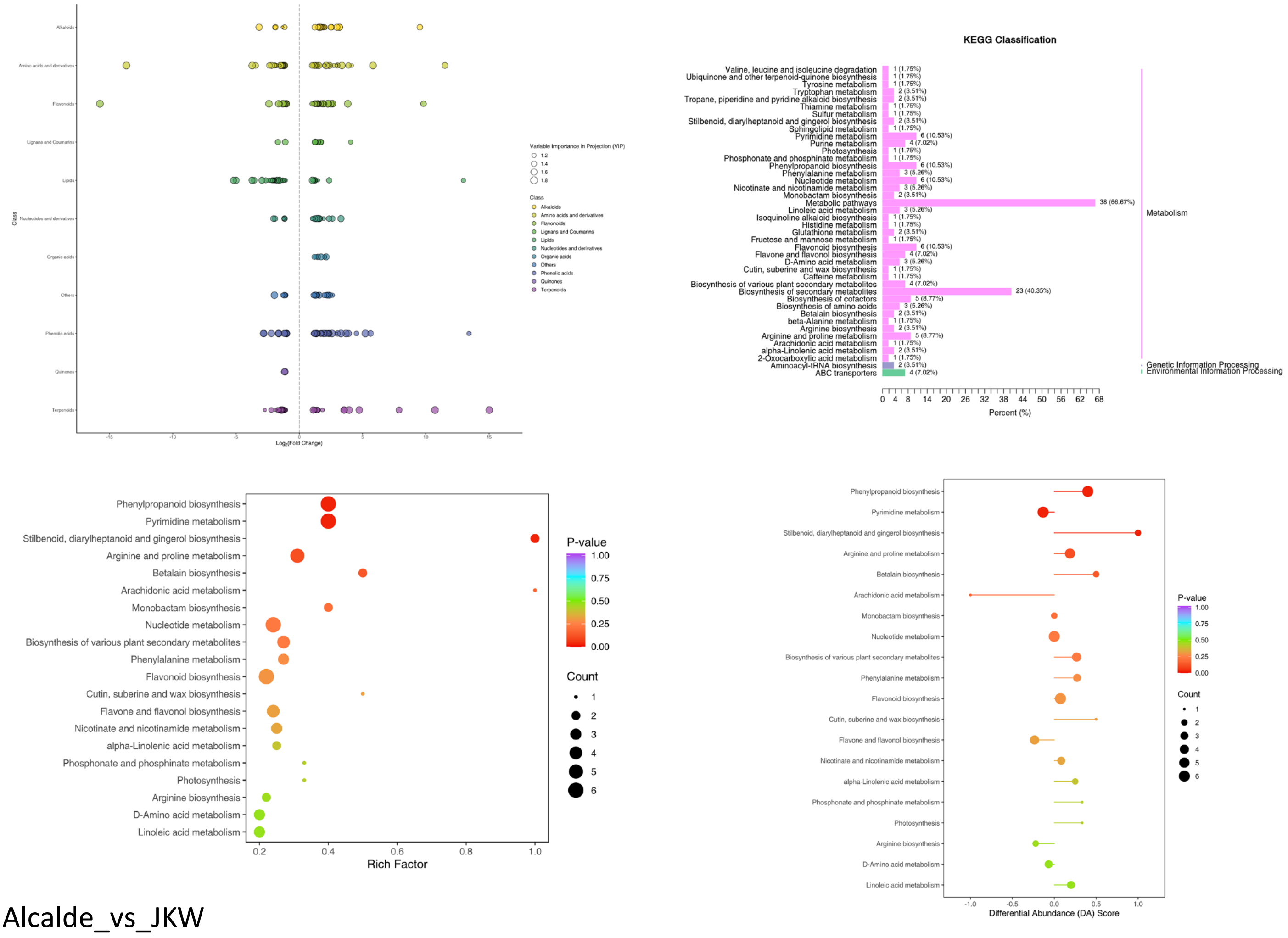


**Figure S5 | Pairwise differential metabolite analysis between *Ziziphus jujuba* cultivars. Scatter plot of differential metabolites shown on top, left**. Each dot in the graph indicates a metabolite, and different colors indicate different metabolite subclasses; the horizontal coordinate indicates the logarithmic value of the multiplicative difference in the content of a substance in two groups of samples (log22FC), the larger the absolute value of the horizontal coordinate, the greater the difference in the content of the substance between the two groups of samples, and the size of the dot represents the VIP value. **KEGG classification of differential metabolites on the top right**. The Y-axis shows the name of the KEGG pathway. The number of significant differential metabolites and the proportion of the total significant differential metabolites are shown next to the bar plot. **KEGG enrichment diagram of differential metabolites on bottom left.** The X-axis represents the Rich Factor and the Y-axis represents the pathway. The color of points reflects the p-value. The darker the red, the more significant the enrichment. The size of the dot represents the number of enriched differential metabolites. **Analysis of overall changes in KEGG metabolic pathway on bottom right.** The Y-axis represents the name of differential pathway, and the X-axis represents DA Score. DA Score reflects the overall change of all metabolites in the metabolic pathway. A Score of 1 indicates that the expression trend of all identified metabolites in this pathway is up-regulated, and -1 indicates that the expression trend of all identified metabolites in this pathway is down-regulated. The length of the line represent the absolute value of DA-score while the size of the dot at the end of the line represent the number of differential metabolites. A dot on the left of the line represent the pathway is down-regulated; a dot on the right of the line represents the pathway is up-regulated. The color of the line and dot represent the p-value. The darker the red, the smaller the p-value and the darker the purple, the larger the p-value.

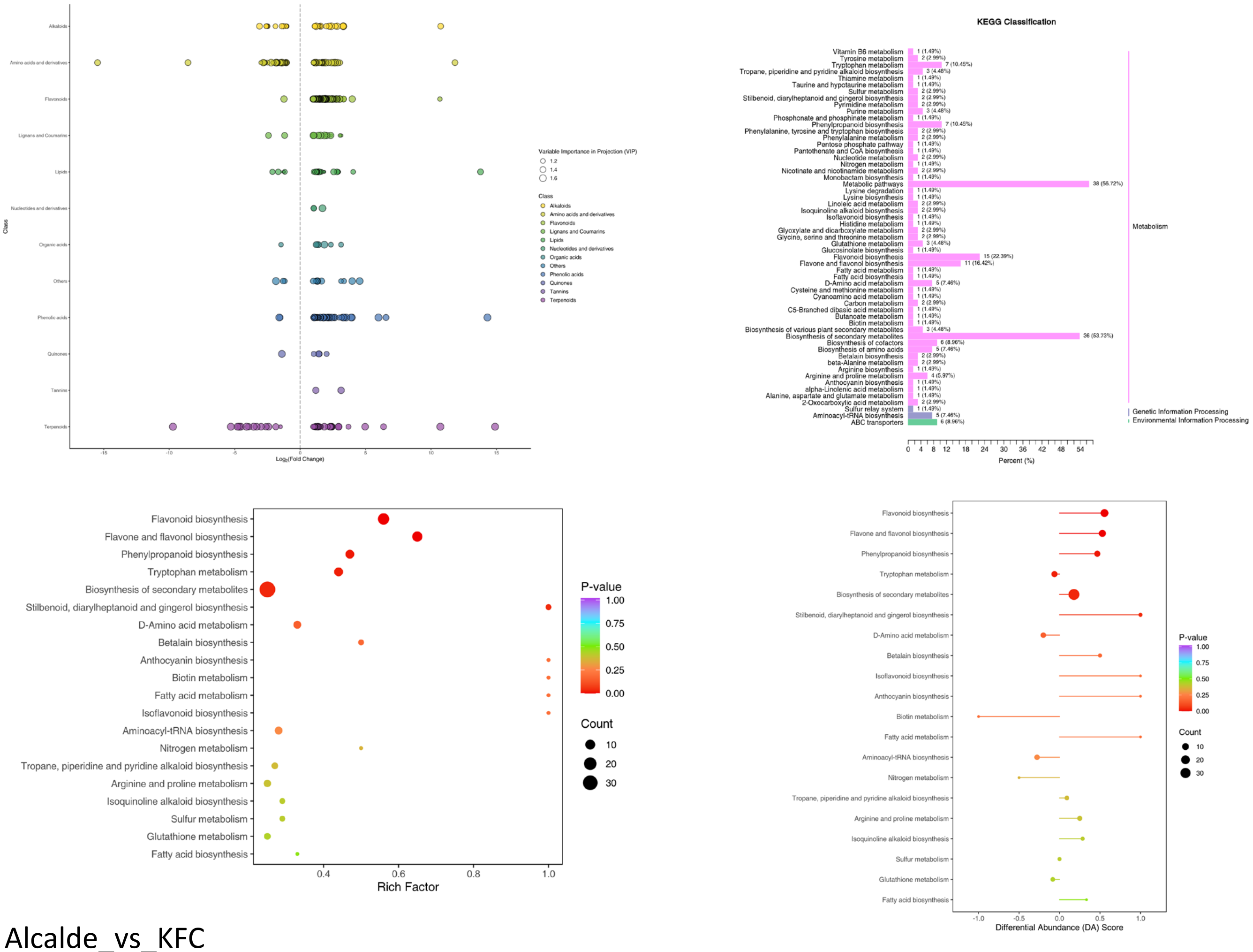


Alcalde_vs_KFC

**Figure S5 | Pairwise differential metabolite analysis between *Ziziphus jujuba* cultivars. Scatter plot of differential metabolites shown on top, left**. Each dot in the graph indicates a metabolite, and different colors indicate different metabolite subclasses; the horizontal coordinate indicates the logarithmic value of the multiplicative difference in the content of a substance in two groups of samples (log2FC), the larger the absolute value of the horizontal coordinate, the greater the difference in the content of the substance between the two groups of samples, and the size of the dot represents the VIP value. **KEGG classification of differential metabolites on the top right**. The Y-axis shows the name of the KEGG pathway. The number of significant differential metabolites and the proportion of the total significant differential metabolites are shown next to the bar plot. **KEGG enrichment diagram of differential metabolites on bottom left.** The X-axis represents the Rich Factor and the Y-axis represents the pathway. The color of points reflects the p-value. The darker the red, the more significant the enrichment. The size of the dot represents the number of enriched differential metabolites. **Analysis of overall changes in KEGG metabolic pathway on bottom right.** The Y-axis represents the name of differential pathway, and the X-axis represents DA Score. DA Score reflects the overall change of all metabolites in the metabolic pathway. A Score of 1 indicates that the expression trend of all identified metabolites in this pathway is up-regulated, and -1 indicates that the expression trend of all identified metabolites in this pathway is down-regulated. The length of the line represent the absolute value of DA-score while the size of the dot at the end of the line represent the number of differential metabolites. A dot on the left of the line represent the pathway is down-regulated; a dot on the right of the line represents the pathway is up-regulated. The color of the line and dot represent the p-value. The darker the red, the smaller the p-value and the darker the purple, the larger the p-value.

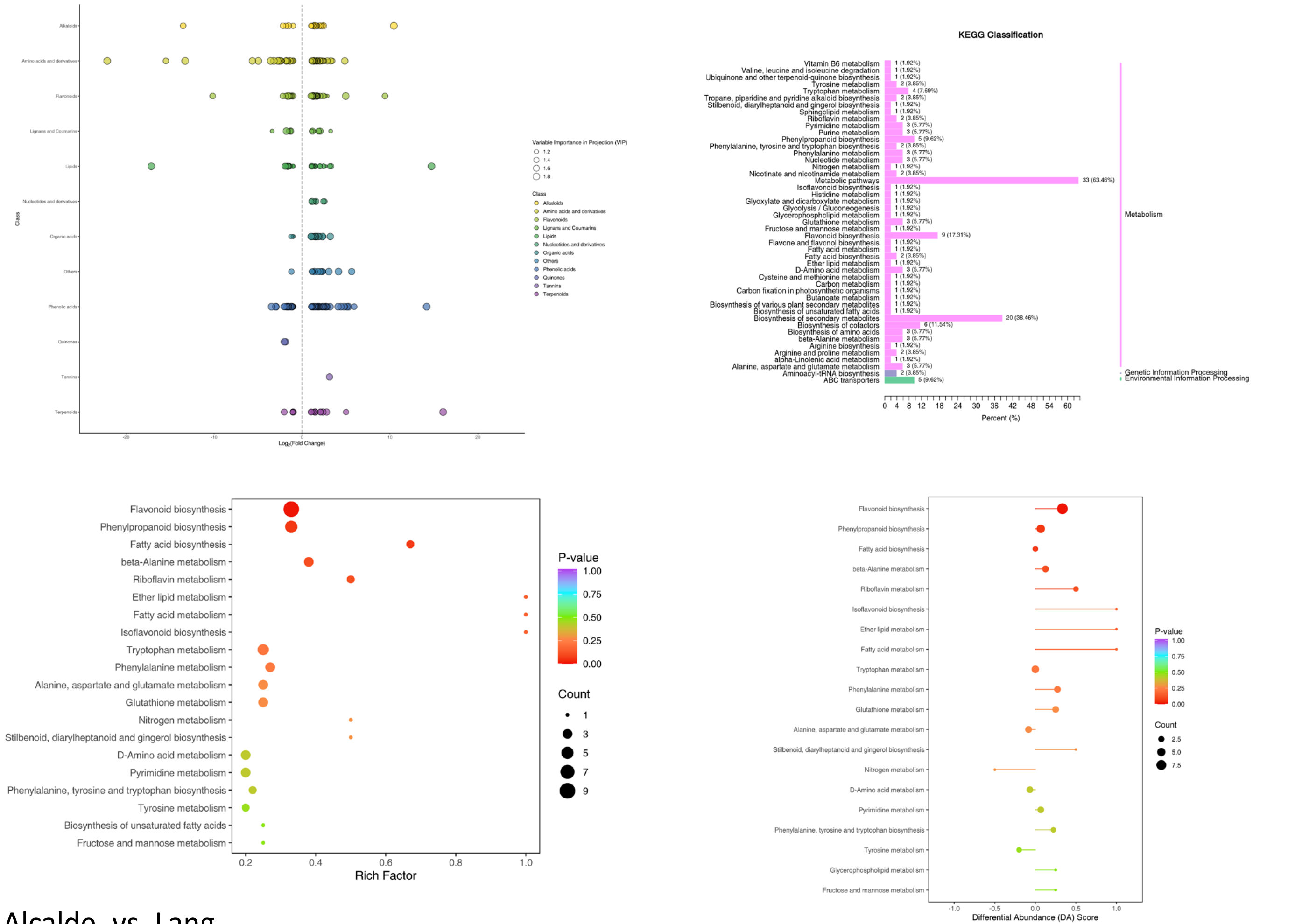


Alcalde_vs_Lang

**Figure S5 | Pairwise differential metabolite analysis between *Ziziphus jujuba* cultivars. Scatter plot of differential metabolites shown on top, left**. Each dot in the graph indicates a metabolite, and different colors indicate different metabolite subclasses; the horizontal coordinate indicates the logarithmic value of the multiplicative difference in the content of a substance in two groups of samples (log22FC), the larger the absolute value of the horizontal coordinate, the greater the difference in the content of the substance between the two groups of samples, and the size of the dot represents the VIP value. **KEGG classification of differential metabolites on the top right**. The Y-axis shows the name of the KEGG pathway. The number of significant differential metabolites and the proportion of the total significant differential metabolites are shown next to the bar plot. **KEGG enrichment diagram of differential metabolites on bottom left.** The X-axis represents the Rich Factor and the Y-axis represents the pathway. The color of points reflects the p-value. The darker the red, the more significant the enrichment. The size of the dot represents the number of enriched differential metabolites. **Analysis of overall changes in KEGG metabolic pathway on bottom right.** The Y-axis represents the name of differential pathway, and the X-axis represents DA Score. DA Score reflects the overall change of all metabolites in the metabolic pathway. A Score of 1 indicates that the expression trend of all identified metabolites in this pathway is up-regulated, and -1 indicates that the expression trend of all identified metabolites in this pathway is down-regulated. The length of the line represent the absolute value of DA-score while the size of the dot at the end of the line represent the number of differential metabolites. A dot on the left of the line represent the pathway is down-regulated; a dot on the right of the line represents the pathway is up-regulated. The color of the line and dot represent the p-value. The darker the red, the smaller the p-value and the darker the purple, the larger the p-value.

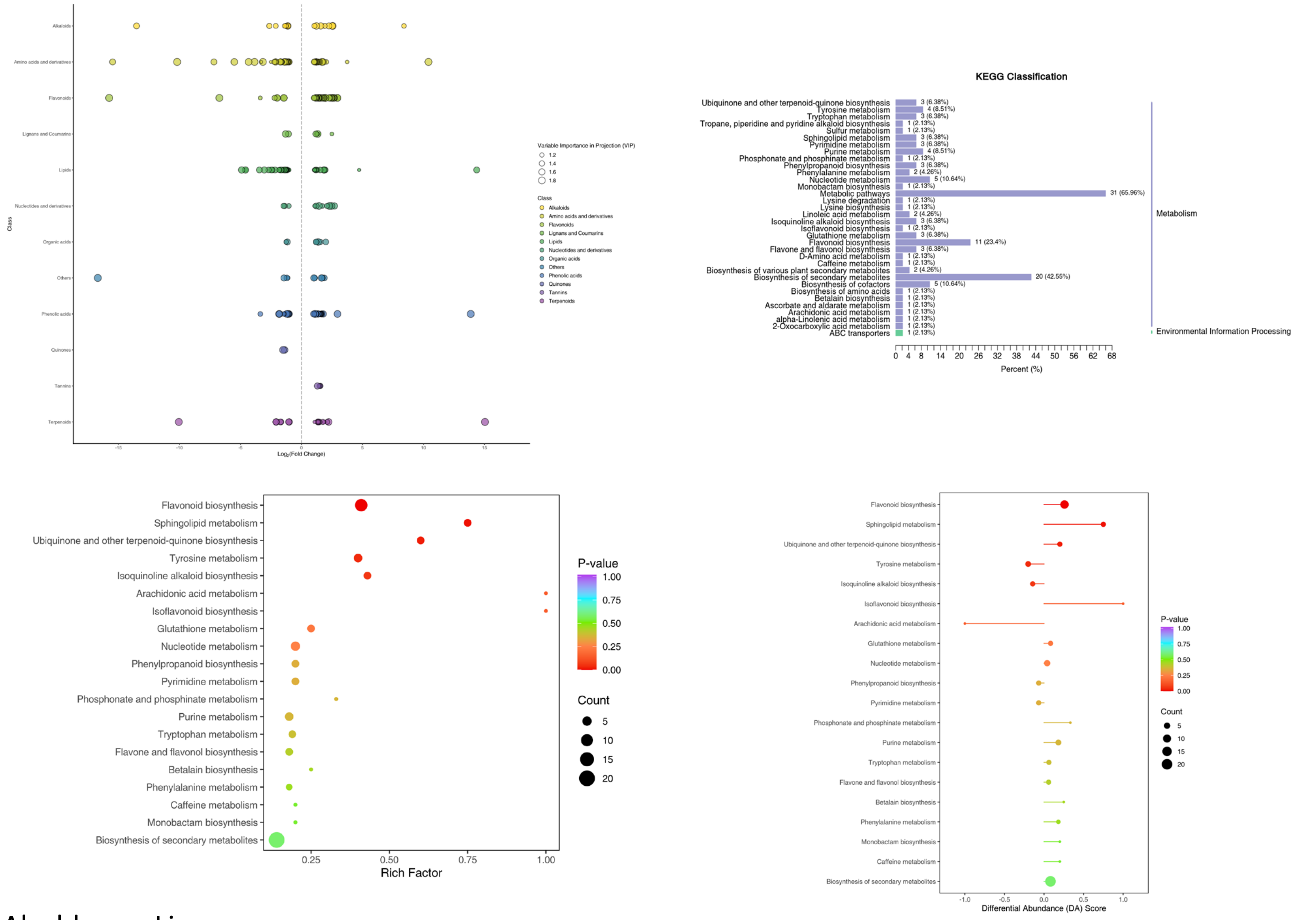


Alcalde_vs_Li

**Figure S5 | Pairwise differential metabolite analysis between *Ziziphus jujuba* cultivars. Scatter plot of differential metabolites shown on top, left**. Each dot in the graph indicates a metabolite, and different colors indicate different metabolite subclasses; the horizontal coordinate indicates the logarithmic value of the multiplicative difference in the content of a substance in two groups of samples (log$_2$2FC), the larger the absolute value of the horizontal coordinate, the greater the difference in the content of the substance between the two groups of samples, and the size of the dot represents the VIP value. **KEGG classification of differential metabolites on the top right**. The Y-axis shows the name of the KEGG pathway. The number of significant differential metabolites and the proportion of the total significant differential metabolites are shown next to the bar plot. **KEGG enrichment diagram of differential metabolites on bottom left.** The X-axis represents the Rich Factor and the Y-axis represents the pathway. The color of points reflects the p-value. The darker the red, the more significant the enrichment. The size of the dot represents the number of enriched differential metabolites. **Analysis of overall changes in KEGG metabolic pathway on bottom right.** The Y-axis represents the name of differential pathway, and the X-axis represents DA Score. DA Score reflects the overall change of all metabolites in the metabolic pathway. A Score of 1 indicates that the expression trend of all identified metabolites in this pathway is up-regulated, and -1 indicates that the expression trend of all identified metabolites in this pathway is down-regulated. The length of the line represent the absolute value of DA-score while the size of the dot at the end of the line represent the number of differential metabolites. A dot on the left of the line represent the pathway is down-regulated; a dot on the right of the line represents the pathway is up-regulated. The color of the line and dot represent the p-value. The darker the red, the smaller the p-value and the darker the purple, the larger the p-value.

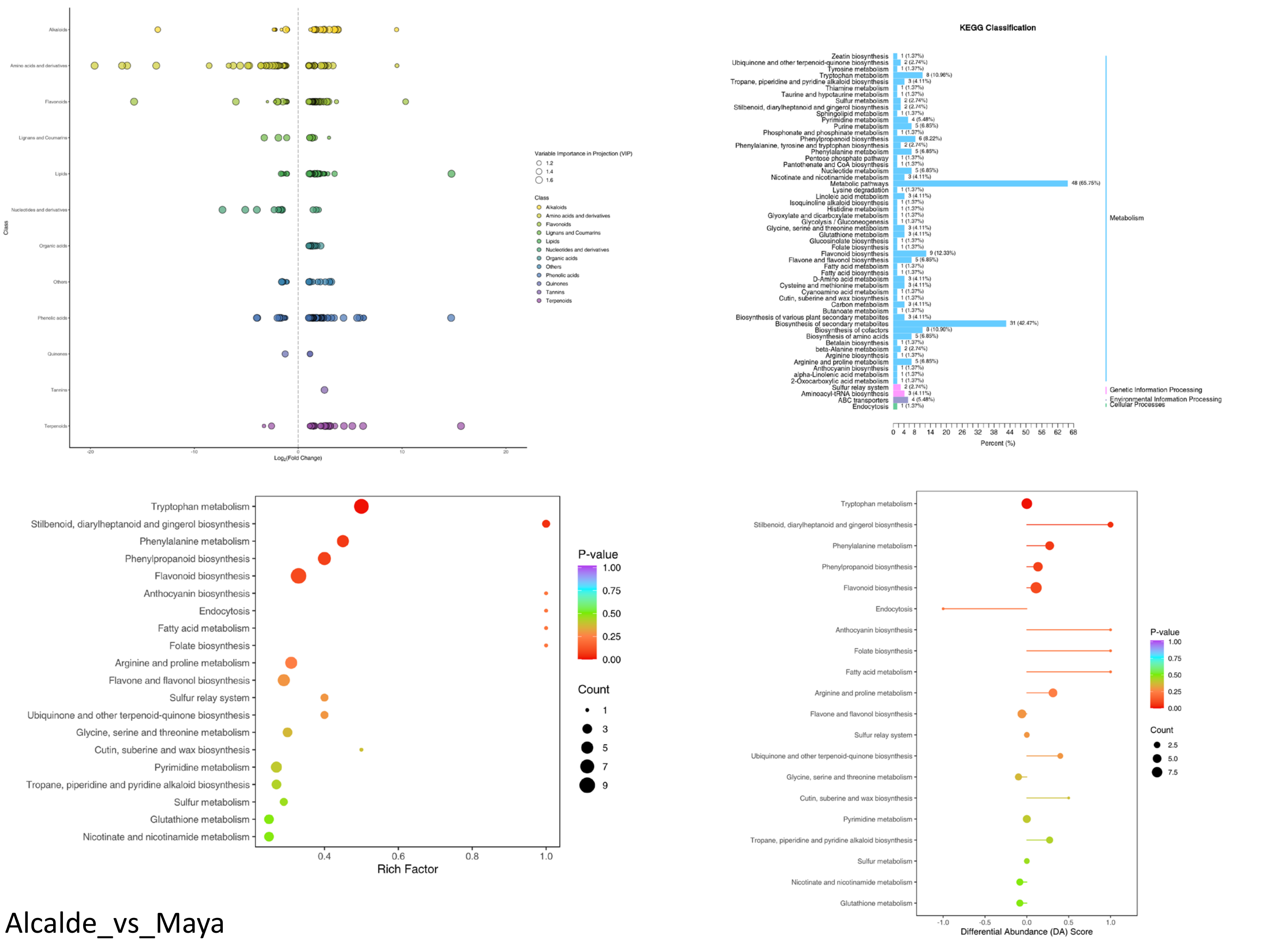


Alcalde_vs_Maya

**Figure S5 | Pairwise differential metabolite analysis between *Ziziphus jujuba* cultivars. Scatter plot of differential metabolites shown on top, left**. Each dot in the graph indicates a metabolite, and different colors indicate different metabolite subclasses; the horizontal coordinate indicates the logarithmic value of the multiplicative difference in the content of a substance in two groups of samples (log22FC), the larger the absolute value of the horizontal coordinate, the greater the difference in the content of the substance between the two groups of samples, and the size of the dot represents the VIP value. **KEGG classification of differential metabolites on the top right**. The Y-axis shows the name of the KEGG pathway. The number of significant differential metabolites and the proportion of the total significant differential metabolites are shown next to the bar plot. **KEGG enrichment diagram of differential metabolites on bottom left.** The X-axis represents the Rich Factor and the Y-axis represents the pathway. The color of points reflects the p-value. The darker the red, the more significant the enrichment. The size of the dot represents the number of enriched differential metabolites. **Analysis of overall changes in KEGG metabolic pathway on bottom right.** The Y-axis represents the name of differential pathway, and the X-axis represents DA Score. DA Score reflects the overall change of all metabolites in the metabolic pathway. A Score of 1 indicates that the expression trend of all identified metabolites in this pathway is up-regulated, and -1 indicates that the expression trend of all identified metabolites in this pathway is down-regulated. The length of the line represent the absolute value of DA-score while the size of the dot at the end of the line represent the number of differential metabolites. A dot on the left of the line represent the pathway is down-regulated; a dot on the right of the line represents the pathway is up-regulated. The color of the line and dot represent the p-value. The darker the red, the smaller the p-value and the darker the purple, the larger the p-value.

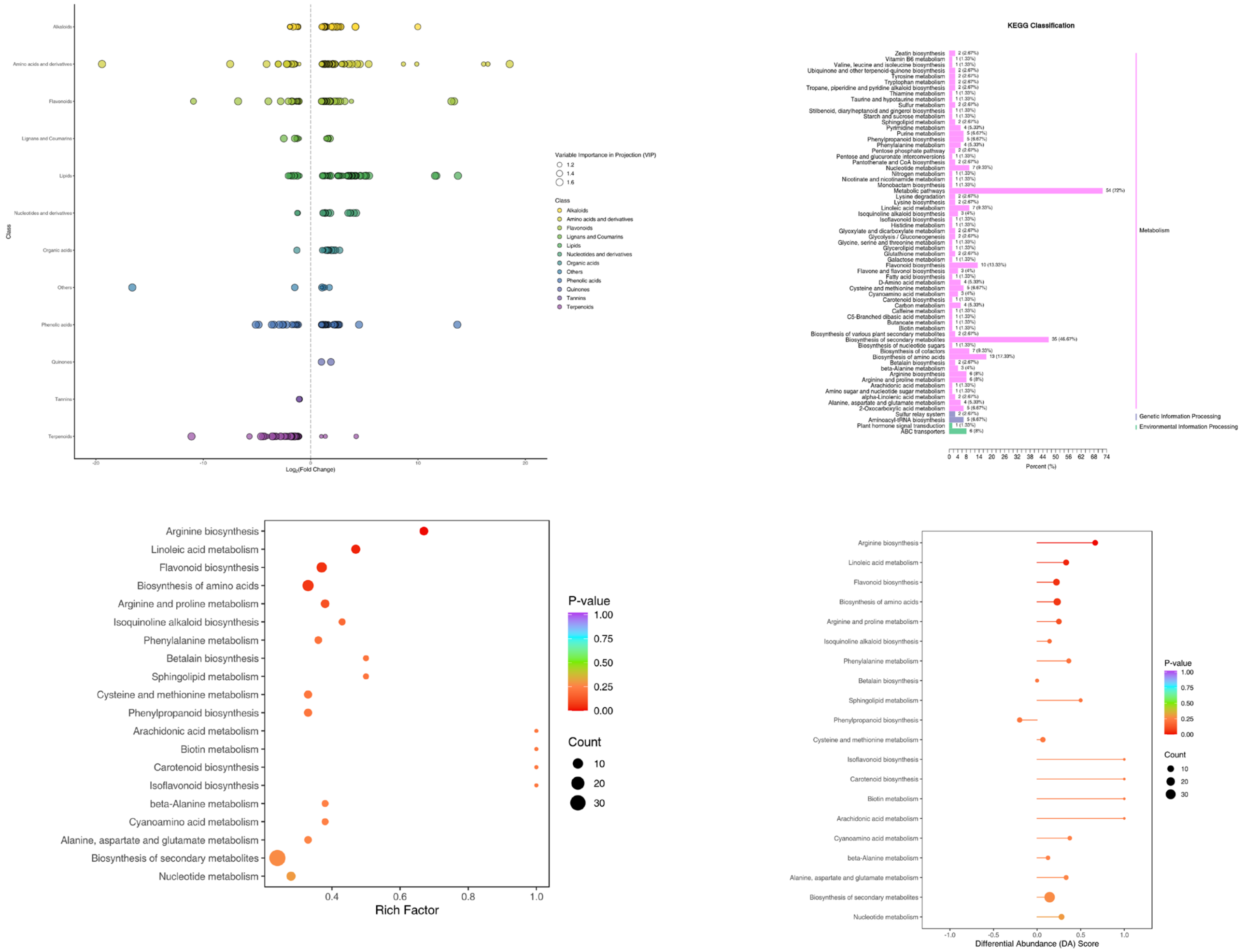


Sandia (Hebei Dongzao)_vs_Shanxi Li

**Figure S5 | Pairwise differential metabolite analysis between *Ziziphus jujuba* cultivars. Scatter plot of differential metabolites shown on top, left**. Each dot in the graph indicates a metabolite, and different colors indicate different metabolite subclasses; the horizontal coordinate indicates the logarithmic value of the multiplicative difference in the content of a substance in two groups of samples (log22FC), the larger the absolute value of the horizontal coordinate, the greater the difference in the content of the substance between the two groups of samples, and the size of the dot represents the VIP value. **KEGG classification of differential metabolites on the top right**. The Y-axis shows the name of the KEGG pathway. The number of significant differential metabolites and the proportion of the total significant differential metabolites are shown next to the bar plot. **KEGG enrichment diagram of differential metabolites on bottom left.** The X-axis represents the Rich Factor and the Y-axis represents the pathway. The color of points reflects the p-value. The darker the red, the more significant the enrichment. The size of the dot represents the number of enriched differential metabolites. **Analysis of overall changes in KEGG metabolic pathway on bottom right.** The Y-axis represents the name of differential pathway, and the X-axis represents DA Score. DA Score reflects the overall change of all metabolites in the metabolic pathway. A Score of 1 indicates that the expression trend of all identified metabolites in this pathway is up-regulated, and -1 indicates that the expression trend of all identified metabolites in this pathway is down-regulated. The length of the line represent the absolute value of DA-score while the size of the dot at the end of the line represent the number of differential metabolites. A dot on the left of the line represent the pathway is down-regulated; a dot on the right of the line represents the pathway is up-regulated. The color of the line and dot represent the p-value. The darker the red, the smaller the p-value and the darker the purple, the larger the p-value.

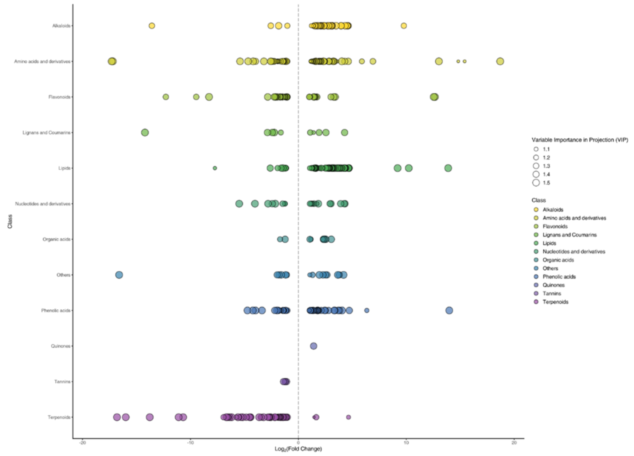


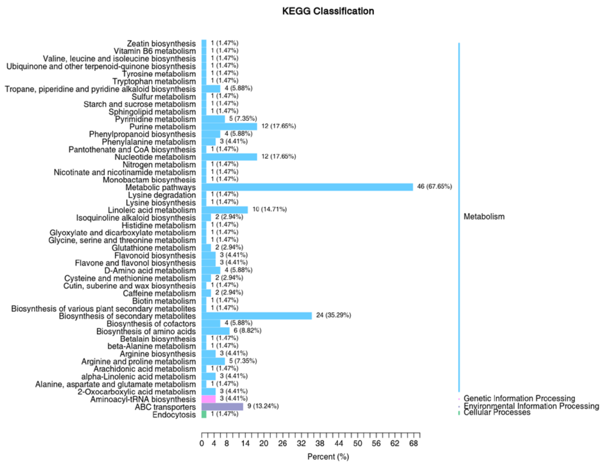


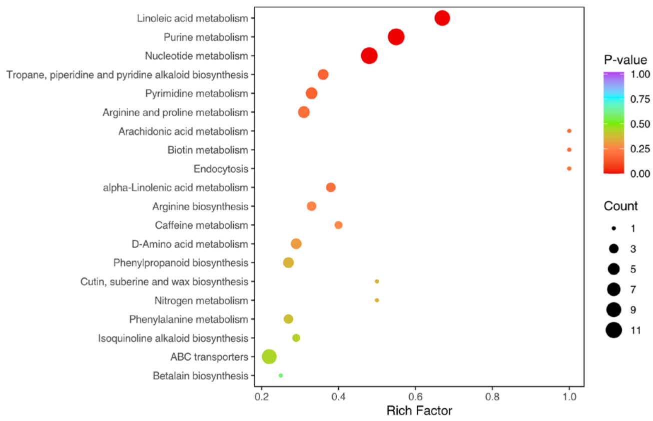


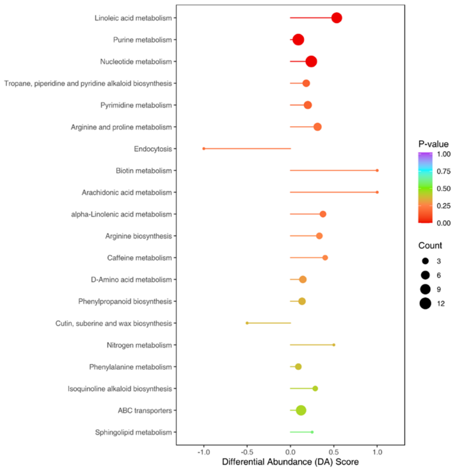


Sandia (Hebei Dongzao)_vs_ZCW

**Figure S5 | Pairwise differential metabolite analysis between *Ziziphus jujuba* cultivars. Scatter plot of differential metabolites shown on top, left**. Each dot in the graph indicates a metabolite, and different colors indicate different metabolite subclasses; the horizontal coordinate indicates the logarithmic value of the multiplicative difference in the content of a substance in two groups of samples (log22FC), the larger the absolute value of the horizontal coordinate, the greater the difference in the content of the substance between the two groups of samples, and the size of the dot represents the VIP value. **KEGG classification of differential metabolites on the top right**. The Y-axis shows the name of the KEGG pathway. The number of significant differential metabolites and the proportion of the total significant differential metabolites are shown next to the bar plot. **KEGG enrichment diagram of differential metabolites on bottom left.** The X-axis represents the Rich Factor and the Y-axis represents the pathway. The color of points reflects the p-value. The darker the red, the more significant the enrichment. The size of the dot represents the number of enriched differential metabolites. **Analysis of overall changes in KEGG metabolic pathway on bottom right.** The Y-axis represents the name of differential pathway, and the X-axis represents DA Score. DA Score reflects the overall change of all metabolites in the metabolic pathway. A Score of 1 indicates that the expression trend of all identified metabolites in this pathway is up-regulated, and -1 indicates that the expression trend of all identified metabolites in this pathway is down-regulated. The length of the line represent the absolute value of DA-score while the size of the dot at the end of the line represent the number of differential metabolites. A dot on the left of the line represent the pathway is down-regulated; a dot on the right of the line represents the pathway is up-regulated. The color of the line and dot represent the p-value. The darker the red, the smaller the p-value and the darker the purple, the larger the p-value.

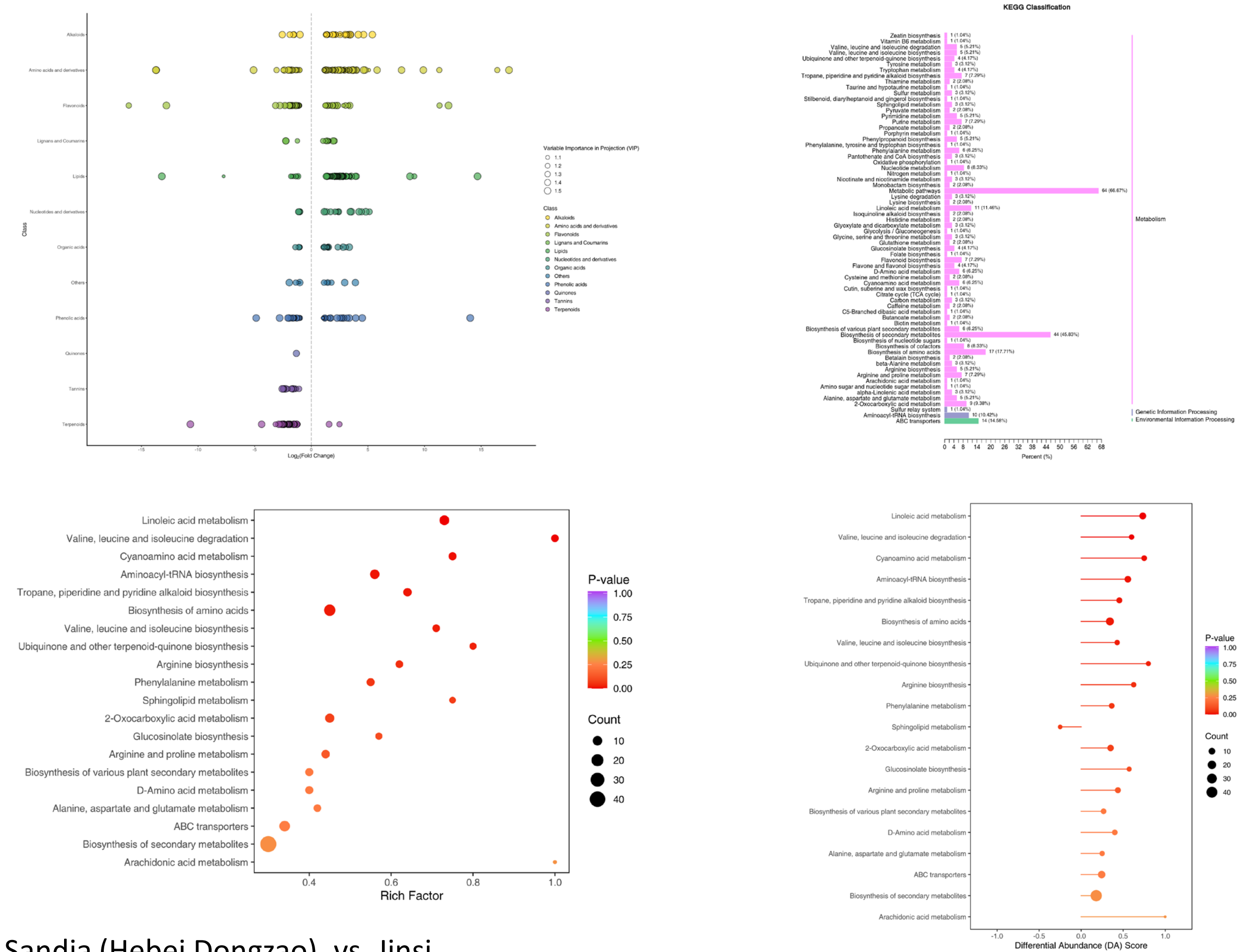


Sandia (Hebei Dongzao)_vs_Jinsi

**Figure S5 | Pairwise differential metabolite analysis between *Ziziphus jujuba* cultivars. Scatter plot of differential metabolites shown on top, left**. Each dot in the graph indicates a metabolite, and different colors indicate different metabolite subclasses; the horizontal coordinate indicates the logarithmic value of the multiplicative difference in the content of a substance in two groups of samples (log22FC), the larger the absolute value of the horizontal coordinate, the greater the difference in the content of the substance between the two groups of samples, and the size of the dot represents the VIP value. **KEGG classification of differential metabolites on the top right**. The Y-axis shows the name of the KEGG pathway. The number of significant differential metabolites and the proportion of the total significant differential metabolites are shown next to the bar plot. **KEGG enrichment diagram of differential metabolites on bottom left.** The X-axis represents the Rich Factor and the Y-axis represents the pathway. The color of points reflects the p-value. The darker the red, the more significant the enrichment. The size of the dot represents the number of enriched differential metabolites. **Analysis of overall changes in KEGG metabolic pathway on bottom right.** The Y-axis represents the name of differential pathway, and the X-axis represents DA Score. DA Score reflects the overall change of all metabolites in the metabolic pathway. A Score of 1 indicates that the expression trend of all identified metabolites in this pathway is up-regulated, and -1 indicates that the expression trend of all identified metabolites in this pathway is down-regulated. The length of the line represent the absolute value of DA-score while the size of the dot at the end of the line represent the number of differential metabolites. A dot on the left of the line represent the pathway is down-regulated; a dot on the right of the line represents the pathway is up-regulated. The color of the line and dot represent the p-value. The darker the red, the smaller the p-value and the darker the purple, the larger the p-value.

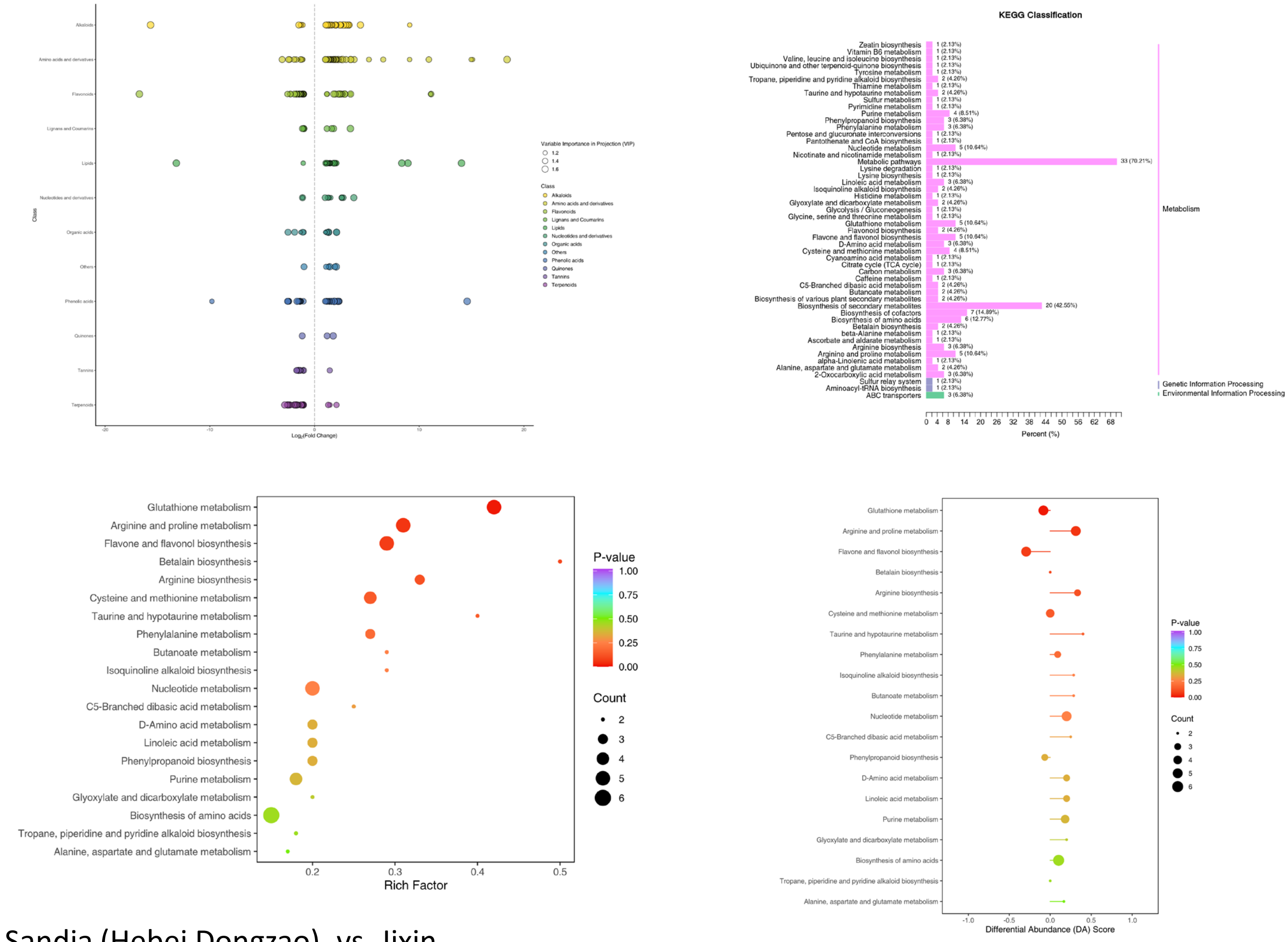


Sandia (Hebei Dongzao)_vs_Jixin

**Figure S5 | Pairwise differential metabolite analysis between *Ziziphus jujuba* cultivars. Scatter plot of differential metabolites shown on top, left**. Each dot in the graph indicates a metabolite, and different colors indicate different metabolite subclasses; the horizontal coordinate indicates the logarithmic value of the multiplicative difference in the content of a substance in two groups of samples ($log_2$2FC), the larger the absolute value of the horizontal coordinate, the greater the difference in the content of the substance between the two groups of samples, and the size of the dot represents the VIP value. **KEGG classification of differential metabolites on the top right**. The Y-axis shows the name of the KEGG pathway. The number of significant differential metabolites and the proportion of the total significant differential metabolites are shown next to the bar plot. **KEGG enrichment diagram of differential metabolites on bottom left.** The X-axis represents the Rich Factor and the Y-axis represents the pathway. The color of points reflects the p-value. The darker the red, the more significant the enrichment. The size of the dot represents the number of enriched differential metabolites. **Analysis of overall changes in KEGG metabolic pathway on bottom right.** The Y-axis represents the name of differential pathway, and the X-axis represents DA Score. DA Score reflects the overall change of all metabolites in the metabolic pathway. A Score of 1 indicates that the expression trend of all identified metabolites in this pathway is up-regulated, and -1 indicates that the expression trend of all identified metabolites in this pathway is down-regulated. The length of the line represent the absolute value of DA-score while the size of the dot at the end of the line represent the number of differential metabolites. A dot on the left of the line represent the pathway is down-regulated; a dot on the right of the line represents the pathway is up-regulated. The color of the line and dot represent the p-value. The darker the red, the smaller the p-value and the darker the purple, the larger the p-value.

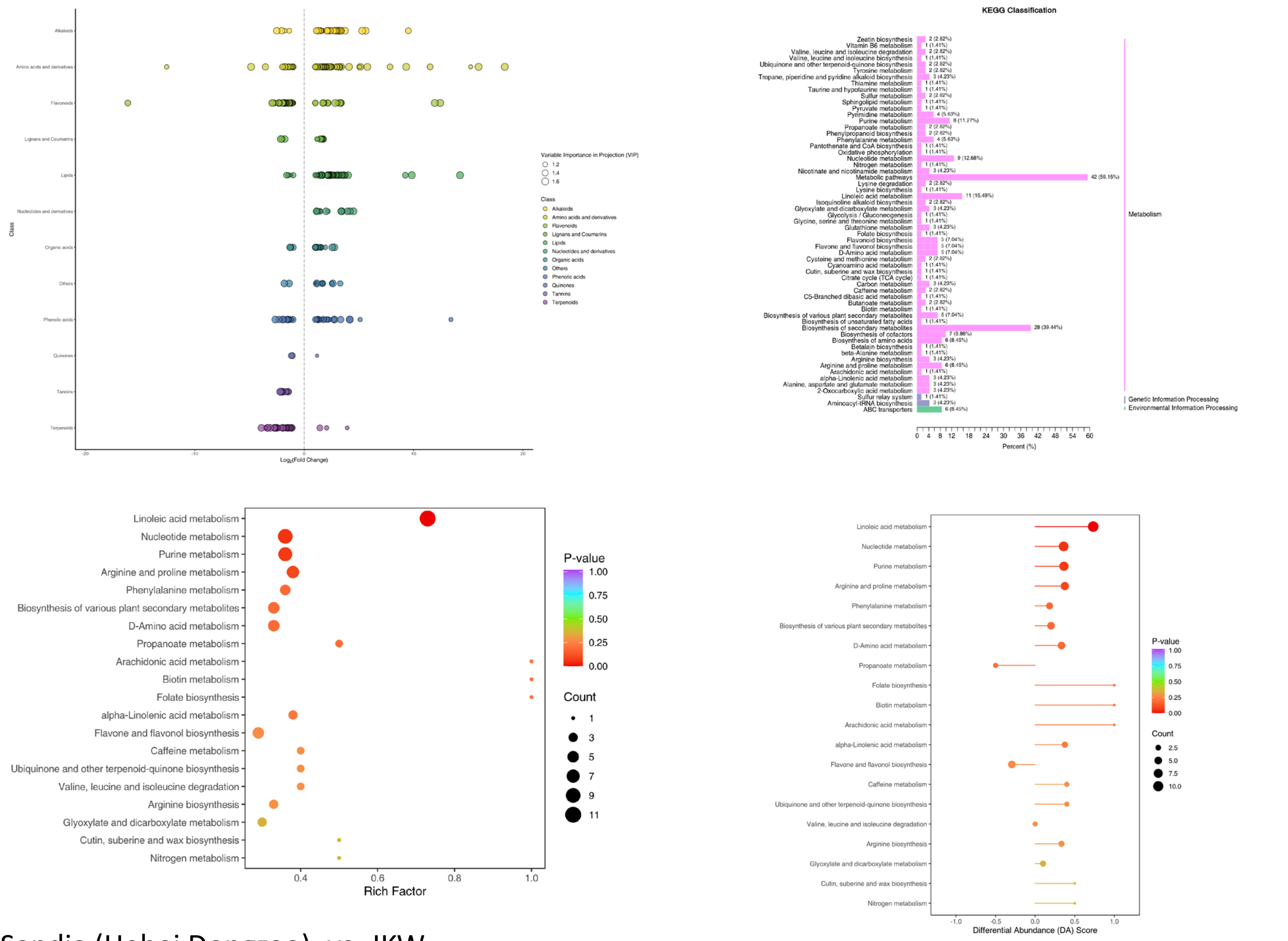


Sandia (Hebei Dongzao)_vs_JKW

**Figure S5 | Pairwise differential metabolite analysis between *Ziziphus jujuba* cultivars. Scatter plot of differential metabolites shown on top, left**. Each dot in the graph indicates a metabolite, and different colors indicate different metabolite subclasses; the horizontal coordinate indicates the logarithmic value of the multiplicative difference in the content of a substance in two groups of samples (log22FC), the larger the absolute value of the horizontal coordinate, the greater the difference in the content of the substance between the two groups of samples, and the size of the dot represents the VIP value. **KEGG classification of differential metabolites on the top right**. The Y-axis shows the name of the KEGG pathway. The number of significant differential metabolites and the proportion of the total significant differential metabolites are shown next to the bar plot. **KEGG enrichment diagram of differential metabolites on bottom left.** The X-axis represents the Rich Factor and the Y-axis represents the pathway. The color of points reflects the p-value. The darker the red, the more significant the enrichment. The size of the dot represents the number of enriched differential metabolites. **Analysis of overall changes in KEGG metabolic pathway on bottom right.** The Y-axis represents the name of differential pathway, and the X-axis represents DA Score. DA Score reflects the overall change of all metabolites in the metabolic pathway. A Score of 1 indicates that the expression trend of all identified metabolites in this pathway is up-regulated, and -1 indicates that the expression trend of all identified metabolites in this pathway is down-regulated. The length of the line represent the absolute value of DA-score while the size of the dot at the end of the line represent the number of differential metabolites. A dot on the left of the line represent the pathway is down-regulated; a dot on the right of the line represents the pathway is up-regulated. The color of the line and dot represent the p-value. The darker the red, the smaller the p-value and the darker the purple, the larger the p-value.

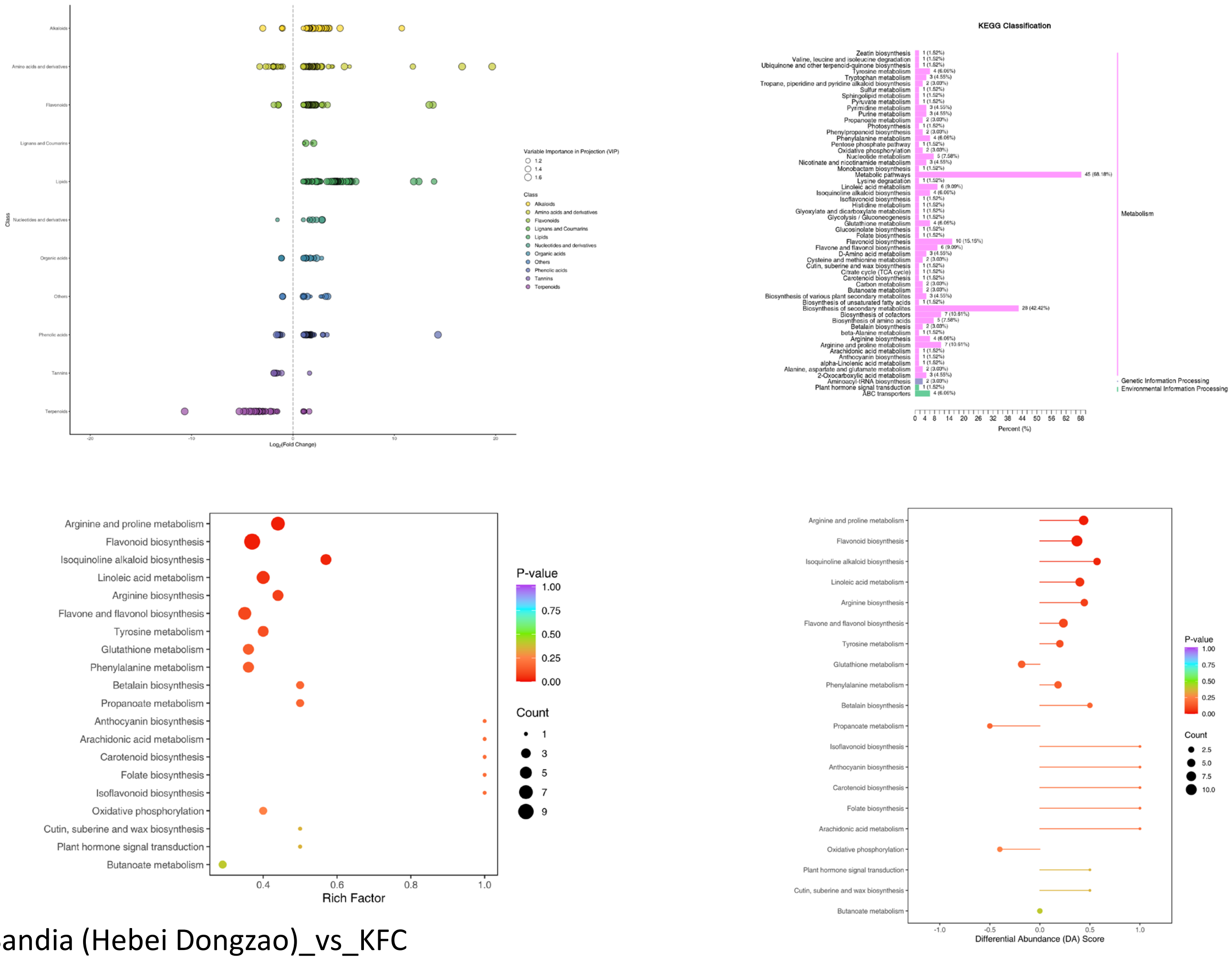


Sandia (Hebei Dongzao)_vs_KFC

**Figure S5 | Pairwise differential metabolite analysis between *Ziziphus jujuba* cultivars. Scatter plot of differential metabolites shown on top, left**. Each dot in the graph indicates a metabolite, and different colors indicate different metabolite subclasses; the horizontal coordinate indicates the logarithmic value of the multiplicative difference in the content of a substance in two groups of samples (log22FC), the larger the absolute value of the horizontal coordinate, the greater the difference in the content of the substance between the two groups of samples, and the size of the dot represents the VIP value. **KEGG classification of differential metabolites on the top right**. The Y-axis shows the name of the KEGG pathway. The number of significant differential metabolites and the proportion of the total significant differential metabolites are shown next to the bar plot. **KEGG enrichment diagram of differential metabolites on bottom left.** The X-axis represents the Rich Factor and the Y-axis represents the pathway. The color of points reflects the p-value. The darker the red, the more significant the enrichment. The size of the dot represents the number of enriched differential metabolites. **Analysis of overall changes in KEGG metabolic pathway on bottom right.** The Y-axis represents the name of differential pathway, and the X-axis represents DA Score. DA Score reflects the overall change of all metabolites in the metabolic pathway. A Score of 1 indicates that the expression trend of all identified metabolites in this pathway is up-regulated, and -1 indicates that the expression trend of all identified metabolites in this pathway is down-regulated. The length of the line represent the absolute value of DA-score while the size of the dot at the end of the line represent the number of differential metabolites. A dot on the left of the line represent the pathway is down-regulated; a dot on the right of the line represents the pathway is up-regulated. The color of the line and dot represent the p-value. The darker the red, the smaller the p-value and the darker the purple, the larger the p-value.

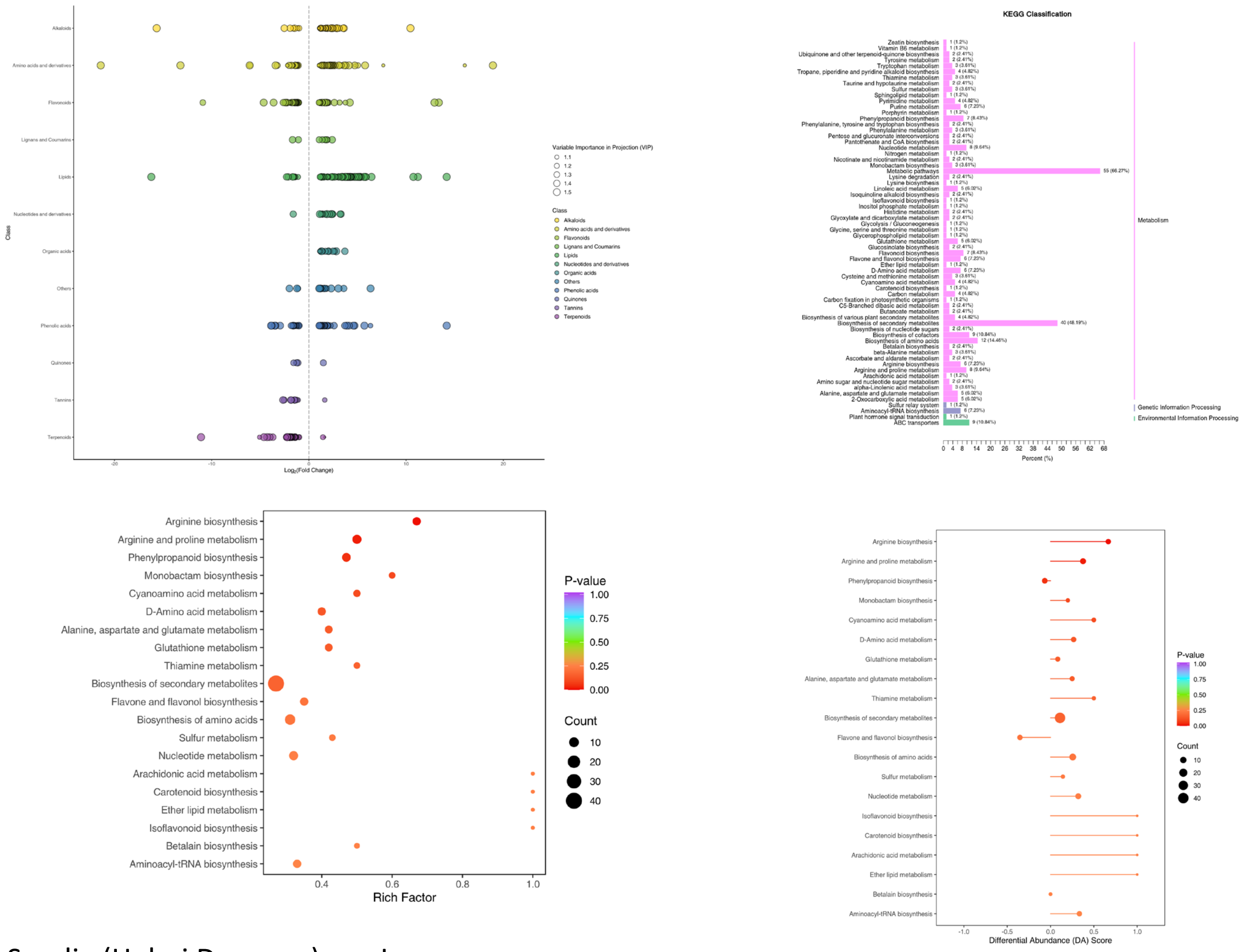


Sandia (Hebei Dongzao)_vs_Lang

**Figure S5 | Pairwise differential metabolite analysis between *Ziziphus jujuba* cultivars. Scatter plot of differential metabolites shown on top, left**. Each dot in the graph indicates a metabolite, and different colors indicate different metabolite subclasses; the horizontal coordinate indicates the logarithmic value of the multiplicative difference in the content of a substance in two groups of samples (log22FC), the larger the absolute value of the horizontal coordinate, the greater the difference in the content of the substance between the two groups of samples, and the size of the dot represents the VIP value. **KEGG classification of differential metabolites on the top right**. The Y-axis shows the name of the KEGG pathway. The number of significant differential metabolites and the proportion of the total significant differential metabolites are shown next to the bar plot. **KEGG enrichment diagram of differential metabolites on bottom left.** The X-axis represents the Rich Factor and the Y-axis represents the pathway. The color of points reflects the p-value. The darker the red, the more significant the enrichment. The size of the dot represents the number of enriched differential metabolites. **Analysis of overall changes in KEGG metabolic pathway on bottom right.** The Y-axis represents the name of differential pathway, and the X-axis represents DA Score. DA Score reflects the overall change of all metabolites in the metabolic pathway. A Score of 1 indicates that the expression trend of all identified metabolites in this pathway is up-regulated, and -1 indicates that the expression trend of all identified metabolites in this pathway is down-regulated. The length of the line represent the absolute value of DA-score while the size of the dot at the end of the line represent the number of differential metabolites. A dot on the left of the line represent the pathway is down-regulated; a dot on the right of the line represents the pathway is up-regulated. The color of the line and dot represent the p-value. The darker the red, the smaller the p-value and the darker the purple, the larger the p-value.

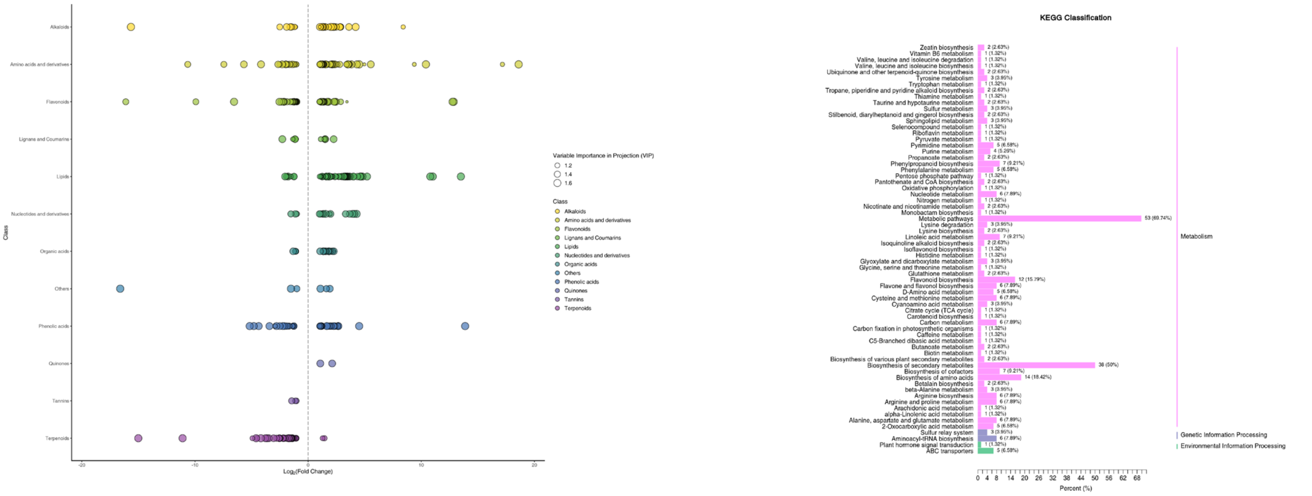


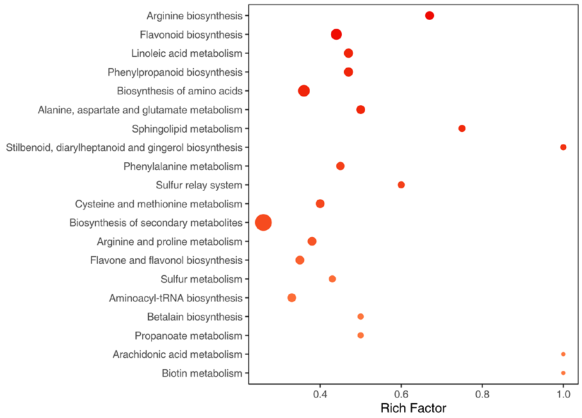


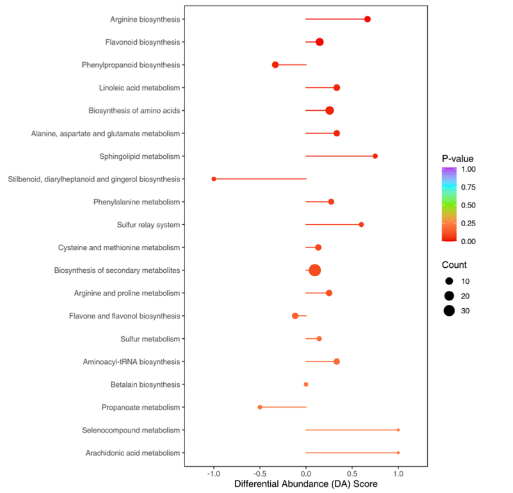


Sandia (Hebei Dongzao)_vs_Li

**Figure S5 | Pairwise differential metabolite analysis between *Ziziphus jujuba* cultivars. Scatter plot of differential metabolites shown on top, left**. Each dot in the graph indicates a metabolite, and different colors indicate different metabolite subclasses; the horizontal coordinate indicates the logarithmic value of the multiplicative difference in the content of a substance in two groups of samples ($log_22FC$), the larger the absolute value of the horizontal coordinate, the greater the difference in the content of the substance between the two groups of samples, and the size of the dot represents the VIP value. **KEGG classification of differential metabolites on the top right**. The Y-axis shows the name of the KEGG pathway. The number of significant differential metabolites and the proportion of the total significant differential metabolites are shown next to the bar plot. **KEGG enrichment diagram of differential metabolites on bottom left.** The X-axis represents the Rich Factor and the Y-axis represents the pathway. The color of points reflects the p-value. The darker the red, the more significant the enrichment. The size of the dot represents the number of enriched differential metabolites. **Analysis of overall changes in KEGG metabolic pathway on bottom right.** The Y-axis represents the name of differential pathway, and the X-axis represents DA Score. DA Score reflects the overall change of all metabolites in the metabolic pathway. A Score of 1 indicates that the expression trend of all identified metabolites in this pathway is up-regulated, and -1 indicates that the expression trend of all identified metabolites in this pathway is down-regulated. The length of the line represent the absolute value of DA-score while the size of the dot at the end of the line represent the number of differential metabolites. A dot on the left of the line represent the pathway is down-regulated; a dot on the right of the line represents the pathway is up-regulated. The color of the line and dot represent the p-value. The darker the red, the smaller the p-value and the darker the purple, the larger the p-value.

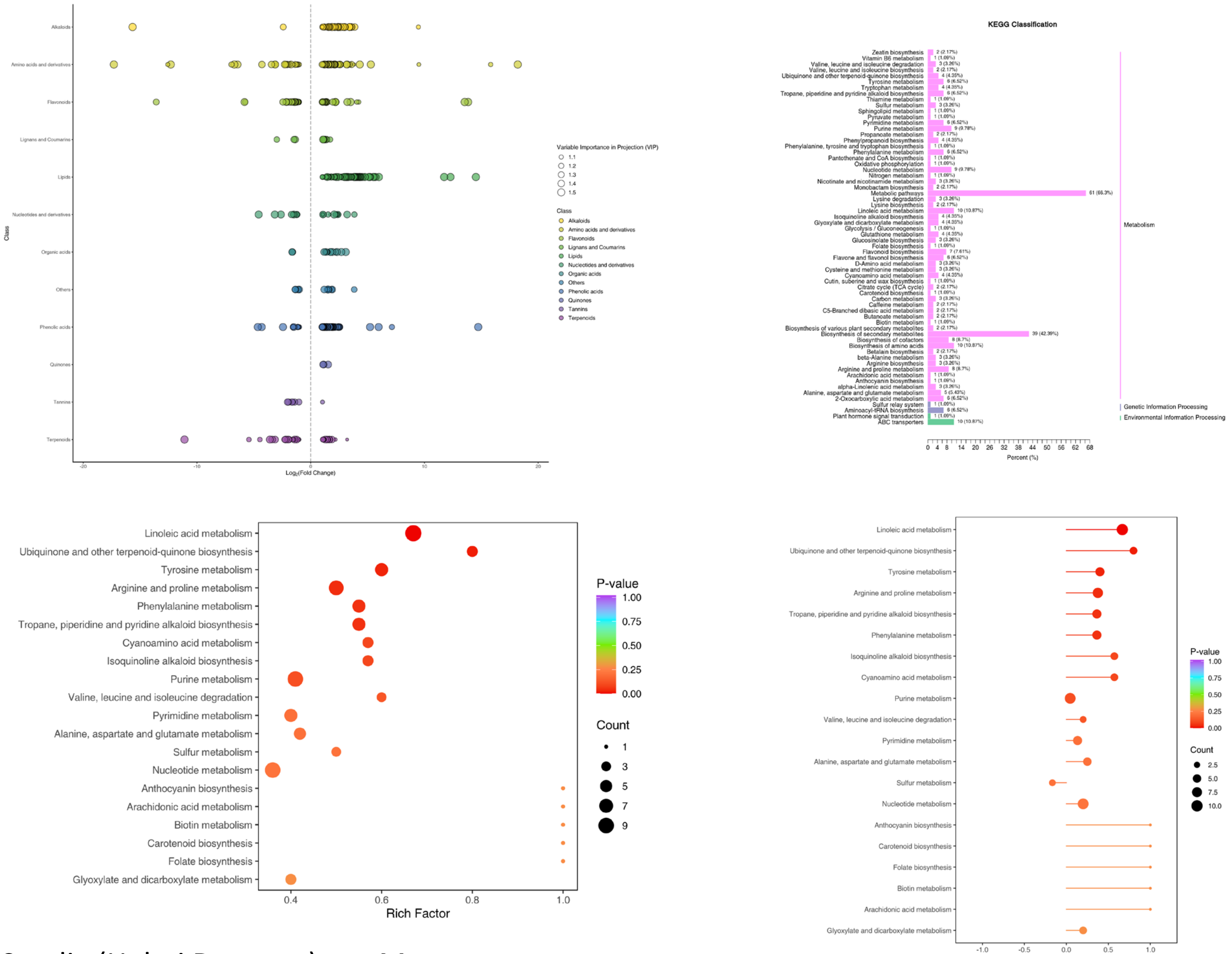


Sandia (Hebei Dongzao)_vs_Maya

**Figure S5 | Pairwise differential metabolite analysis between *Ziziphus jujuba* cultivars. Scatter plot of differential metabolites shown on top, left**. Each dot in the graph indicates a metabolite, and different colors indicate different metabolite subclasses; the horizontal coordinate indicates the logarithmic value of the multiplicative difference in the content of a substance in two groups of samples (log22FC), the larger the absolute value of the horizontal coordinate, the greater the difference in the content of the substance between the two groups of samples, and the size of the dot represents the VIP value. **KEGG classification of differential metabolites on the top right**. The Y-axis shows the name of the KEGG pathway. The number of significant differential metabolites and the proportion of the total significant differential metabolites are shown next to the bar plot. **KEGG enrichment diagram of differential metabolites on bottom left.** The X-axis represents the Rich Factor and the Y-axis represents the pathway. The color of points reflects the p-value. The darker the red, the more significant the enrichment. The size of the dot represents the number of enriched differential metabolites. **Analysis of overall changes in KEGG metabolic pathway on bottom right.** The Y-axis represents the name of differential pathway, and the X-axis represents DA Score. DA Score reflects the overall change of all metabolites in the metabolic pathway. A Score of 1 indicates that the expression trend of all identified metabolites in this pathway is up-regulated, and -1 indicates that the expression trend of all identified metabolites in this pathway is down-regulated. The length of the line represent the absolute value of DA-score while the size of the dot at the end of the line represent the number of differential metabolites. A dot on the left of the line represent the pathway is down-regulated; a dot on the right of the line represents the pathway is up-regulated. The color of the line and dot represent the p-value. The darker the red, the smaller the p-value and the darker the purple, the larger the p-value.

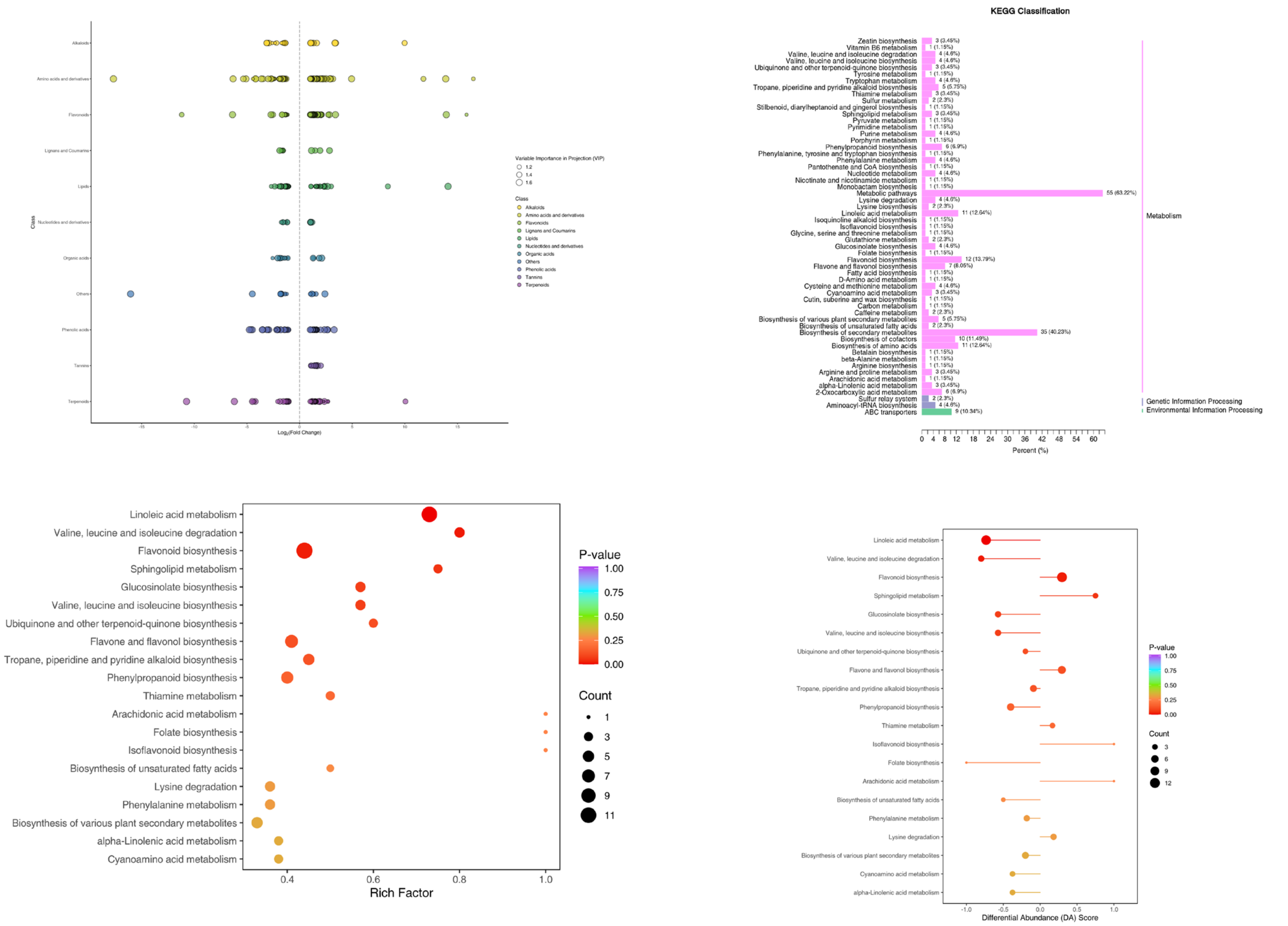


Jinsi_vs_Shanxi Li

**Figure S5 | Pairwise differential metabolite analysis between *Ziziphus jujuba* cultivars. Scatter plot of differential metabolites shown on top, left**. Each dot in the graph indicates a metabolite, and different colors indicate different metabolite subclasses; the horizontal coordinate indicates the logarithmic value of the multiplicative difference in the content of a substance in two groups of samples (log22FC), the larger the absolute value of the horizontal coordinate, the greater the difference in the content of the substance between the two groups of samples, and the size of the dot represents the VIP value. **KEGG classification of differential metabolites on the top right**. The Y-axis shows the name of the KEGG pathway. The number of significant differential metabolites and the proportion of the total significant differential metabolites are shown next to the bar plot. **KEGG enrichment diagram of differential metabolites on bottom left.** The X-axis represents the Rich Factor and the Y-axis represents the pathway. The color of points reflects the p-value. The darker the red, the more significant the enrichment. The size of the dot represents the number of enriched differential metabolites. **Analysis of overall changes in KEGG metabolic pathway on bottom right.** The Y-axis represents the name of differential pathway, and the X-axis represents DA Score. DA Score reflects the overall change of all metabolites in the metabolic pathway. A Score of 1 indicates that the expression trend of all identified metabolites in this pathway is up-regulated, and -1 indicates that the expression trend of all identified metabolites in this pathway is down-regulated. The length of the line represent the absolute value of DA-score while the size of the dot at the end of the line represent the number of differential metabolites. A dot on the left of the line represent the pathway is down-regulated; a dot on the right of the line represents the pathway is up-regulated. The color of the line and dot represent the p-value. The darker the red, the smaller the p-value and the darker the purple, the larger the p-value.

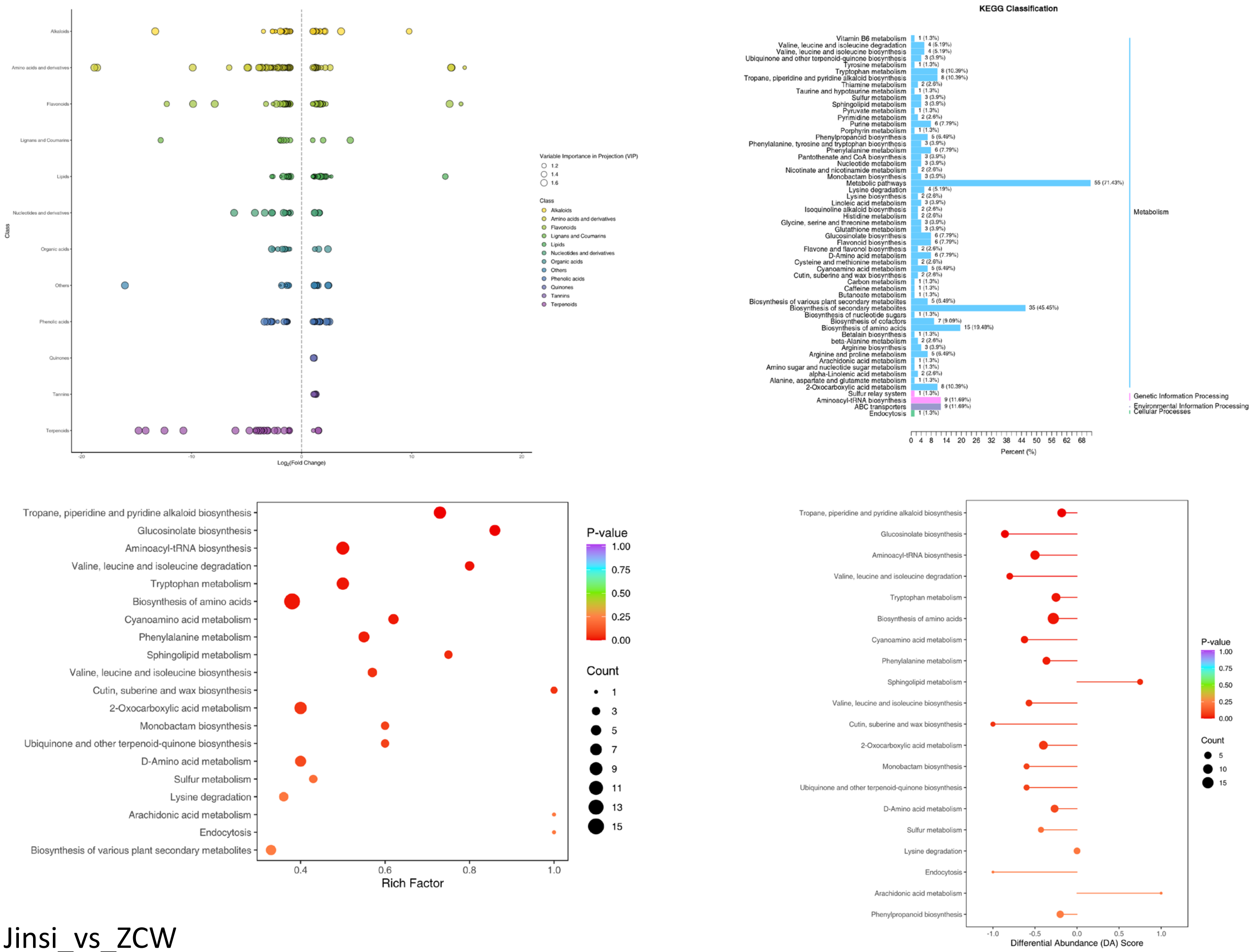


Jinsi_vs_ZCW

**Figure S5 | Pairwise differential metabolite analysis between *Ziziphus jujuba* cultivars. Scatter plot of differential metabolites shown on top, left**. Each dot in the graph indicates a metabolite, and different colors indicate different metabolite subclasses; the horizontal coordinate indicates the logarithmic value of the multiplicative difference in the content of a substance in two groups of samples (log22FC), the larger the absolute value of the horizontal coordinate, the greater the difference in the content of the substance between the two groups of samples, and the size of the dot represents the VIP value. **KEGG classification of differential metabolites on the top right**. The Y-axis shows the name of the KEGG pathway. The number of significant differential metabolites and the proportion of the total significant differential metabolites are shown next to the bar plot. **KEGG enrichment diagram of differential metabolites on bottom left.** The X-axis represents the Rich Factor and the Y-axis represents the pathway. The color of points reflects the p-value. The darker the red, the more significant the enrichment. The size of the dot represents the number of enriched differential metabolites. **Analysis of overall changes in KEGG metabolic pathway on bottom right.** The Y-axis represents the name of differential pathway, and the X-axis represents DA Score. DA Score reflects the overall change of all metabolites in the metabolic pathway. A Score of 1 indicates that the expression trend of all identified metabolites in this pathway is up-regulated, and -1 indicates that the expression trend of all identified metabolites in this pathway is down-regulated. The length of the line represent the absolute value of DA-score while the size of the dot at the end of the line represent the number of differential metabolites. A dot on the left of the line represent the pathway is down-regulated; a dot on the right of the line represents the pathway is up-regulated. The color of the line and dot represent the p-value. The darker the red, the smaller the p-value and the darker the purple, the larger the p-value.

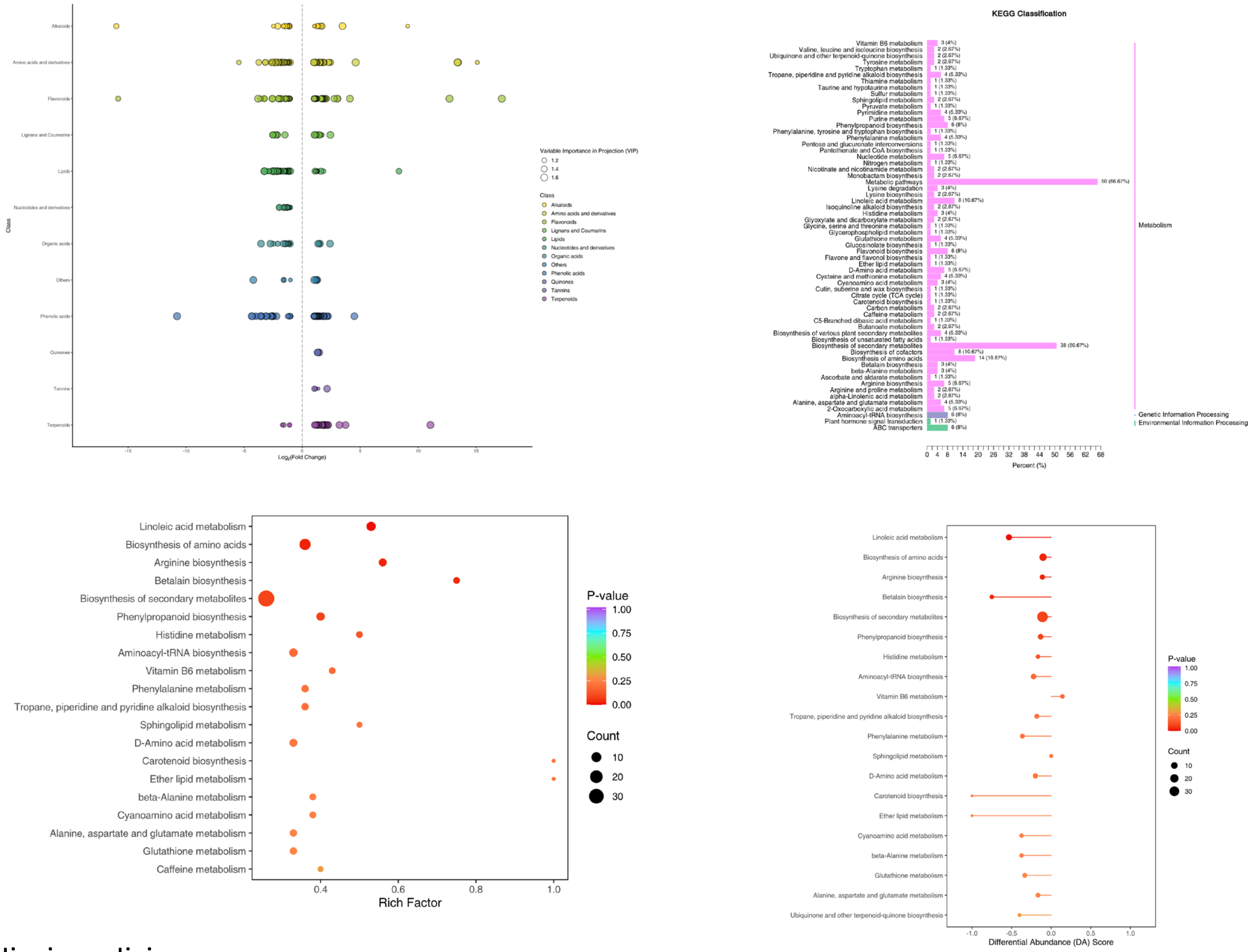


Jinsi_vs_Jixin

**Figure S5 | Pairwise differential metabolite analysis between *Ziziphus jujuba* cultivars. Scatter plot of differential metabolites shown on top, left**. Each dot in the graph indicates a metabolite, and different colors indicate different metabolite subclasses; the horizontal coordinate indicates the logarithmic value of the multiplicative difference in the content of a substance in two groups of samples (log22FC), the larger the absolute value of the horizontal coordinate, the greater the difference in the content of the substance between the two groups of samples, and the size of the dot represents the VIP value. **KEGG classification of differential metabolites on the top right**. The Y-axis shows the name of the KEGG pathway. The number of significant differential metabolites and the proportion of the total significant differential metabolites are shown next to the bar plot. **KEGG enrichment diagram of differential metabolites on bottom left.** The X-axis represents the Rich Factor and the Y-axis represents the pathway. The color of points reflects the p-value. The darker the red, the more significant the enrichment. The size of the dot represents the number of enriched differential metabolites. **Analysis of overall changes in KEGG metabolic pathway on bottom right.** The Y-axis represents the name of differential pathway, and the X-axis represents DA Score. DA Score reflects the overall change of all metabolites in the metabolic pathway. A Score of 1 indicates that the expression trend of all identified metabolites in this pathway is up-regulated, and -1 indicates that the expression trend of all identified metabolites in this pathway is down-regulated. The length of the line represent the absolute value of DA-score while the size of the dot at the end of the line represent the number of differential metabolites. A dot on the left of the line represent the pathway is down-regulated; a dot on the right of the line represents the pathway is up-regulated. The color of the line and dot represent the p-value. The darker the red, the smaller the p-value and the darker the purple, the larger the p-value.

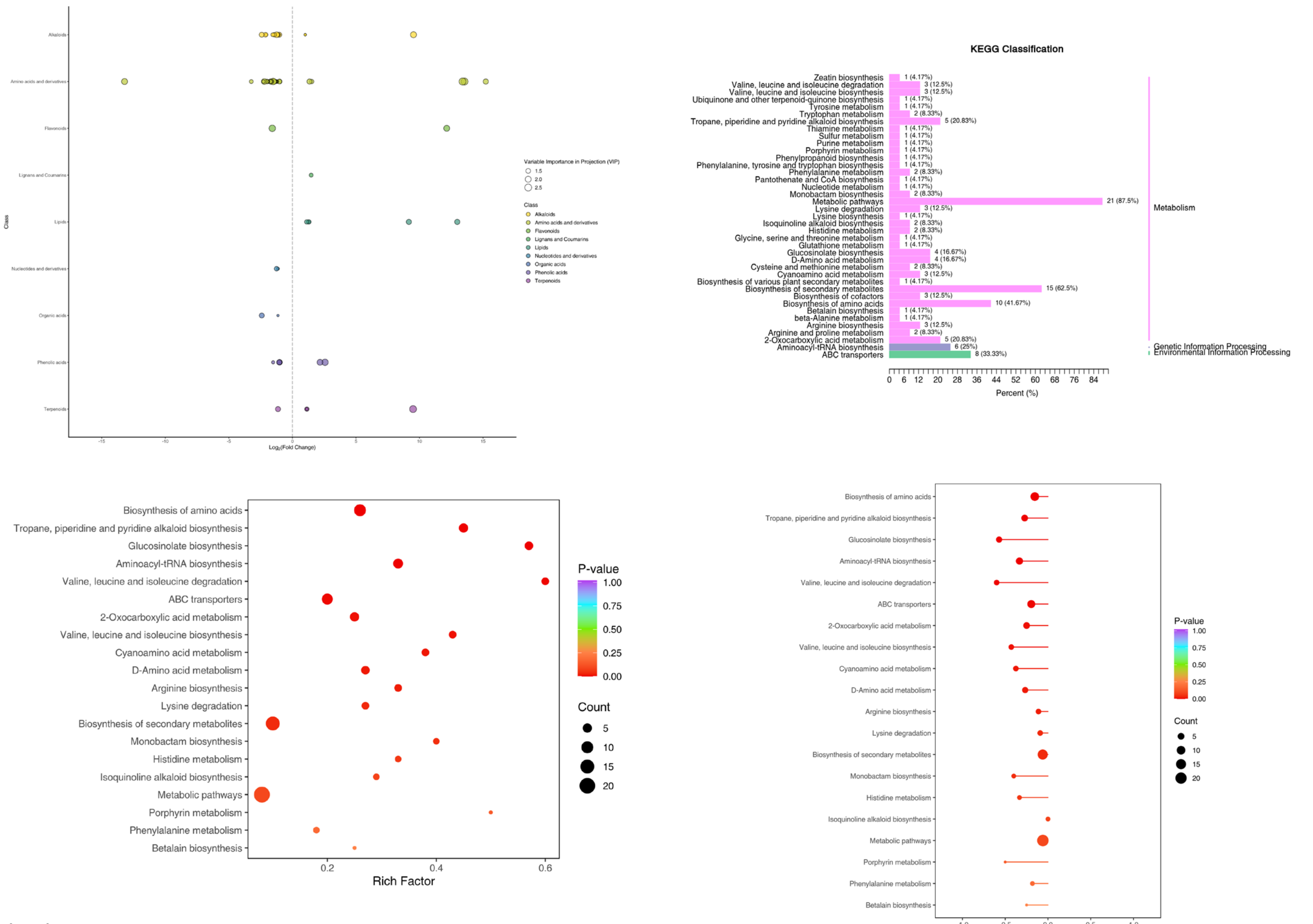


Jinsi_vs_JKW

**Figure S5 | Pairwise differential metabolite analysis between *Ziziphus jujuba* cultivars. Scatter plot of differential metabolites shown on top, left**. Each dot in the graph indicates a metabolite, and different colors indicate different metabolite subclasses; the horizontal coordinate indicates the logarithmic value of the multiplicative difference in the content of a substance in two groups of samples ($\log_2$2FC), the larger the absolute value of the horizontal coordinate, the greater the difference in the content of the substance between the two groups of samples, and the size of the dot represents the VIP value. **KEGG classification of differential metabolites on the top right**. The Y-axis shows the name of the KEGG pathway. The number of significant differential metabolites and the proportion of the total significant differential metabolites are shown next to the bar plot. **KEGG enrichment diagram of differential metabolites on bottom left.** The X-axis represents the Rich Factor and the Y-axis represents the pathway. The color of points reflects the p-value. The darker the red, the more significant the enrichment. The size of the dot represents the number of enriched differential metabolites. **Analysis of overall changes in KEGG metabolic pathway on bottom right.** The Y-axis represents the name of differential pathway, and the X-axis represents DA Score. DA Score reflects the overall change of all metabolites in the metabolic pathway. A Score of 1 indicates that the expression trend of all identified metabolites in this pathway is up-regulated, and -1 indicates that the expression trend of all identified metabolites in this pathway is down-regulated. The length of the line represent the absolute value of DA-score while the size of the dot at the end of the line represent the number of differential metabolites. A dot on the left of the line represent the pathway is down-regulated; a dot on the right of the line represents the pathway is up-regulated. The color of the line and dot represent the p-value. The darker the red, the smaller the p-value and the darker the purple, the larger the p-value.

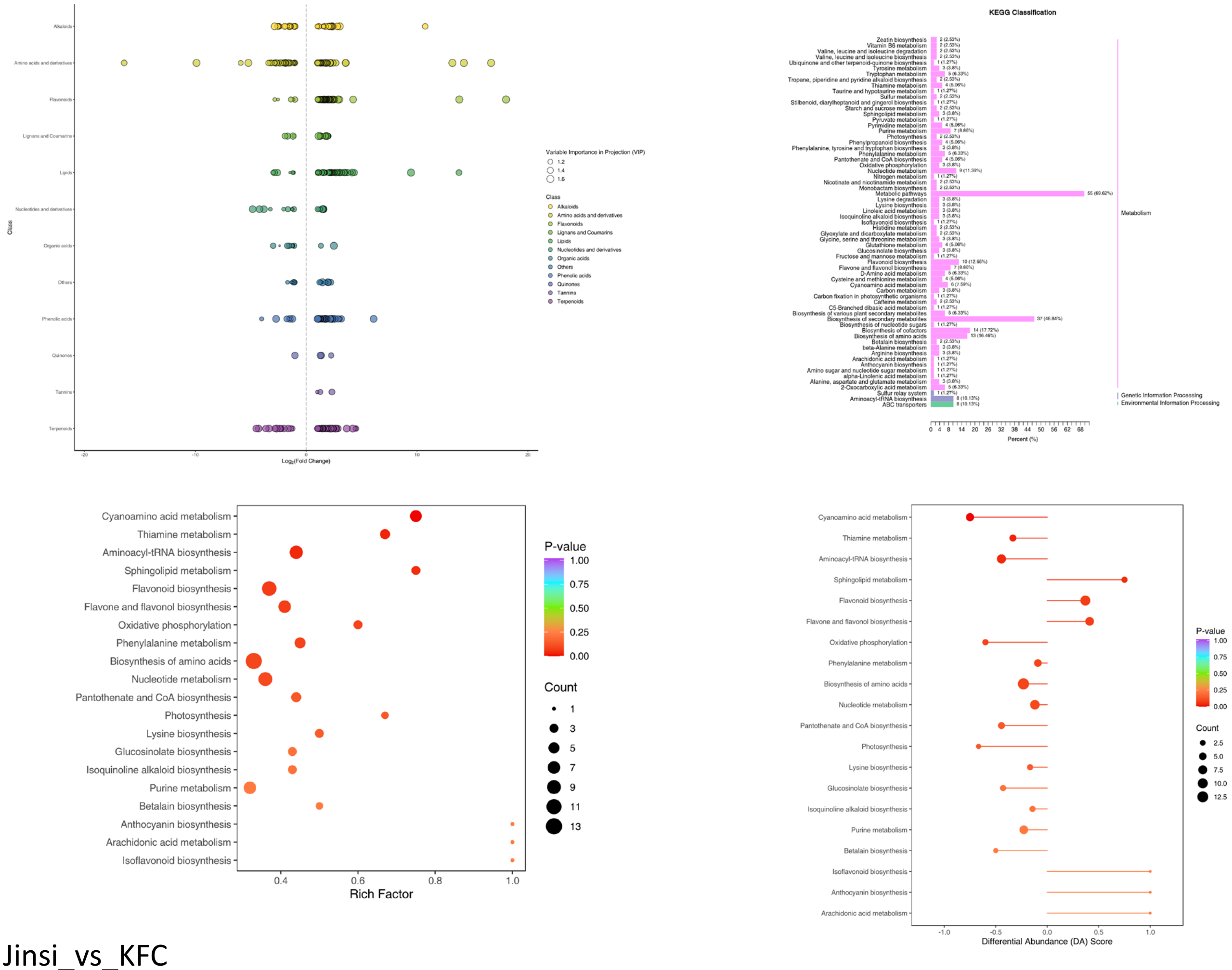


Jinsi_vs_KFC

**Figure S5 | Pairwise differential metabolite analysis between *Ziziphus jujuba* cultivars. Scatter plot of differential metabolites shown on top, left**. Each dot in the graph indicates a metabolite, and different colors indicate different metabolite subclasses; the horizontal coordinate indicates the logarithmic value of the multiplicative difference in the content of a substance in two groups of samples ($log_2 2FC$), the larger the absolute value of the horizontal coordinate, the greater the difference in the content of the substance between the two groups of samples, and the size of the dot represents the VIP value. **KEGG classification of differential metabolites on the top right**. The Y-axis shows the name of the KEGG pathway. The number of significant differential metabolites and the proportion of the total significant differential metabolites are shown next to the bar plot. **KEGG enrichment diagram of differential metabolites on bottom left.** The X-axis represents the Rich Factor and the Y-axis represents the pathway. The color of points reflects the p-value. The darker the red, the more significant the enrichment. The size of the dot represents the number of enriched differential metabolites. **Analysis of overall changes in KEGG metabolic pathway on bottom right.** The Y-axis represents the name of differential pathway, and the X-axis represents DA Score. DA Score reflects the overall change of all metabolites in the metabolic pathway. A Score of 1 indicates that the expression trend of all identified metabolites in this pathway is up-regulated, and -1 indicates that the expression trend of all identified metabolites in this pathway is down-regulated. The length of the line represent the absolute value of DA-score while the size of the dot at the end of the line represent the number of differential metabolites. A dot on the left of the line represent the pathway is down-regulated; a dot on the right of the line represents the pathway is up-regulated. The color of the line and dot represent the p-value. The darker the red, the smaller the p-value and the darker the purple, the larger the p-value.

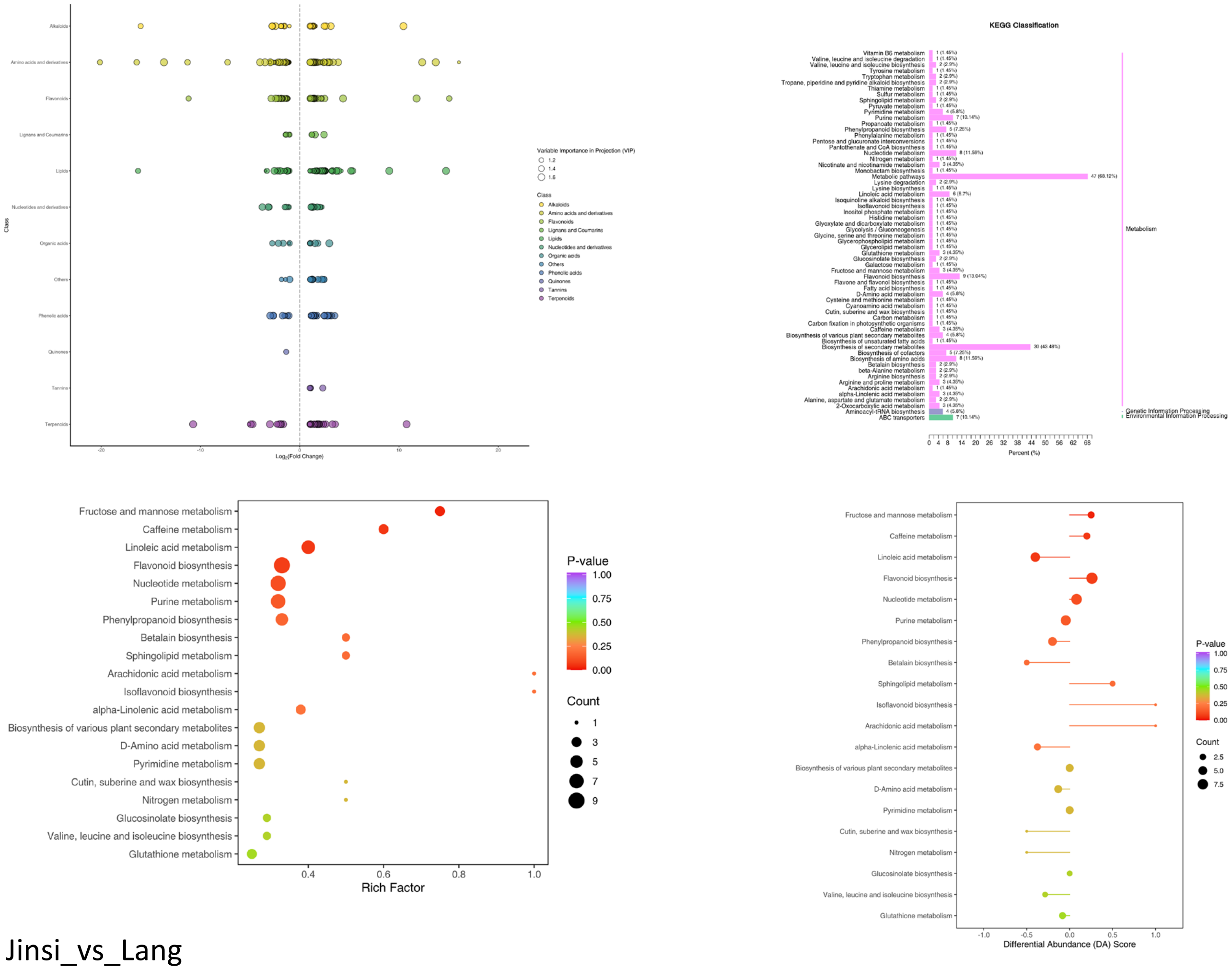


**Figure S5 | Pairwise differential metabolite analysis between *Ziziphus jujuba* cultivars. Scatter plot of differential metabolites shown on top, left**. Each dot in the graph indicates a metabolite, and different colors indicate different metabolite subclasses; the horizontal coordinate indicates the logarithmic value of the multiplicative difference in the content of a substance in two groups of samples (log22FC), the larger the absolute value of the horizontal coordinate, the greater the difference in the content of the substance between the two groups of samples, and the size of the dot represents the VIP value. **KEGG classification of differential metabolites on the top right**. The Y-axis shows the name of the KEGG pathway. The number of significant differential metabolites and the proportion of the total significant differential metabolites are shown next to the bar plot. **KEGG enrichment diagram of differential metabolites on bottom left.** The X-axis represents the Rich Factor and the Y-axis represents the pathway. The color of points reflects the p-value. The darker the red, the more significant the enrichment. The size of the dot represents the number of enriched differential metabolites. **Analysis of overall changes in KEGG metabolic pathway on bottom right.** The Y-axis represents the name of differential pathway, and the X-axis represents DA Score. DA Score reflects the overall change of all metabolites in the metabolic pathway. A Score of 1 indicates that the expression trend of all identified metabolites in this pathway is up-regulated, and -1 indicates that the expression trend of all identified metabolites in this pathway is down-regulated. The length of the line represent the absolute value of DA-score while the size of the dot at the end of the line represent the number of differential metabolites. A dot on the left of the line represent the pathway is down-regulated; a dot on the right of the line represents the pathway is up-regulated. The color of the line and dot represent the p-value. The darker the red, the smaller the p-value and the darker the purple, the larger the p-value.

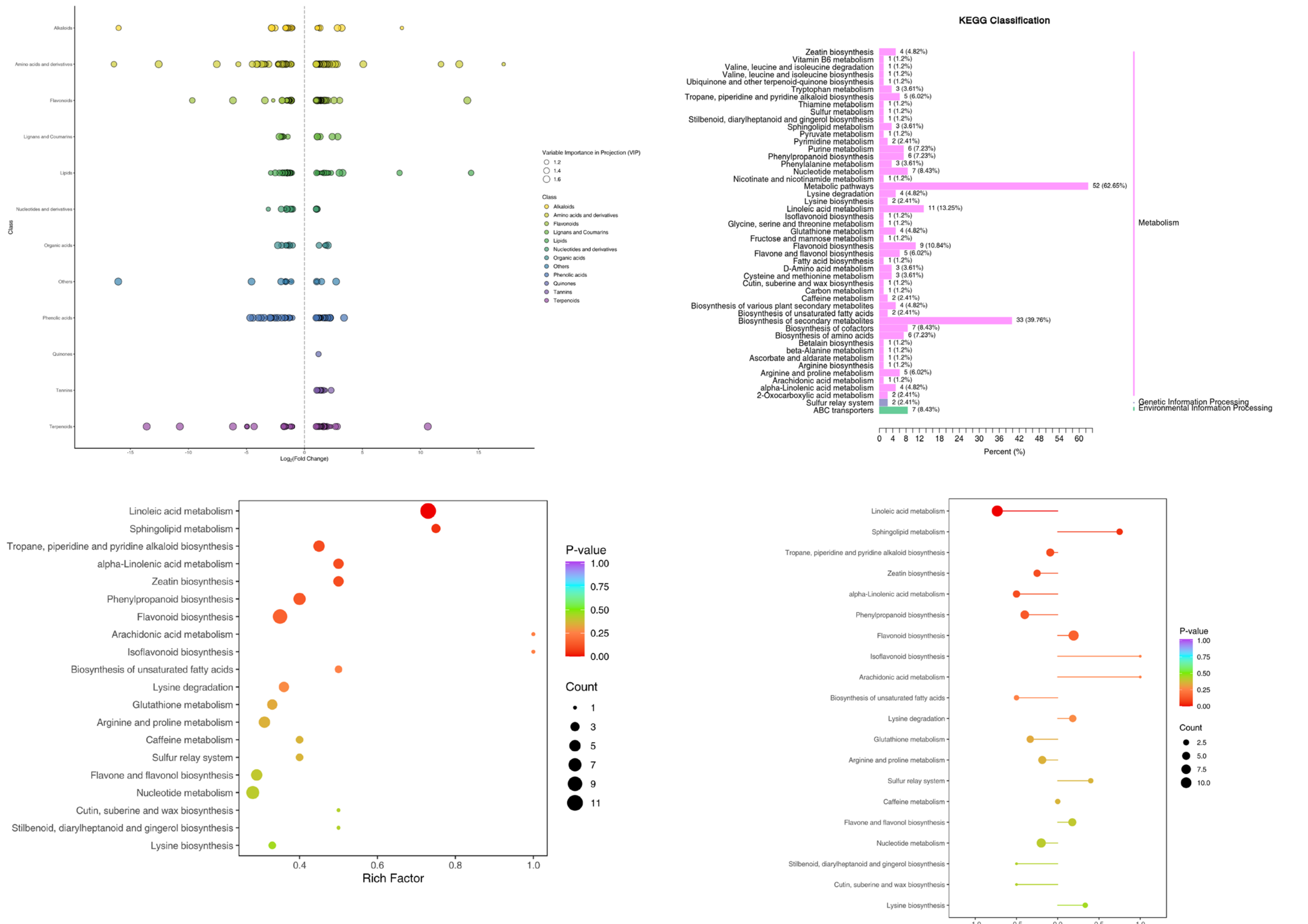


Jinsi_vs_Li

**Figure S5 | Pairwise differential metabolite analysis between *Ziziphus jujuba* cultivars. Scatter plot of differential metabolites shown on top, left**. Each dot in the graph indicates a metabolite, and different colors indicate different metabolite subclasses; the horizontal coordinate indicates the logarithmic value of the multiplicative difference in the content of a substance in two groups of samples (log22FC), the larger the absolute value of the horizontal coordinate, the greater the difference in the content of the substance between the two groups of samples, and the size of the dot represents the VIP value. **KEGG classification of differential metabolites on the top right**. The Y-axis shows the name of the KEGG pathway. The number of significant differential metabolites and the proportion of the total significant differential metabolites are shown next to the bar plot. **KEGG enrichment diagram of differential metabolites on bottom left.** The X-axis represents the Rich Factor and the Y-axis represents the pathway. The color of points reflects the p-value. The darker the red, the more significant the enrichment. The size of the dot represents the number of enriched differential metabolites. **Analysis of overall changes in KEGG metabolic pathway on bottom right.** The Y-axis represents the name of differential pathway, and the X-axis represents DA Score. DA Score reflects the overall change of all metabolites in the metabolic pathway. A Score of 1 indicates that the expression trend of all identified metabolites in this pathway is up-regulated, and -1 indicates that the expression trend of all identified metabolites in this pathway is down-regulated. The length of the line represent the absolute value of DA-score while the size of the dot at the end of the line represent the number of differential metabolites. A dot on the left of the line represent the pathway is down-regulated; a dot on the right of the line represents the pathway is up-regulated. The color of the line and dot represent the p-value. The darker the red, the smaller the p-value and the darker the purple, the larger the p-value.

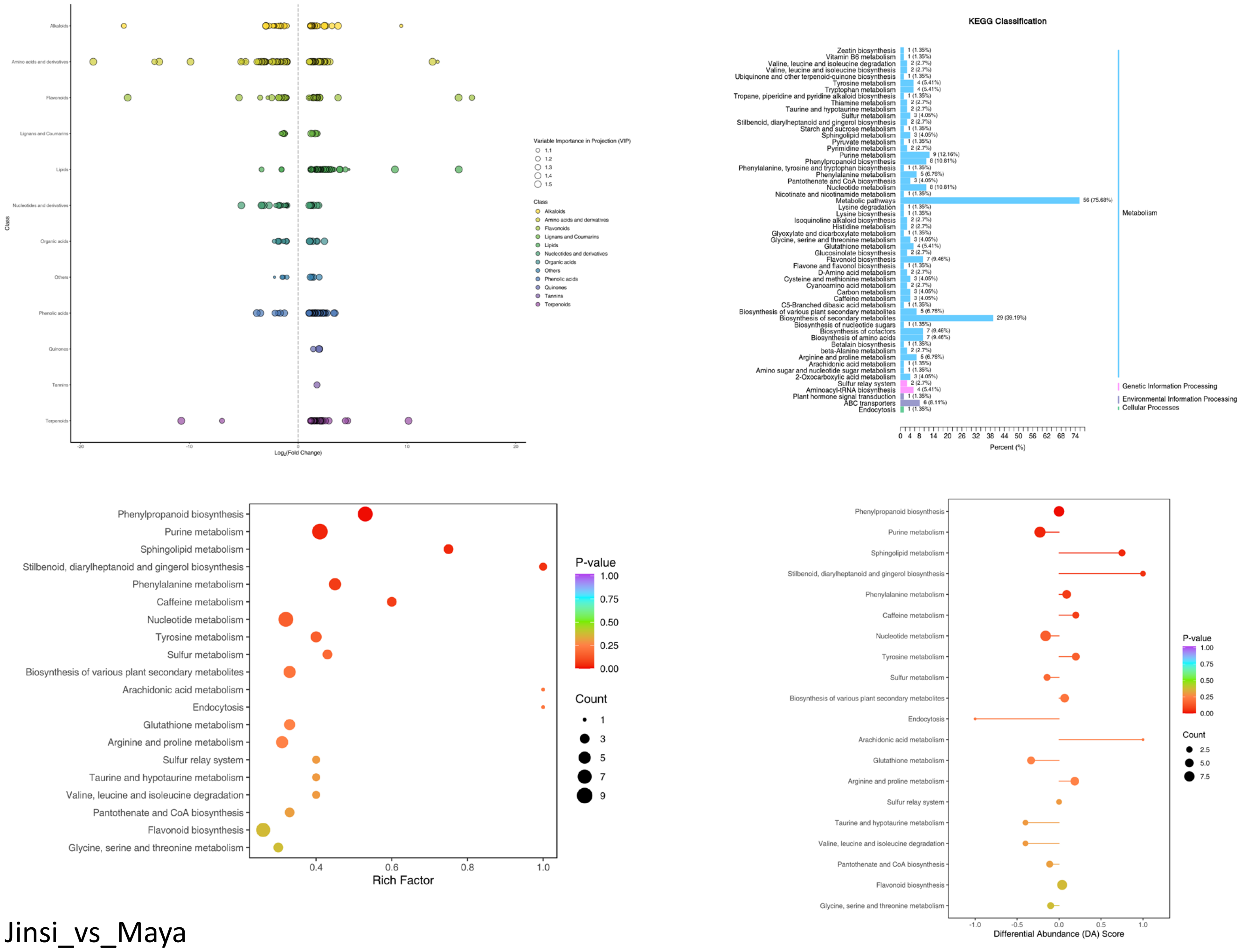


**Figure S5 | Pairwise differential metabolite analysis between *Ziziphus jujuba* cultivars. Scatter plot of differential metabolites shown on top, left**. Each dot in the graph indicates a metabolite, and different colors indicate different metabolite subclasses; the horizontal coordinate indicates the logarithmic value of the multiplicative difference in the content of a substance in two groups of samples (log22FC), the larger the absolute value of the horizontal coordinate, the greater the difference in the content of the substance between the two groups of samples, and the size of the dot represents the VIP value. **KEGG classification of differential metabolites on the top right**. The Y-axis shows the name of the KEGG pathway. The number of significant differential metabolites and the proportion of the total significant differential metabolites are shown next to the bar plot. **KEGG enrichment diagram of differential metabolites on bottom left.** The X-axis represents the Rich Factor and the Y-axis represents the pathway. The color of points reflects the p-value. The darker the red, the more significant the enrichment. The size of the dot represents the number of enriched differential metabolites. **Analysis of overall changes in KEGG metabolic pathway on bottom right.** The Y-axis represents the name of differential pathway, and the X-axis represents DA Score. DA Score reflects the overall change of all metabolites in the metabolic pathway. A Score of 1 indicates that the expression trend of all identified metabolites in this pathway is up-regulated, and -1 indicates that the expression trend of all identified metabolites in this pathway is down-regulated. The length of the line represent the absolute value of DA-score while the size of the dot at the end of the line represent the number of differential metabolites. A dot on the left of the line represent the pathway is down-regulated; a dot on the right of the line represents the pathway is up-regulated. The color of the line and dot represent the p-value. The darker the red, the smaller the p-value and the darker the purple, the larger the p-value.

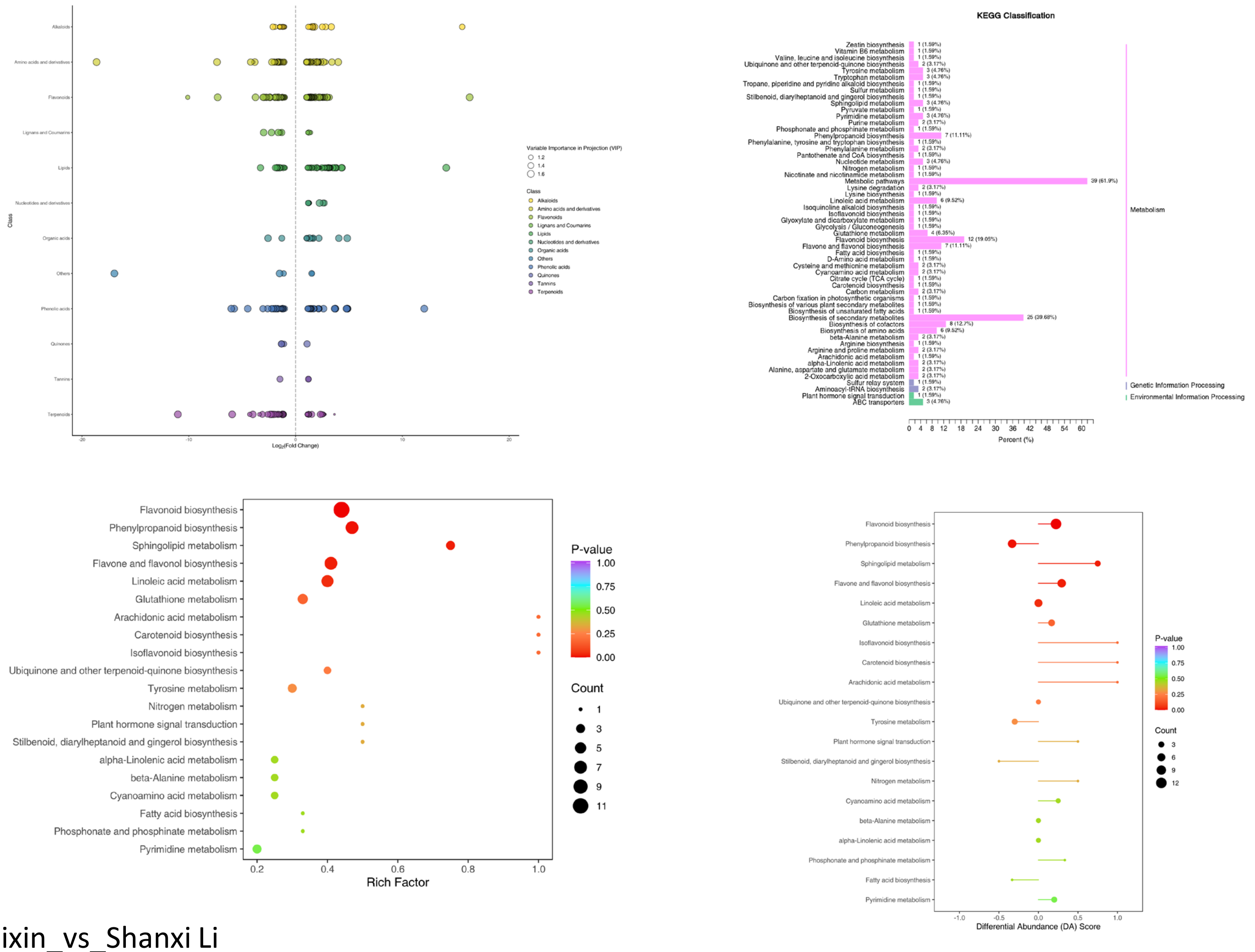


Jixin_vs_Shanxi Li

**Figure S5 | Pairwise differential metabolite analysis between *Ziziphus jujuba* cultivars. Scatter plot of differential metabolites shown on top, left**. Each dot in the graph indicates a metabolite, and different colors indicate different metabolite subclasses; the horizontal coordinate indicates the logarithmic value of the multiplicative difference in the content of a substance in two groups of samples (log22FC), the larger the absolute value of the horizontal coordinate, the greater the difference in the content of the substance between the two groups of samples, and the size of the dot represents the VIP value. **KEGG classification of differential metabolites on the top right**. The Y-axis shows the name of the KEGG pathway. The number of significant differential metabolites and the proportion of the total significant differential metabolites are shown next to the bar plot. **KEGG enrichment diagram of differential metabolites on bottom left.** The X-axis represents the Rich Factor and the Y-axis represents the pathway. The color of points reflects the p-value. The darker the red, the more significant the enrichment. The size of the dot represents the number of enriched differential metabolites. **Analysis of overall changes in KEGG metabolic pathway on bottom right.** The Y-axis represents the name of differential pathway, and the X-axis represents DA Score. DA Score reflects the overall change of all metabolites in the metabolic pathway. A Score of 1 indicates that the expression trend of all identified metabolites in this pathway is up-regulated, and -1 indicates that the expression trend of all identified metabolites in this pathway is down-regulated. The length of the line represent the absolute value of DA-score while the size of the dot at the end of the line represent the number of differential metabolites. A dot on the left of the line represent the pathway is down-regulated; a dot on the right of the line represents the pathway is up-regulated. The color of the line and dot represent the p-value. The darker the red, the smaller the p-value and the darker the purple, the larger the p-value.

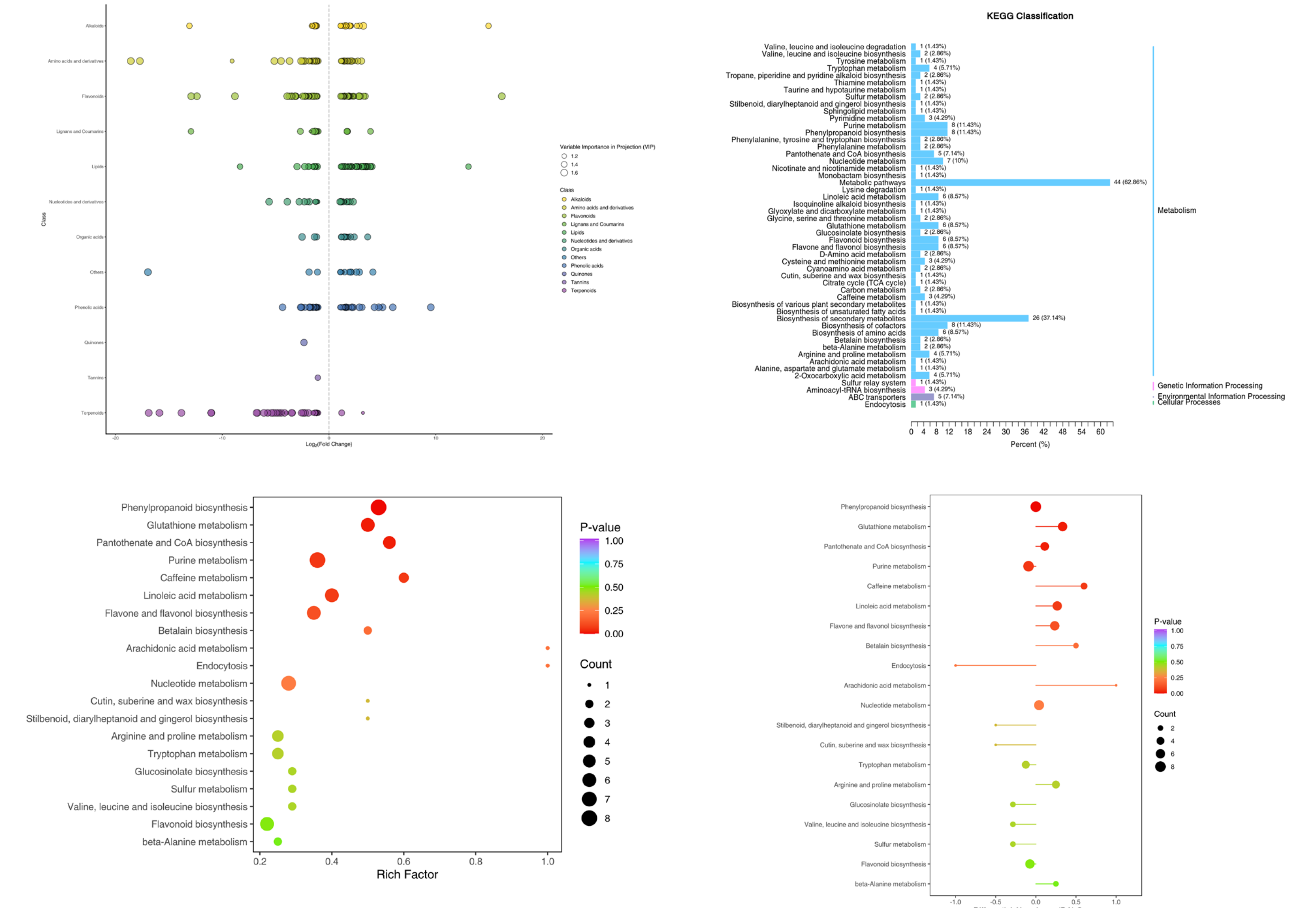


Jixin_vs_ZCW

**Figure S5 | Pairwise differential metabolite analysis between *Ziziphus jujuba* cultivars. Scatter plot of differential metabolites shown on top, left**. Each dot in the graph indicates a metabolite, and different colors indicate different metabolite subclasses; the horizontal coordinate indicates the logarithmic value of the multiplicative difference in the content of a substance in two groups of samples (log22FC), the larger the absolute value of the horizontal coordinate, the greater the difference in the content of the substance between the two groups of samples, and the size of the dot represents the VIP value. **KEGG classification of differential metabolites on the top right**. The Y-axis shows the name of the KEGG pathway. The number of significant differential metabolites and the proportion of the total significant differential metabolites are shown next to the bar plot. **KEGG enrichment diagram of differential metabolites on bottom left.** The X-axis represents the Rich Factor and the Y-axis represents the pathway. The color of points reflects the p-value. The darker the red, the more significant the enrichment. The size of the dot represents the number of enriched differential metabolites. **Analysis of overall changes in KEGG metabolic pathway on bottom right.** The Y-axis represents the name of differential pathway, and the X-axis represents DA Score. DA Score reflects the overall change of all metabolites in the metabolic pathway. A Score of 1 indicates that the expression trend of all identified metabolites in this pathway is up-regulated, and -1 indicates that the expression trend of all identified metabolites in this pathway is down-regulated. The length of the line represent the absolute value of DA-score while the size of the dot at the end of the line represent the number of differential metabolites. A dot on the left of the line represent the pathway is down-regulated; a dot on the right of the line represents the pathway is up-regulated. The color of the line and dot represent the p-value. The darker the red, the smaller the p-value and the darker the purple, the larger the p-value.

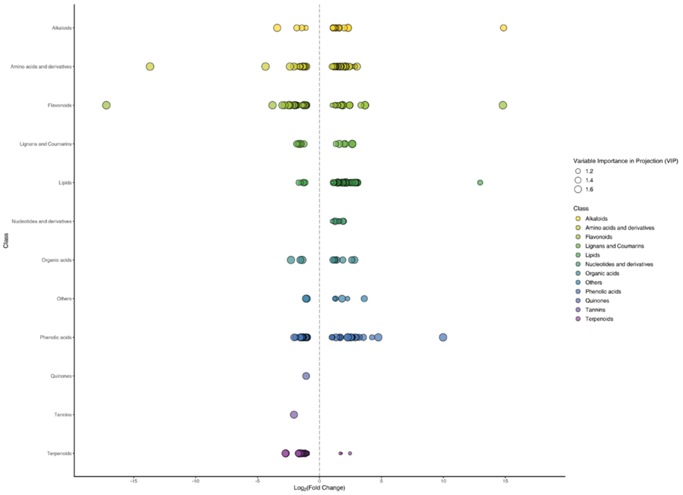


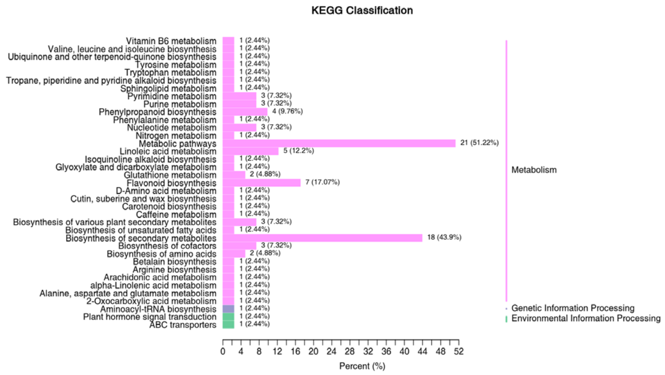


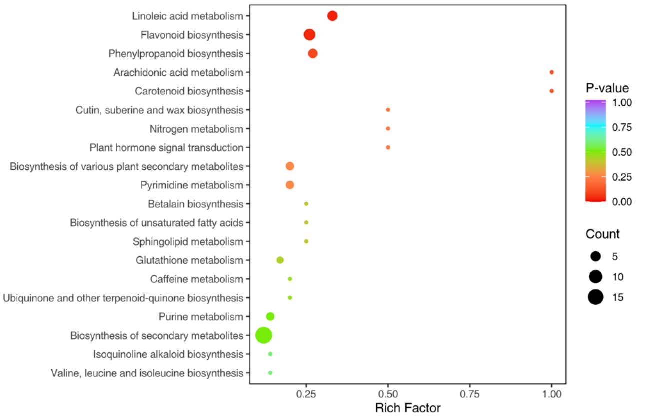


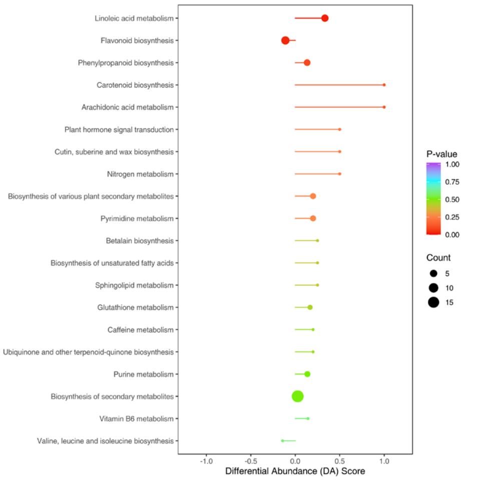


Jixin_vs_JKW

**Figure S5 | Pairwise differential metabolite analysis between *Ziziphus jujuba* cultivars. Scatter plot of differential metabolites shown on top, left**. Each dot in the graph indicates a metabolite, and different colors indicate different metabolite subclasses; the horizontal coordinate indicates the logarithmic value of the multiplicative difference in the content of a substance in two groups of samples (log22FC), the larger the absolute value of the horizontal coordinate, the greater the difference in the content of the substance between the two groups of samples, and the size of the dot represents the VIP value. **KEGG classification of differential metabolites on the top right**. The Y-axis shows the name of the KEGG pathway. The number of significant differential metabolites and the proportion of the total significant differential metabolites are shown next to the bar plot. **KEGG enrichment diagram of differential metabolites on bottom left.** The X-axis represents the Rich Factor and the Y-axis represents the pathway. The color of points reflects the p-value. The darker the red, the more significant the enrichment. The size of the dot represents the number of enriched differential metabolites. **Analysis of overall changes in KEGG metabolic pathway on bottom right.** The Y-axis represents the name of differential pathway, and the X-axis represents DA Score. DA Score reflects the overall change of all metabolites in the metabolic pathway. A Score of 1 indicates that the expression trend of all identified metabolites in this pathway is up-regulated, and -1 indicates that the expression trend of all identified metabolites in this pathway is down-regulated. The length of the line represent the absolute value of DA-score while the size of the dot at the end of the line represent the number of differential metabolites. A dot on the left of the line represent the pathway is down-regulated; a dot on the right of the line represents the pathway is up-regulated. The color of the line and dot represent the p-value. The darker the red, the smaller the p-value and the darker the purple, the larger the p-value.

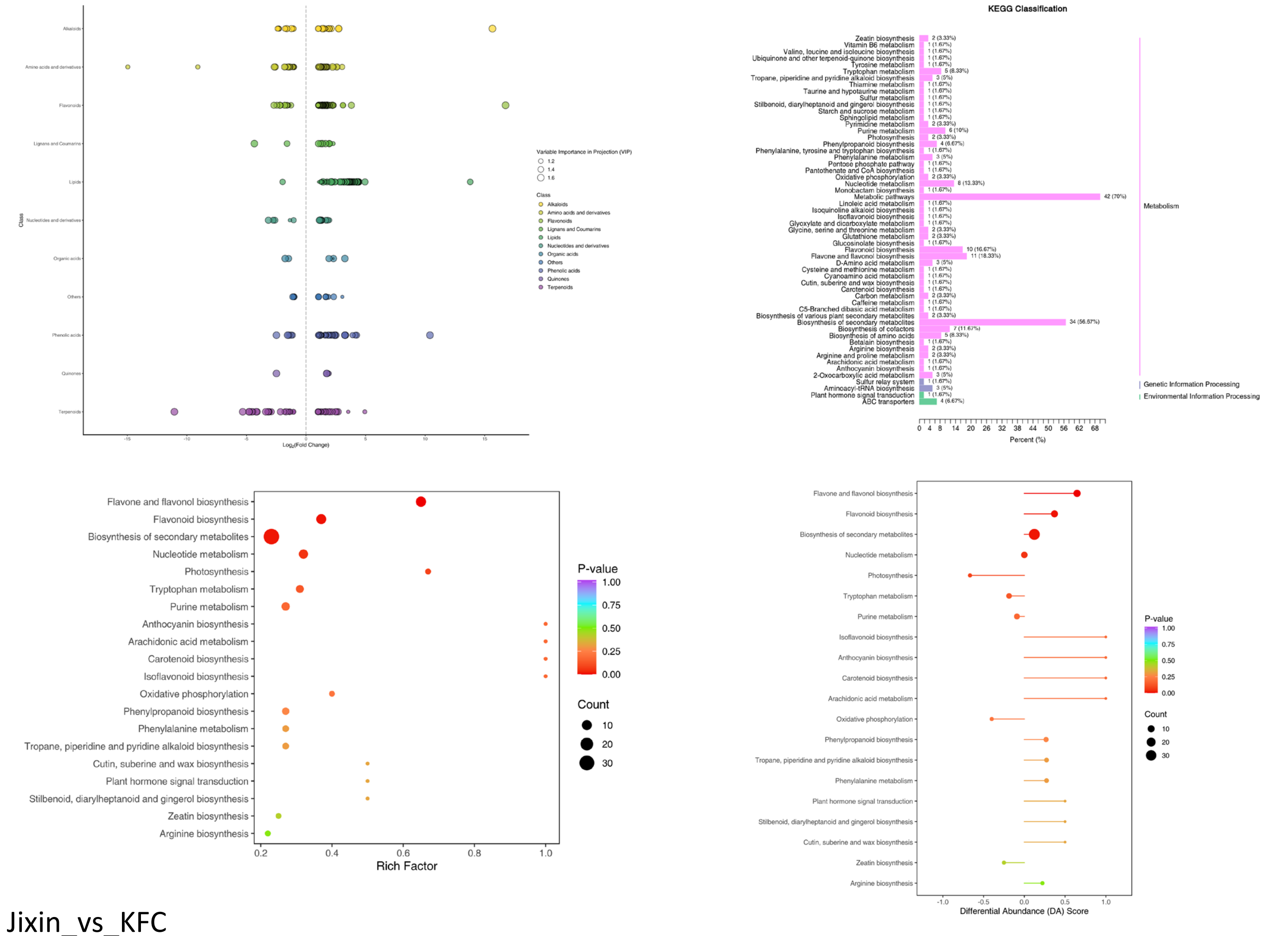


Jixin_vs_KFC

**Figure S5 | Pairwise differential metabolite analysis between *Ziziphus jujuba* cultivars. Scatter plot of differential metabolites shown on top, left**. Each dot in the graph indicates a metabolite, and different colors indicate different metabolite subclasses; the horizontal coordinate indicates the logarithmic value of the multiplicative difference in the content of a substance in two groups of samples (log22FC), the larger the absolute value of the horizontal coordinate, the greater the difference in the content of the substance between the two groups of samples, and the size of the dot represents the VIP value. **KEGG classification of differential metabolites on the top right**. The Y-axis shows the name of the KEGG pathway. The number of significant differential metabolites and the proportion of the total significant differential metabolites are shown next to the bar plot. **KEGG enrichment diagram of differential metabolites on bottom left.** The X-axis represents the Rich Factor and the Y-axis represents the pathway. The color of points reflects the p-value. The darker the red, the more significant the enrichment. The size of the dot represents the number of enriched differential metabolites. **Analysis of overall changes in KEGG metabolic pathway on bottom right.** The Y-axis represents the name of differential pathway, and the X-axis represents DA Score. DA Score reflects the overall change of all metabolites in the metabolic pathway. A Score of 1 indicates that the expression trend of all identified metabolites in this pathway is up-regulated, and -1 indicates that the expression trend of all identified metabolites in this pathway is down-regulated. The length of the line represent the absolute value of DA-score while the size of the dot at the end of the line represent the number of differential metabolites. A dot on the left of the line represent the pathway is down-regulated; a dot on the right of the line represents the pathway is up-regulated. The color of the line and dot represent the p-value. The darker the red, the smaller the p-value and the darker the purple, the larger the p-value.

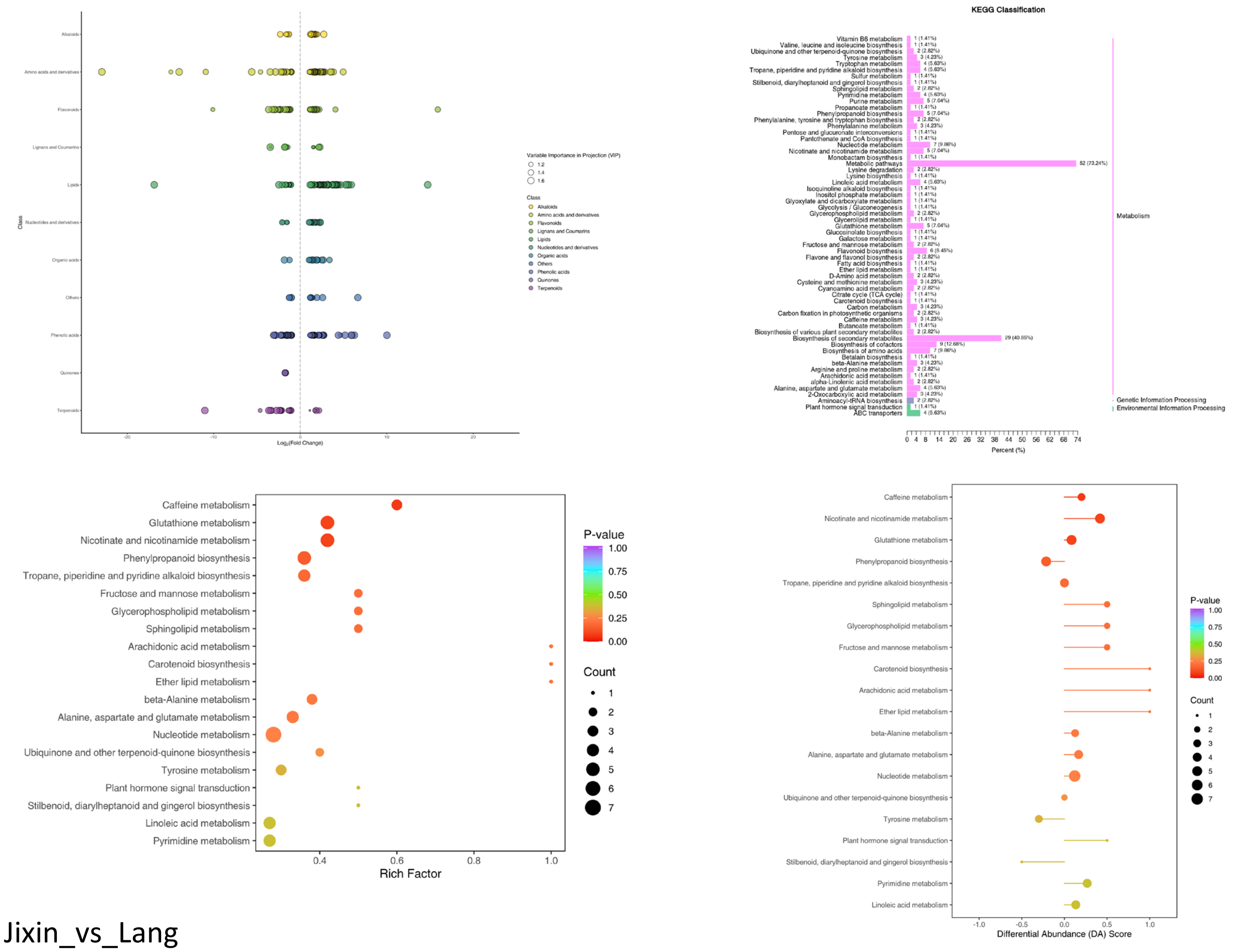


**Figure S5 | Pairwise differential metabolite analysis between *Ziziphus jujuba* cultivars. Scatter plot of differential metabolites shown on top, left**. Each dot in the graph indicates a metabolite, and different colors indicate different metabolite subclasses; the horizontal coordinate indicates the logarithmic value of the multiplicative difference in the content of a substance in two groups of samples (log22FC), the larger the absolute value of the horizontal coordinate, the greater the difference in the content of the substance between the two groups of samples, and the size of the dot represents the VIP value. **KEGG classification of differential metabolites on the top right**. The Y-axis shows the name of the KEGG pathway. The number of significant differential metabolites and the proportion of the total significant differential metabolites are shown next to the bar plot. **KEGG enrichment diagram of differential metabolites on bottom left.** The X-axis represents the Rich Factor and the Y-axis represents the pathway. The color of points reflects the p-value. The darker the red, the more significant the enrichment. The size of the dot represents the number of enriched differential metabolites. **Analysis of overall changes in KEGG metabolic pathway on bottom right.** The Y-axis represents the name of differential pathway, and the X-axis represents DA Score. DA Score reflects the overall change of all metabolites in the metabolic pathway. A Score of 1 indicates that the expression trend of all identified metabolites in this pathway is up-regulated, and -1 indicates that the expression trend of all identified metabolites in this pathway is down-regulated. The length of the line represent the absolute value of DA-score while the size of the dot at the end of the line represent the number of differential metabolites. A dot on the left of the line represent the pathway is down-regulated; a dot on the right of the line represents the pathway is up-regulated. The color of the line and dot represent the p-value. The darker the red, the smaller the p-value and the darker the purple, the larger the p-value.

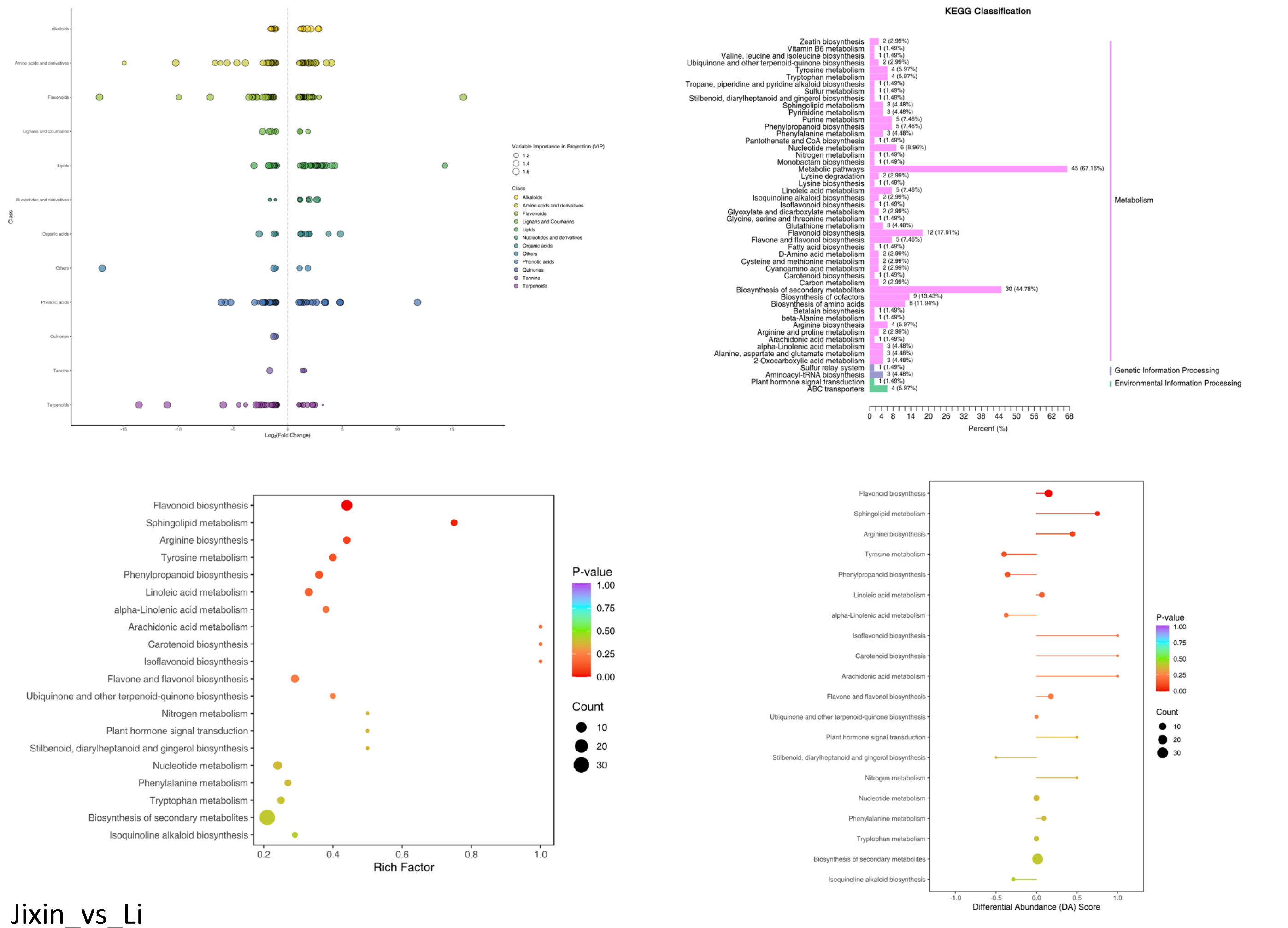


Jixin_vs_Li

**Figure S5 | Pairwise differential metabolite analysis between *Ziziphus jujuba* cultivars. Scatter plot of differential metabolites shown on top, left**. Each dot in the graph indicates a metabolite, and different colors indicate different metabolite subclasses; the horizontal coordinate indicates the logarithmic value of the multiplicative difference in the content of a substance in two groups of samples (log22FC), the larger the absolute value of the horizontal coordinate, the greater the difference in the content of the substance between the two groups of samples, and the size of the dot represents the VIP value. **KEGG classification of differential metabolites on the top right**. The Y-axis shows the name of the KEGG pathway. The number of significant differential metabolites and the proportion of the total significant differential metabolites are shown next to the bar plot. **KEGG enrichment diagram of differential metabolites on bottom left.** The X-axis represents the Rich Factor and the Y-axis represents the pathway. The color of points reflects the p-value. The darker the red, the more significant the enrichment. The size of the dot represents the number of enriched differential metabolites. **Analysis of overall changes in KEGG metabolic pathway on bottom right.** The Y-axis represents the name of differential pathway, and the X-axis represents DA Score. DA Score reflects the overall change of all metabolites in the metabolic pathway. A Score of 1 indicates that the expression trend of all identified metabolites in this pathway is up-regulated, and -1 indicates that the expression trend of all identified metabolites in this pathway is down-regulated. The length of the line represent the absolute value of DA-score while the size of the dot at the end of the line represent the number of differential metabolites. A dot on the left of the line represent the pathway is down-regulated; a dot on the right of the line represents the pathway is up-regulated. The color of the line and dot represent the p-value. The darker the red, the smaller the p-value and the darker the purple, the larger the p-value.

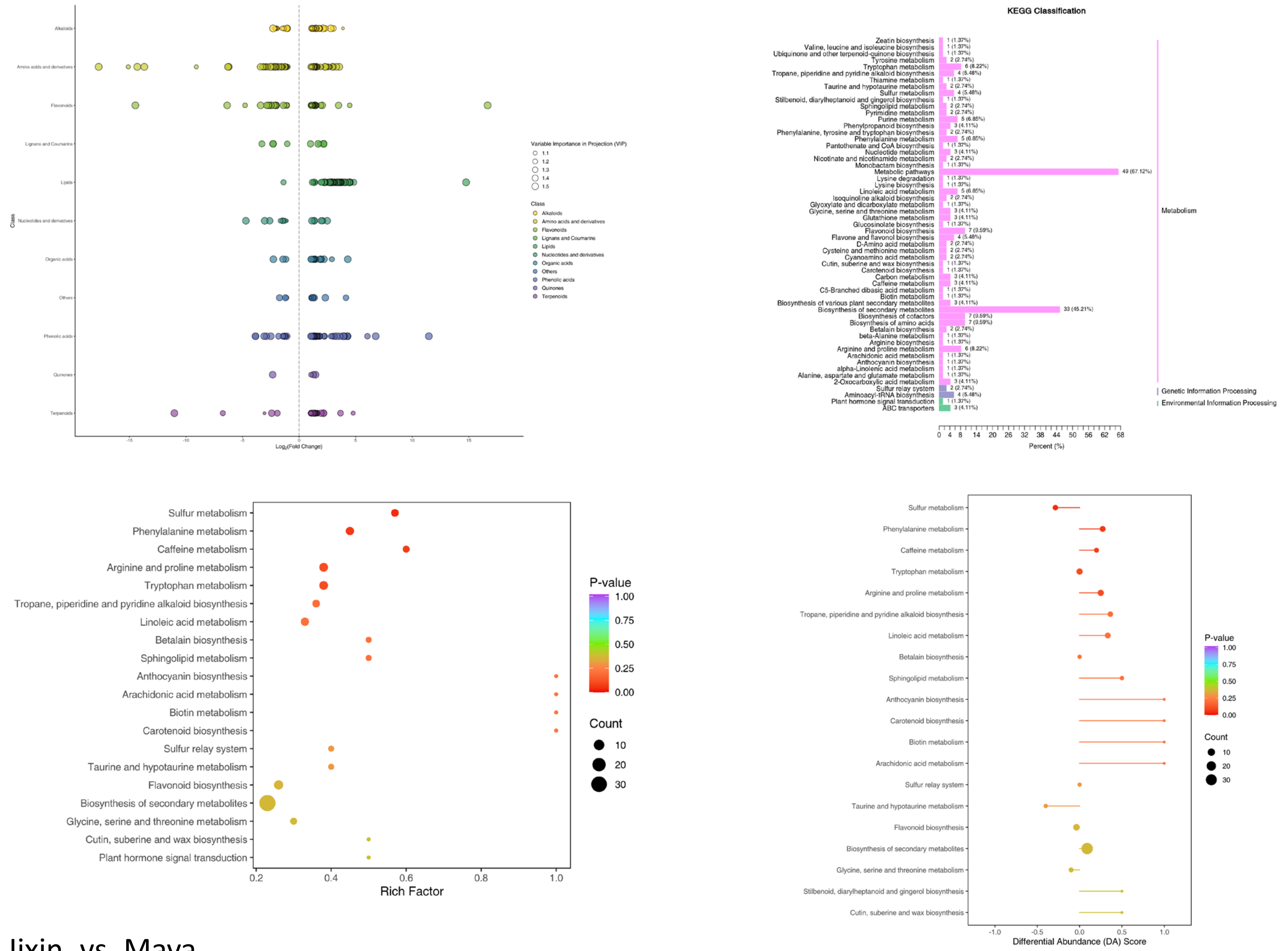


Jixin_vs_Maya

**Figure S5 | Pairwise differential metabolite analysis between *Ziziphus jujuba* cultivars. Scatter plot of differential metabolites shown on top, left**. Each dot in the graph indicates a metabolite, and different colors indicate different metabolite subclasses; the horizontal coordinate indicates the logarithmic value of the multiplicative difference in the content of a substance in two groups of samples (log22FC), the larger the absolute value of the horizontal coordinate, the greater the difference in the content of the substance between the two groups of samples, and the size of the dot represents the VIP value. **KEGG classification of differential metabolites on the top right**. The Y-axis shows the name of the KEGG pathway. The number of significant differential metabolites and the proportion of the total significant differential metabolites are shown next to the bar plot. **KEGG enrichment diagram of differential metabolites on bottom left.** The X-axis represents the Rich Factor and the Y-axis represents the pathway. The color of points reflects the p-value. The darker the red, the more significant the enrichment. The size of the dot represents the number of enriched differential metabolites. **Analysis of overall changes in KEGG metabolic pathway on bottom right.** The Y-axis represents the name of differential pathway, and the X-axis represents DA Score. DA Score reflects the overall change of all metabolites in the metabolic pathway. A Score of 1 indicates that the expression trend of all identified metabolites in this pathway is up-regulated, and -1 indicates that the expression trend of all identified metabolites in this pathway is down-regulated. The length of the line represent the absolute value of DA-score while the size of the dot at the end of the line represent the number of differential metabolites. A dot on the left of the line represent the pathway is down-regulated; a dot on the right of the line represents the pathway is up-regulated. The color of the line and dot represent the p-value. The darker the red, the smaller the p-value and the darker the purple, the larger the p-value.

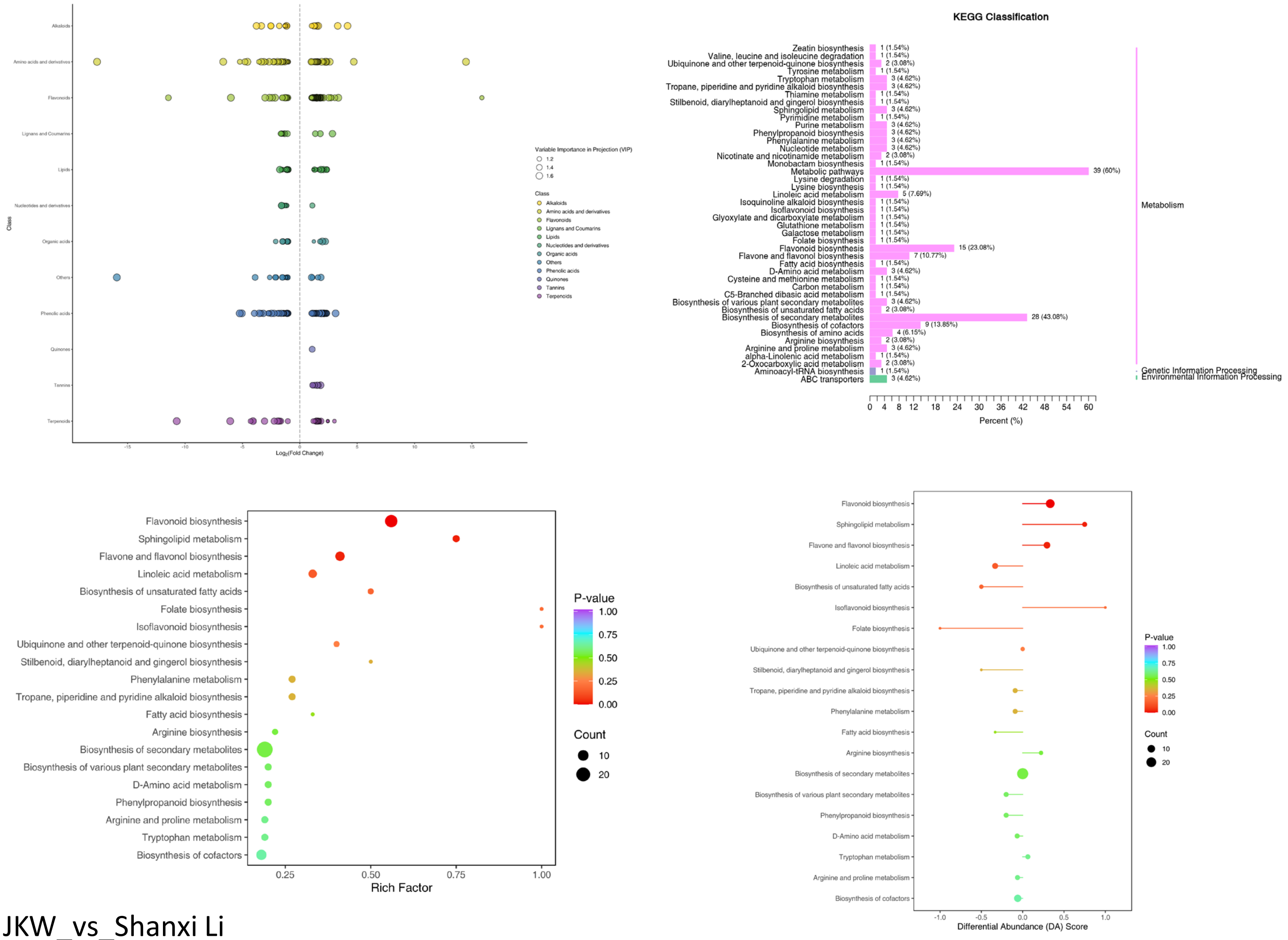


JKW_vs_Shanxi Li

**Figure S5 | Pairwise differential metabolite analysis between *Ziziphus jujuba* cultivars. Scatter plot of differential metabolites shown on top, left**. Each dot in the graph indicates a metabolite, and different colors indicate different metabolite subclasses; the horizontal coordinate indicates the logarithmic value of the multiplicative difference in the content of a substance in two groups of samples (log22FC), the larger the absolute value of the horizontal coordinate, the greater the difference in the content of the substance between the two groups of samples, and the size of the dot represents the VIP value. **KEGG classification of differential metabolites on the top right**. The Y-axis shows the name of the KEGG pathway. The number of significant differential metabolites and the proportion of the total significant differential metabolites are shown next to the bar plot. **KEGG enrichment diagram of differential metabolites on bottom left.** The X-axis represents the Rich Factor and the Y-axis represents the pathway. The color of points reflects the p-value. The darker the red, the more significant the enrichment. The size of the dot represents the number of enriched differential metabolites. **Analysis of overall changes in KEGG metabolic pathway on bottom right.** The Y-axis represents the name of differential pathway, and the X-axis represents DA Score. DA Score reflects the overall change of all metabolites in the metabolic pathway. A Score of 1 indicates that the expression trend of all identified metabolites in this pathway is up-regulated, and -1 indicates that the expression trend of all identified metabolites in this pathway is down-regulated. The length of the line represent the absolute value of DA-score while the size of the dot at the end of the line represent the number of differential metabolites. A dot on the left of the line represent the pathway is down-regulated; a dot on the right of the line represents the pathway is up-regulated. The color of the line and dot represent the p-value. The darker the red, the smaller the p-value and the darker the purple, the larger the p-value.

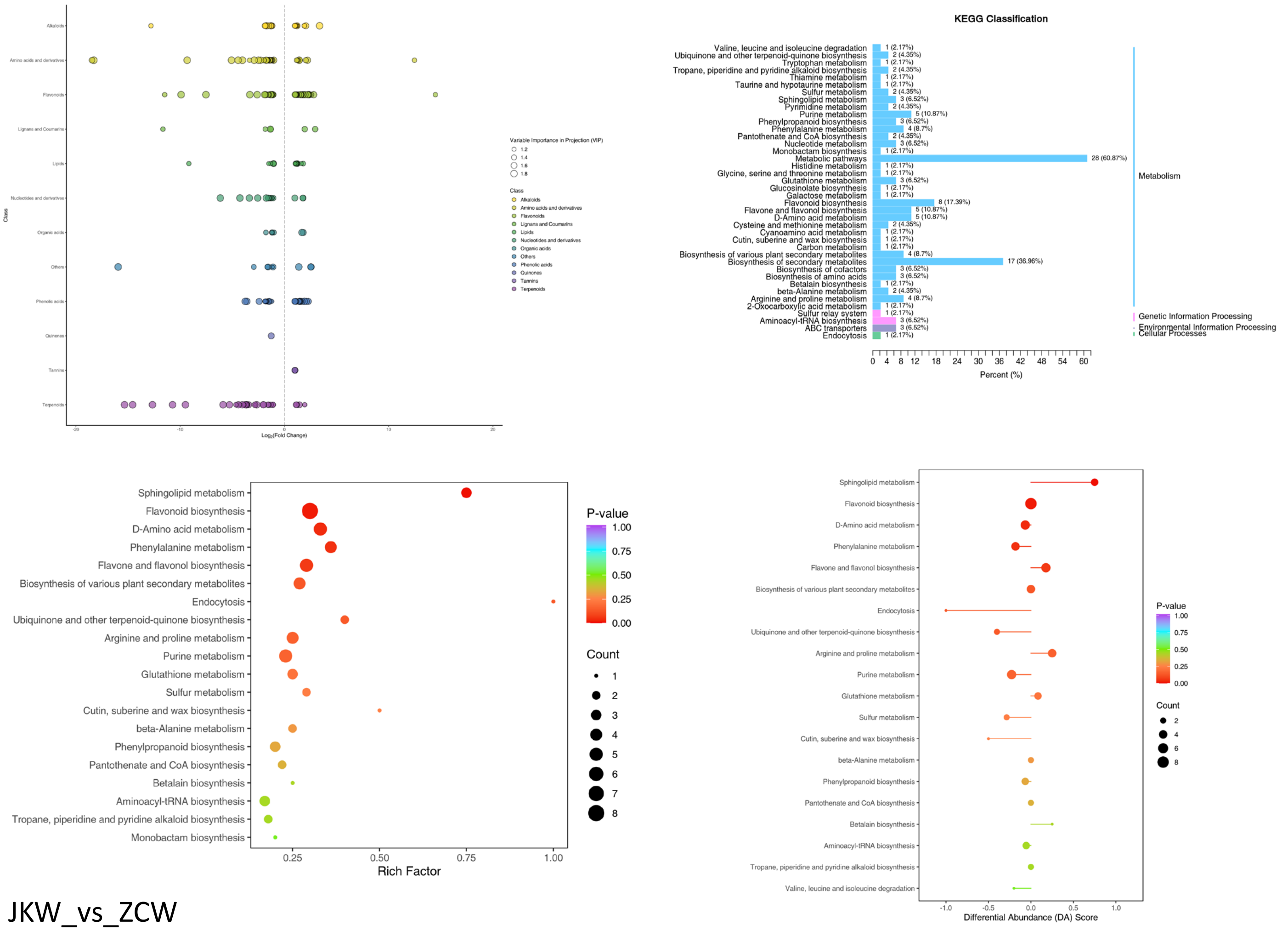


**Figure S5 | Pairwise differential metabolite analysis between *Ziziphus jujuba* cultivars. Scatter plot of differential metabolites shown on top, left**. Each dot in the graph indicates a metabolite, and different colors indicate different metabolite subclasses; the horizontal coordinate indicates the logarithmic value of the multiplicative difference in the content of a substance in two groups of samples (log22FC), the larger the absolute value of the horizontal coordinate, the greater the difference in the content of the substance between the two groups of samples, and the size of the dot represents the VIP value. **KEGG classification of differential metabolites on the top right**. The Y-axis shows the name of the KEGG pathway. The number of significant differential metabolites and the proportion of the total significant differential metabolites are shown next to the bar plot. **KEGG enrichment diagram of differential metabolites on bottom left.** The X-axis represents the Rich Factor and the Y-axis represents the pathway. The color of points reflects the p-value. The darker the red, the more significant the enrichment. The size of the dot represents the number of enriched differential metabolites. **Analysis of overall changes in KEGG metabolic pathway on bottom right.** The Y-axis represents the name of differential pathway, and the X-axis represents DA Score. DA Score reflects the overall change of all metabolites in the metabolic pathway. A Score of 1 indicates that the expression trend of all identified metabolites in this pathway is up-regulated, and -1 indicates that the expression trend of all identified metabolites in this pathway is down-regulated. The length of the line represent the absolute value of DA-score while the size of the dot at the end of the line represent the number of differential metabolites. A dot on the left of the line represent the pathway is down-regulated; a dot on the right of the line represents the pathway is up-regulated. The color of the line and dot represent the p-value. The darker the red, the smaller the p-value and the darker the purple, the larger the p-value.

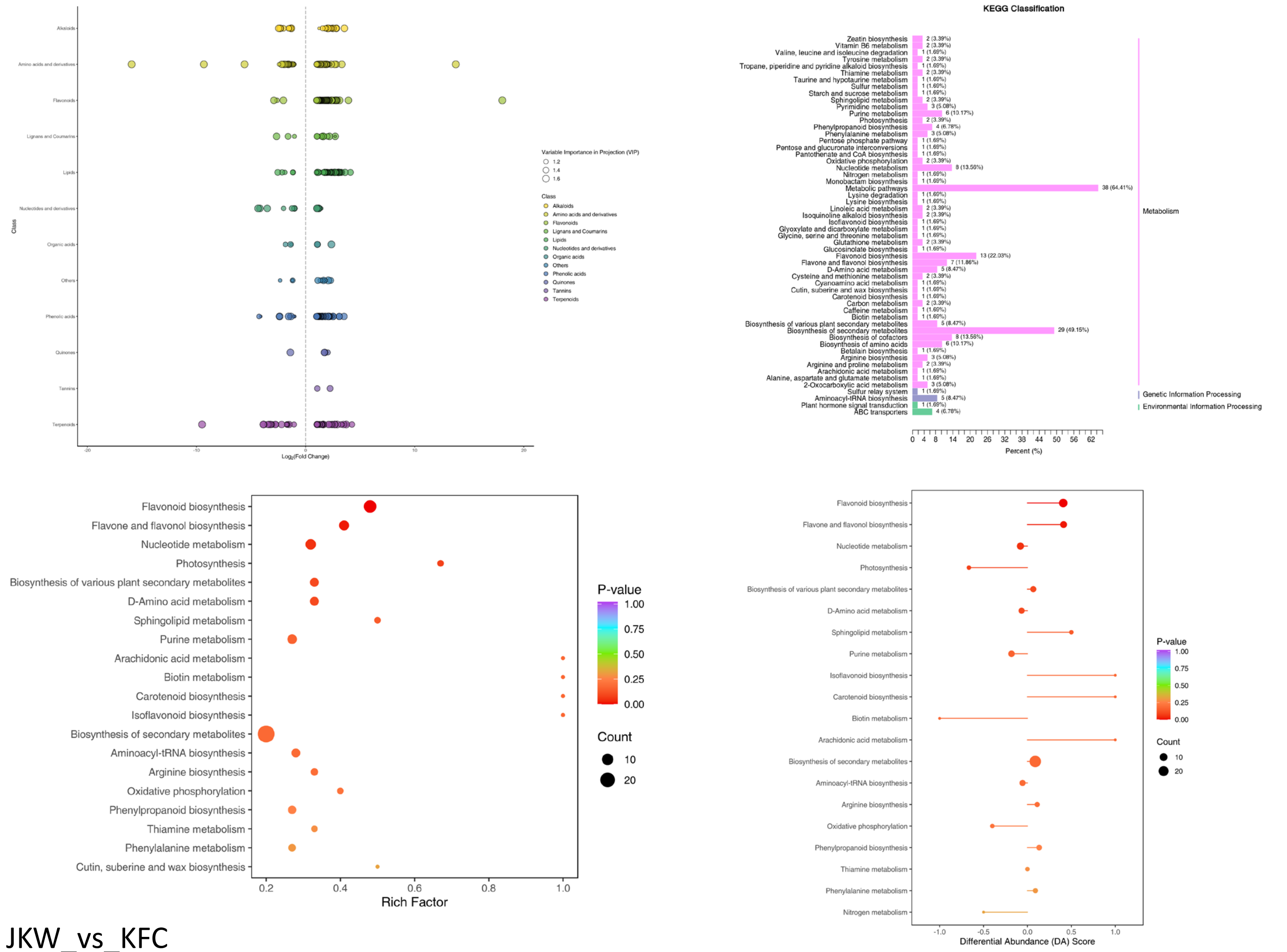


JKW_vs_KFC

**Figure S5 | Pairwise differential metabolite analysis between *Ziziphus jujuba* cultivars. Scatter plot of differential metabolites shown on top, left**. Each dot in the graph indicates a metabolite, and different colors indicate different metabolite subclasses; the horizontal coordinate indicates the logarithmic value of the multiplicative difference in the content of a substance in two groups of samples ($log_2 2FC$), the larger the absolute value of the horizontal coordinate, the greater the difference in the content of the substance between the two groups of samples, and the size of the dot represents the VIP value. **KEGG classification of differential metabolites on the top right**. The Y-axis shows the name of the KEGG pathway. The number of significant differential metabolites and the proportion of the total significant differential metabolites are shown next to the bar plot. **KEGG enrichment diagram of differential metabolites on bottom left.** The X-axis represents the Rich Factor and the Y-axis represents the pathway. The color of points reflects the p-value. The darker the red, the more significant the enrichment. The size of the dot represents the number of enriched differential metabolites. **Analysis of overall changes in KEGG metabolic pathway on bottom right.** The Y-axis represents the name of differential pathway, and the X-axis represents DA Score. DA Score reflects the overall change of all metabolites in the metabolic pathway. A Score of 1 indicates that the expression trend of all identified metabolites in this pathway is up-regulated, and -1 indicates that the expression trend of all identified metabolites in this pathway is down-regulated. The length of the line represent the absolute value of DA-score while the size of the dot at the end of the line represent the number of differential metabolites. A dot on the left of the line represent the pathway is down-regulated; a dot on the right of the line represents the pathway is up-regulated. The color of the line and dot represent the p-value. The darker the red, the smaller the p-value and the darker the purple, the larger the p-value.

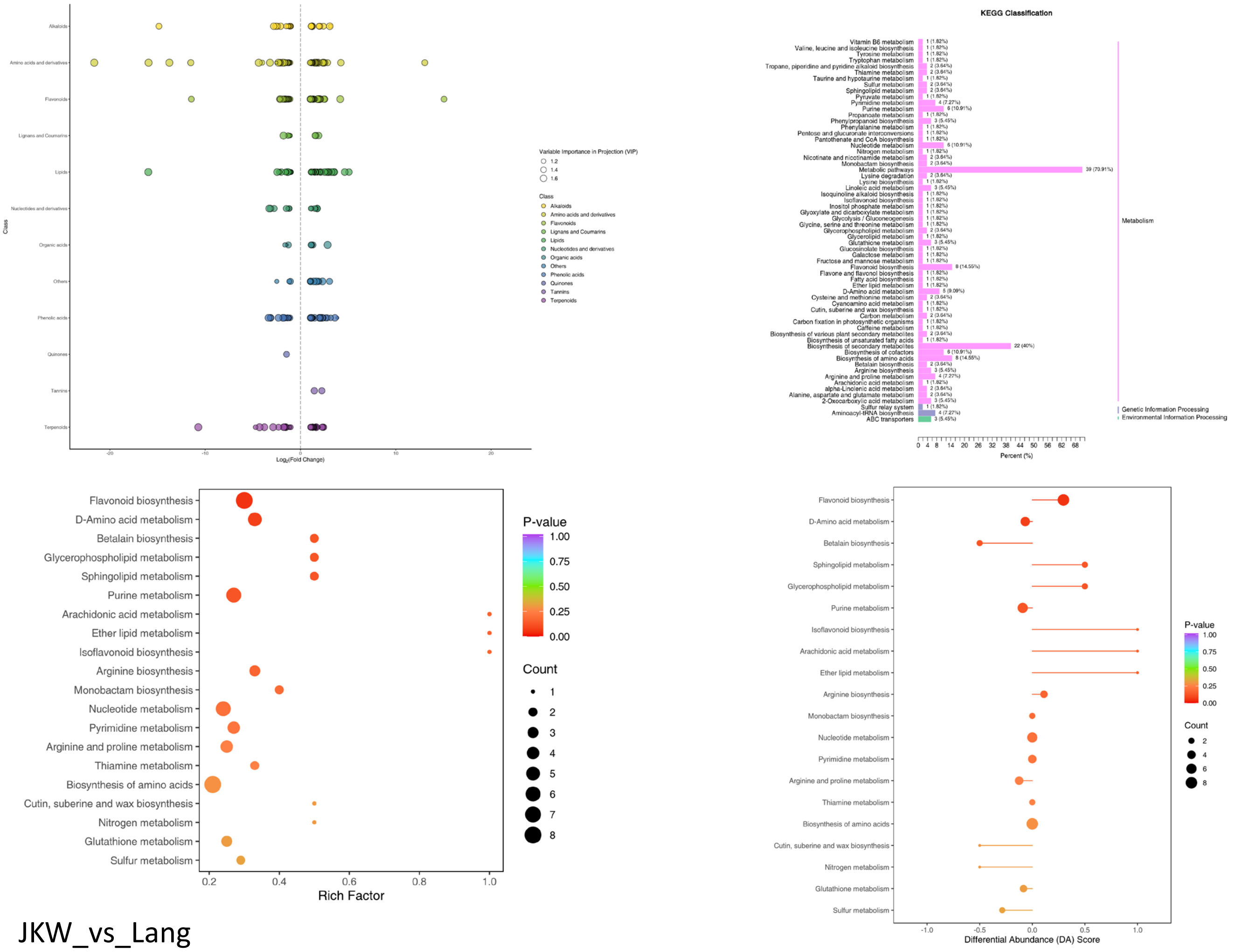


JKW_vs_Lang

**Figure S5 | Pairwise differential metabolite analysis between *Ziziphus jujuba* cultivars. Scatter plot of differential metabolites shown on top, left**. Each dot in the graph indicates a metabolite, and different colors indicate different metabolite subclasses; the horizontal coordinate indicates the logarithmic value of the multiplicative difference in the content of a substance in two groups of samples (log22FC), the larger the absolute value of the horizontal coordinate, the greater the difference in the content of the substance between the two groups of samples, and the size of the dot represents the VIP value. **KEGG classification of differential metabolites on the top right**. The Y-axis shows the name of the KEGG pathway. The number of significant differential metabolites and the proportion of the total significant differential metabolites are shown next to the bar plot. **KEGG enrichment diagram of differential metabolites on bottom left.** The X-axis represents the Rich Factor and the Y-axis represents the pathway. The color of points reflects the p-value. The darker the red, the more significant the enrichment. The size of the dot represents the number of enriched differential metabolites. **Analysis of overall changes in KEGG metabolic pathway on bottom right.** The Y-axis represents the name of differential pathway, and the X-axis represents DA Score. DA Score reflects the overall change of all metabolites in the metabolic pathway. A Score of 1 indicates that the expression trend of all identified metabolites in this pathway is up-regulated, and -1 indicates that the expression trend of all identified metabolites in this pathway is down-regulated. The length of the line represent the absolute value of DA-score while the size of the dot at the end of the line represent the number of differential metabolites. A dot on the left of the line represent the pathway is down-regulated; a dot on the right of the line represents the pathway is up-regulated. The color of the line and dot represent the p-value. The darker the red, the smaller the p-value and the darker the purple, the larger the p-value.

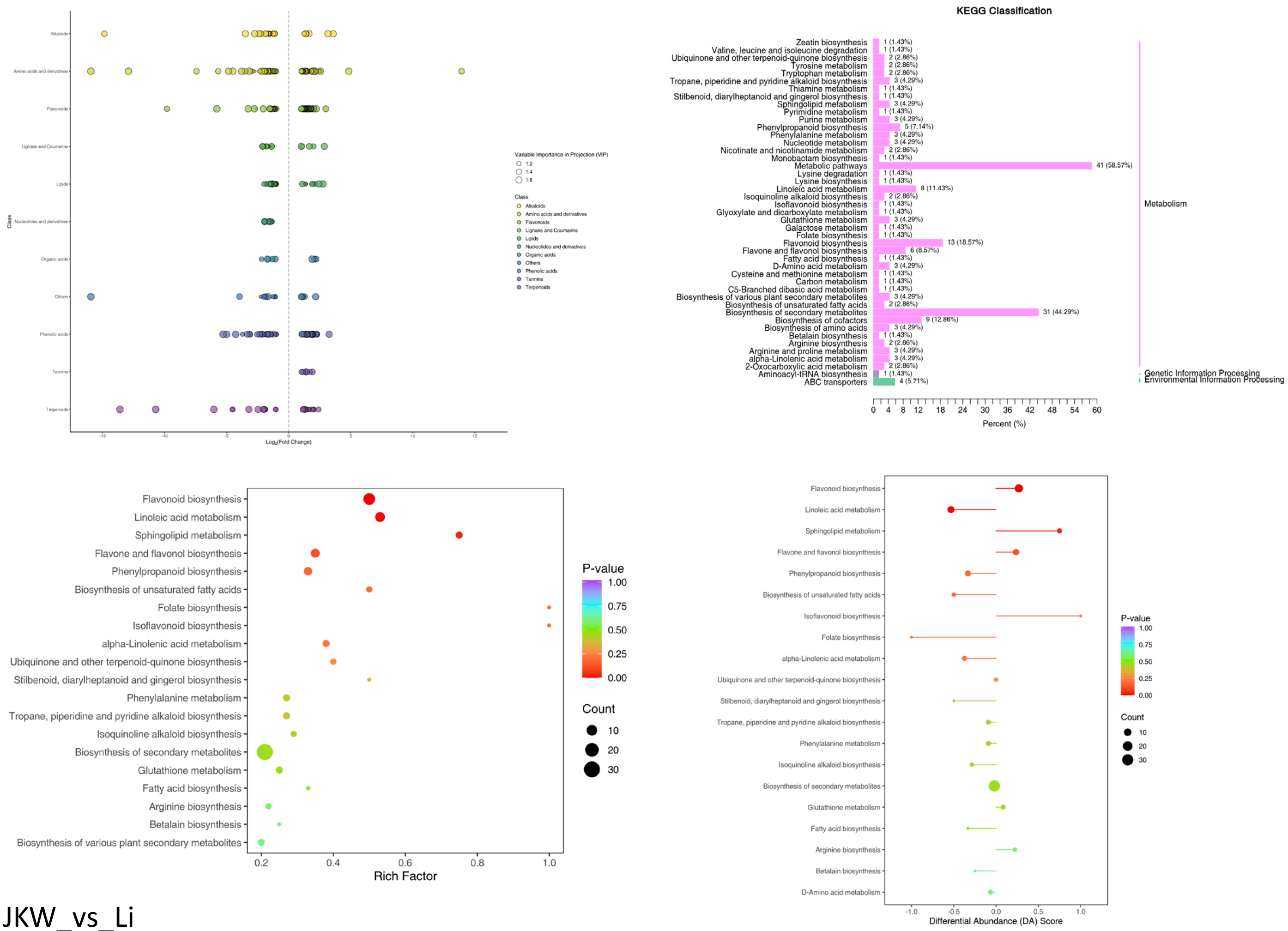


JKW_vs_Li

**Figure S5 | Pairwise differential metabolite analysis between *Ziziphus jujuba* cultivars. Scatter plot of differential metabolites shown on top, left**. Each dot in the graph indicates a metabolite, and different colors indicate different metabolite subclasses; the horizontal coordinate indicates the logarithmic value of the multiplicative difference in the content of a substance in two groups of samples (log22FC), the larger the absolute value of the horizontal coordinate, the greater the difference in the content of the substance between the two groups of samples, and the size of the dot represents the VIP value. **KEGG classification of differential metabolites on the top right**. The Y-axis shows the name of the KEGG pathway. The number of significant differential metabolites and the proportion of the total significant differential metabolites are shown next to the bar plot. **KEGG enrichment diagram of differential metabolites on bottom left.** The X-axis represents the Rich Factor and the Y-axis represents the pathway. The color of points reflects the p-value. The darker the red, the more significant the enrichment. The size of the dot represents the number of enriched differential metabolites. **Analysis of overall changes in KEGG metabolic pathway on bottom right.** The Y-axis represents the name of differential pathway, and the X-axis represents DA Score. DA Score reflects the overall change of all metabolites in the metabolic pathway. A Score of 1 indicates that the expression trend of all identified metabolites in this pathway is up-regulated, and -1 indicates that the expression trend of all identified metabolites in this pathway is down-regulated. The length of the line represent the absolute value of DA-score while the size of the dot at the end of the line represent the number of differential metabolites. A dot on the left of the line represent the pathway is down-regulated; a dot on the right of the line represents the pathway is up-regulated. The color of the line and dot represent the p-value. The darker the red, the smaller the p-value and the darker the purple, the larger the p-value.

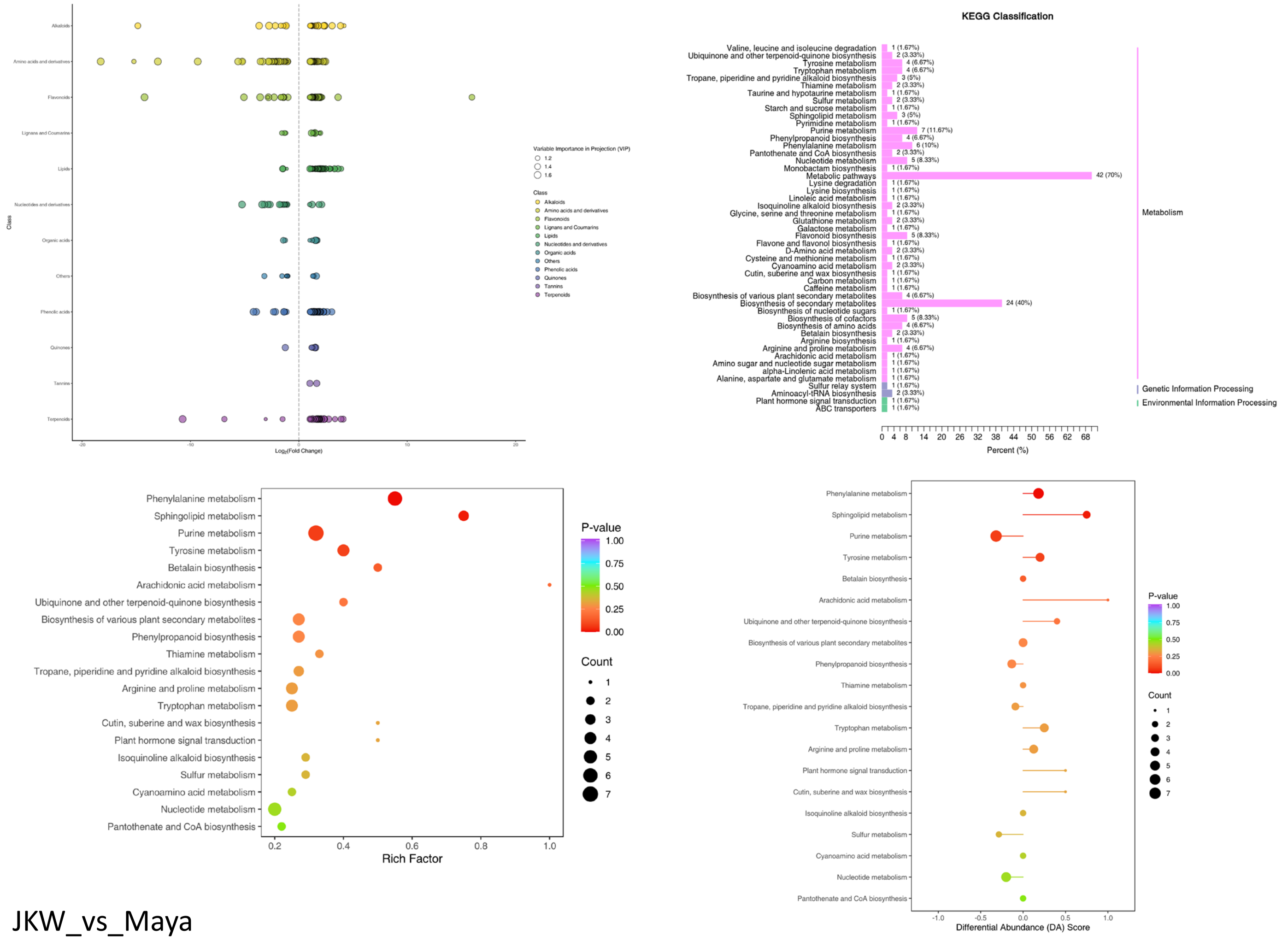


**Figure S5 | Pairwise differential metabolite analysis between *Ziziphus jujuba* cultivars. Scatter plot of differential metabolites shown on top, left**. Each dot in the graph indicates a metabolite, and different colors indicate different metabolite subclasses; the horizontal coordinate indicates the logarithmic value of the multiplicative difference in the content of a substance in two groups of samples (log22FC), the larger the absolute value of the horizontal coordinate, the greater the difference in the content of the substance between the two groups of samples, and the size of the dot represents the VIP value. **KEGG classification of differential metabolites on the top right**. The Y-axis shows the name of the KEGG pathway. The number of significant differential metabolites and the proportion of the total significant differential metabolites are shown next to the bar plot. **KEGG enrichment diagram of differential metabolites on bottom left.** The X-axis represents the Rich Factor and the Y-axis represents the pathway. The color of points reflects the p-value. The darker the red, the more significant the enrichment. The size of the dot represents the number of enriched differential metabolites. **Analysis of overall changes in KEGG metabolic pathway on bottom right.** The Y-axis represents the name of differential pathway, and the X-axis represents DA Score. DA Score reflects the overall change of all metabolites in the metabolic pathway. A Score of 1 indicates that the expression trend of all identified metabolites in this pathway is up-regulated, and -1 indicates that the expression trend of all identified metabolites in this pathway is down-regulated. The length of the line represent the absolute value of DA-score while the size of the dot at the end of the line represent the number of differential metabolites. A dot on the left of the line represent the pathway is down-regulated; a dot on the right of the line represents the pathway is up-regulated. The color of the line and dot represent the p-value. The darker the red, the smaller the p-value and the darker the purple, the larger the p-value.

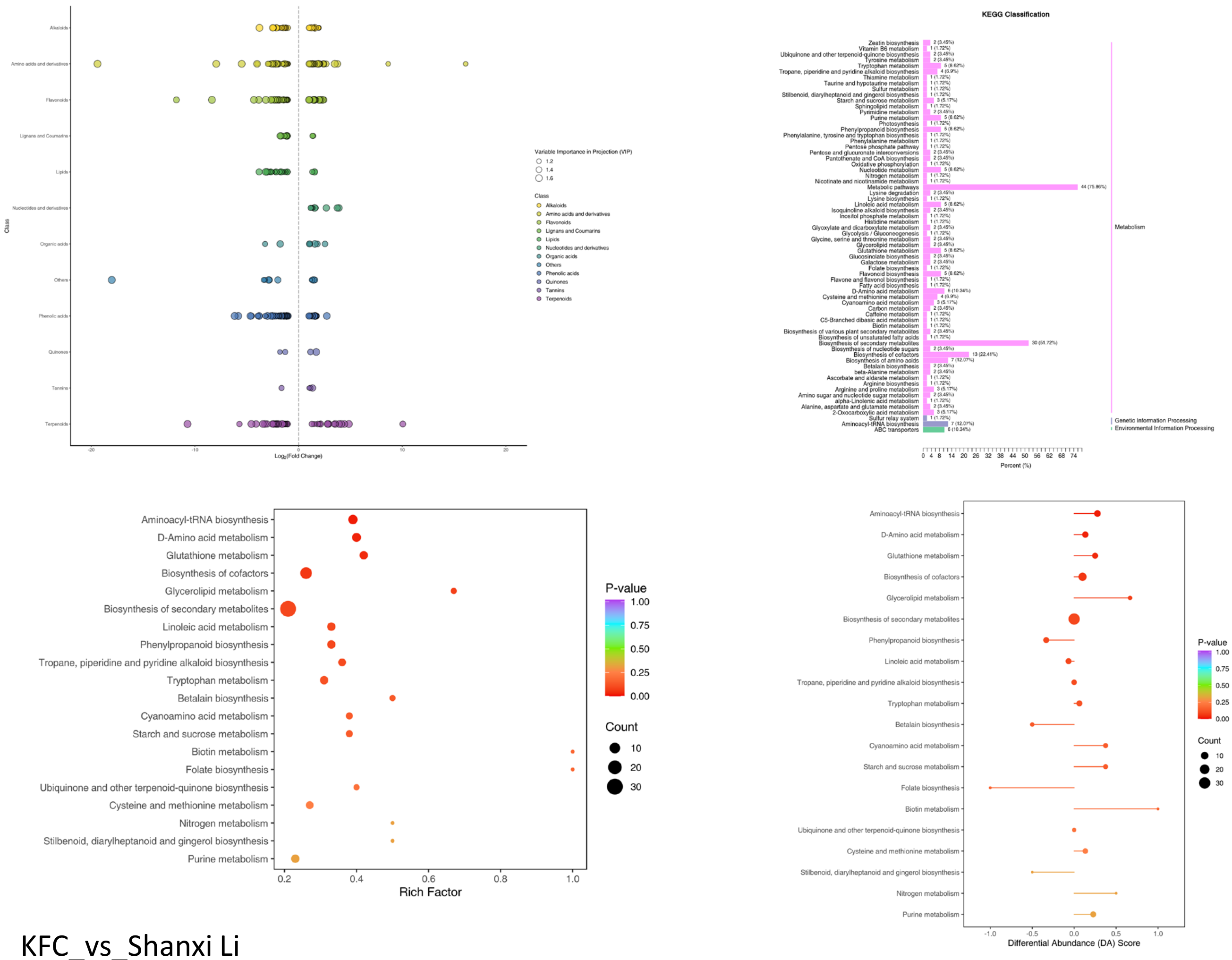


KFC_vs_Shanxi Li

**Figure S5 | Pairwise differential metabolite analysis between *Ziziphus jujuba* cultivars. Scatter plot of differential metabolites shown on top, left**. Each dot in the graph indicates a metabolite, and different colors indicate different metabolite subclasses; the horizontal coordinate indicates the logarithmic value of the multiplicative difference in the content of a substance in two groups of samples (log22FC), the larger the absolute value of the horizontal coordinate, the greater the difference in the content of the substance between the two groups of samples, and the size of the dot represents the VIP value. **KEGG classification of differential metabolites on the top right**. The Y-axis shows the name of the KEGG pathway. The number of significant differential metabolites and the proportion of the total significant differential metabolites are shown next to the bar plot. **KEGG enrichment diagram of differential metabolites on bottom left.** The X-axis represents the Rich Factor and the Y-axis represents the pathway. The color of points reflects the p-value. The darker the red, the more significant the enrichment. The size of the dot represents the number of enriched differential metabolites. **Analysis of overall changes in KEGG metabolic pathway on bottom right.** The Y-axis represents the name of differential pathway, and the X-axis represents DA Score. DA Score reflects the overall change of all metabolites in the metabolic pathway. A Score of 1 indicates that the expression trend of all identified metabolites in this pathway is up-regulated, and -1 indicates that the expression trend of all identified metabolites in this pathway is down-regulated. The length of the line represent the absolute value of DA-score while the size of the dot at the end of the line represent the number of differential metabolites. A dot on the left of the line represent the pathway is down-regulated; a dot on the right of the line represents the pathway is up-regulated. The color of the line and dot represent the p-value. The darker the red, the smaller the p-value and the darker the purple, the larger the p-value.

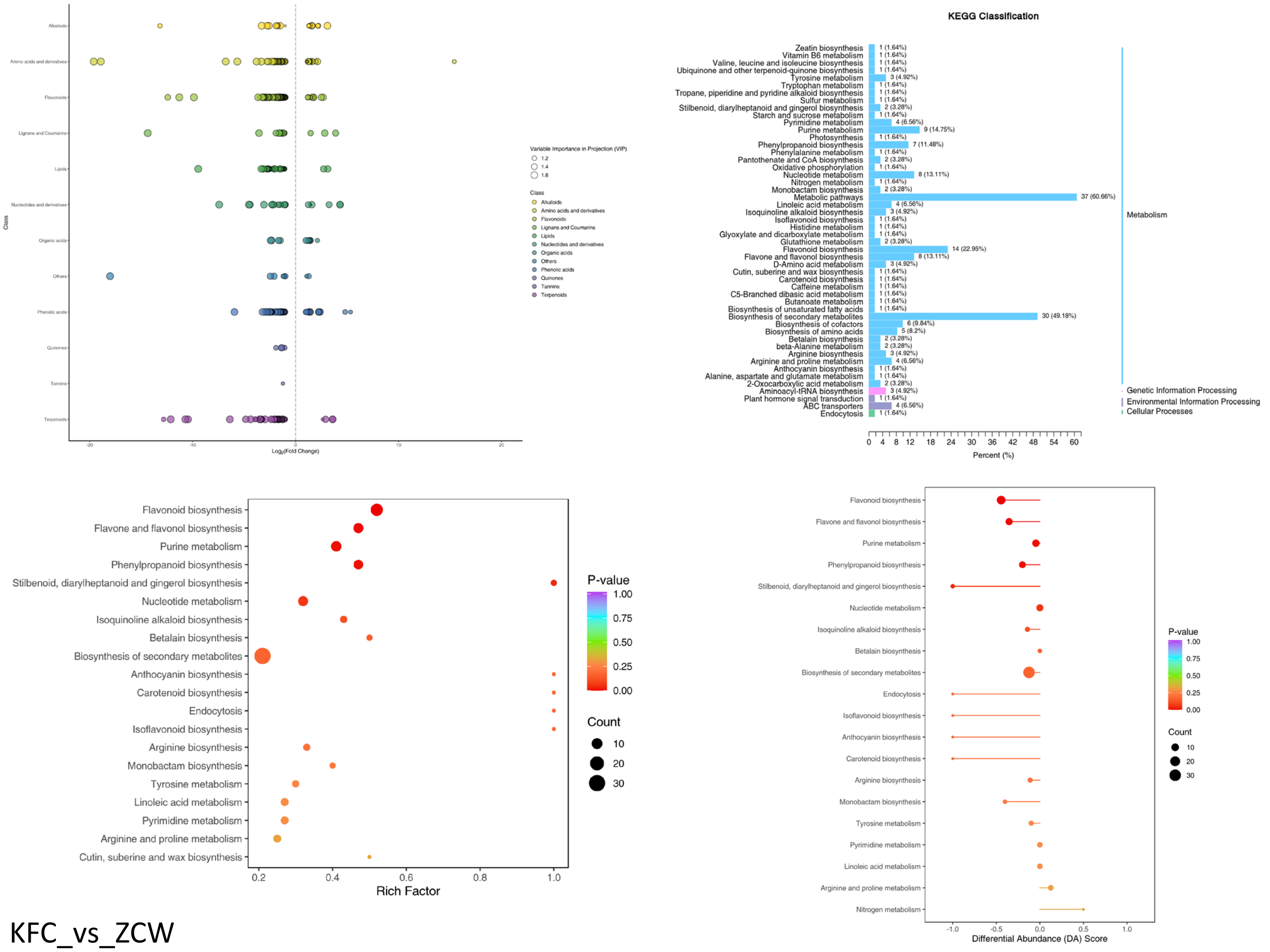


KFC_vs_ZCW

**Figure S5 | Pairwise differential metabolite analysis between *Ziziphus jujuba* cultivars. Scatter plot of differential metabolites shown on top, left**. Each dot in the graph indicates a metabolite, and different colors indicate different metabolite subclasses; the horizontal coordinate indicates the logarithmic value of the multiplicative difference in the content of a substance in two groups of samples (log22FC), the larger the absolute value of the horizontal coordinate, the greater the difference in the content of the substance between the two groups of samples, and the size of the dot represents the VIP value. **KEGG classification of differential metabolites on the top right**. The Y-axis shows the name of the KEGG pathway. The number of significant differential metabolites and the proportion of the total significant differential metabolites are shown next to the bar plot. **KEGG enrichment diagram of differential metabolites on bottom left.** The X-axis represents the Rich Factor and the Y-axis represents the pathway. The color of points reflects the p-value. The darker the red, the more significant the enrichment. The size of the dot represents the number of enriched differential metabolites. **Analysis of overall changes in KEGG metabolic pathway on bottom right.** The Y-axis represents the name of differential pathway, and the X-axis represents DA Score. DA Score reflects the overall change of all metabolites in the metabolic pathway. A Score of 1 indicates that the expression trend of all identified metabolites in this pathway is up-regulated, and -1 indicates that the expression trend of all identified metabolites in this pathway is down-regulated. The length of the line represent the absolute value of DA-score while the size of the dot at the end of the line represent the number of differential metabolites. A dot on the left of the line represent the pathway is down-regulated; a dot on the right of the line represents the pathway is up-regulated. The color of the line and dot represent the p-value. The darker the red, the smaller the p-value and the darker the purple, the larger the p-value.

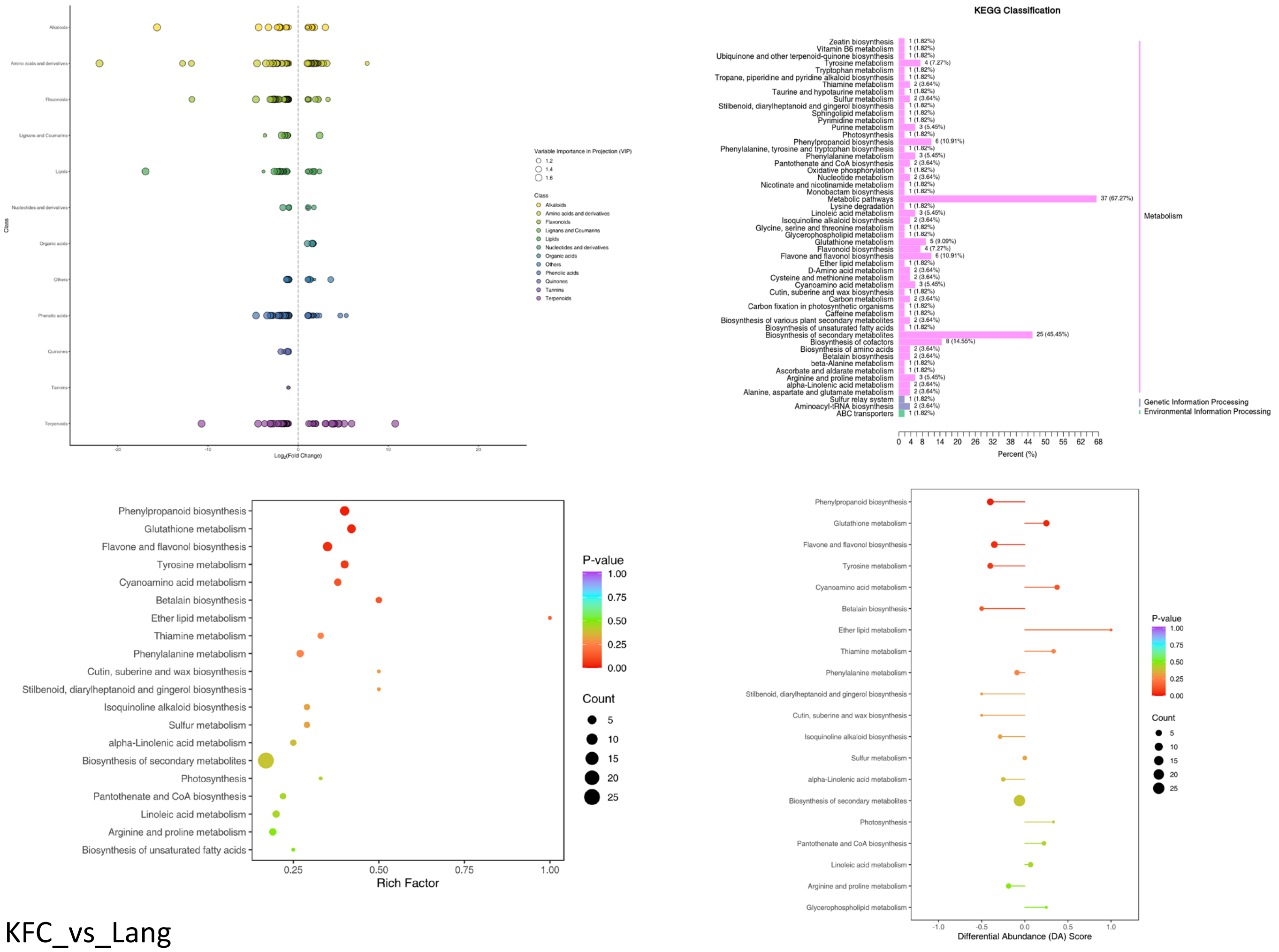


**Figure S5 | Pairwise differential metabolite analysis between *Ziziphus jujuba* cultivars. Scatter plot of differential metabolites shown on top, left**. Each dot in the graph indicates a metabolite, and different colors indicate different metabolite subclasses; the horizontal coordinate indicates the logarithmic value of the multiplicative difference in the content of a substance in two groups of samples (log22FC), the larger the absolute value of the horizontal coordinate, the greater the difference in the content of the substance between the two groups of samples, and the size of the dot represents the VIP value. **KEGG classification of differential metabolites on the top right**. The Y-axis shows the name of the KEGG pathway. The number of significant differential metabolites and the proportion of the total significant differential metabolites are shown next to the bar plot. **KEGG enrichment diagram of differential metabolites on bottom left.** The X-axis represents the Rich Factor and the Y-axis represents the pathway. The color of points reflects the p-value. The darker the red, the more significant the enrichment. The size of the dot represents the number of enriched differential metabolites. **Analysis of overall changes in KEGG metabolic pathway on bottom right.** The Y-axis represents the name of differential pathway, and the X-axis represents DA Score. DA Score reflects the overall change of all metabolites in the metabolic pathway. A Score of 1 indicates that the expression trend of all identified metabolites in this pathway is up-regulated, and -1 indicates that the expression trend of all identified metabolites in this pathway is down-regulated. The length of the line represent the absolute value of DA-score while the size of the dot at the end of the line represent the number of differential metabolites. A dot on the left of the line represent the pathway is down-regulated; a dot on the right of the line represents the pathway is up-regulated. The color of the line and dot represent the p-value. The darker the red, the smaller the p-value and the darker the purple, the larger the p-value.

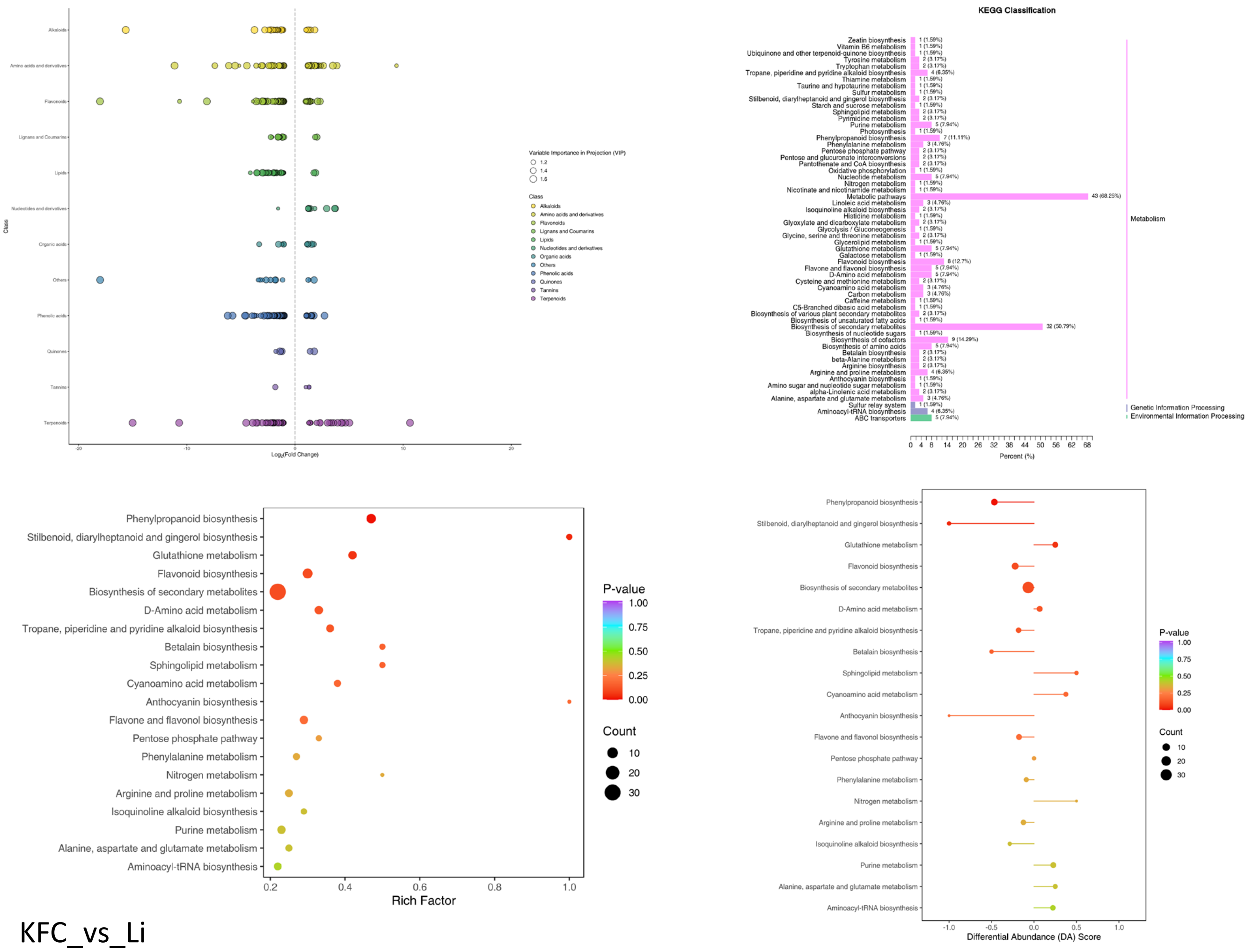


**Figure S5 | Pairwise differential metabolite analysis between *Ziziphus jujuba* cultivars. Scatter plot of differential metabolites shown on top, left**. Each dot in the graph indicates a metabolite, and different colors indicate different metabolite subclasses; the horizontal coordinate indicates the logarithmic value of the multiplicative difference in the content of a substance in two groups of samples (log22FC), the larger the absolute value of the horizontal coordinate, the greater the difference in the content of the substance between the two groups of samples, and the size of the dot represents the VIP value. **KEGG classification of differential metabolites on the top right**. The Y-axis shows the name of the KEGG pathway. The number of significant differential metabolites and the proportion of the total significant differential metabolites are shown next to the bar plot. **KEGG enrichment diagram of differential metabolites on bottom left.** The X-axis represents the Rich Factor and the Y-axis represents the pathway. The color of points reflects the p-value. The darker the red, the more significant the enrichment. The size of the dot represents the number of enriched differential metabolites. **Analysis of overall changes in KEGG metabolic pathway on bottom right.** The Y-axis represents the name of differential pathway, and the X-axis represents DA Score. DA Score reflects the overall change of all metabolites in the metabolic pathway. A Score of 1 indicates that the expression trend of all identified metabolites in this pathway is up-regulated, and -1 indicates that the expression trend of all identified metabolites in this pathway is down-regulated. The length of the line represent the absolute value of DA-score while the size of the dot at the end of the line represent the number of differential metabolites. A dot on the left of the line represent the pathway is down-regulated; a dot on the right of the line represents the pathway is up-regulated. The color of the line and dot represent the p-value. The darker the red, the smaller the p-value and the darker the purple, the larger the p-value.

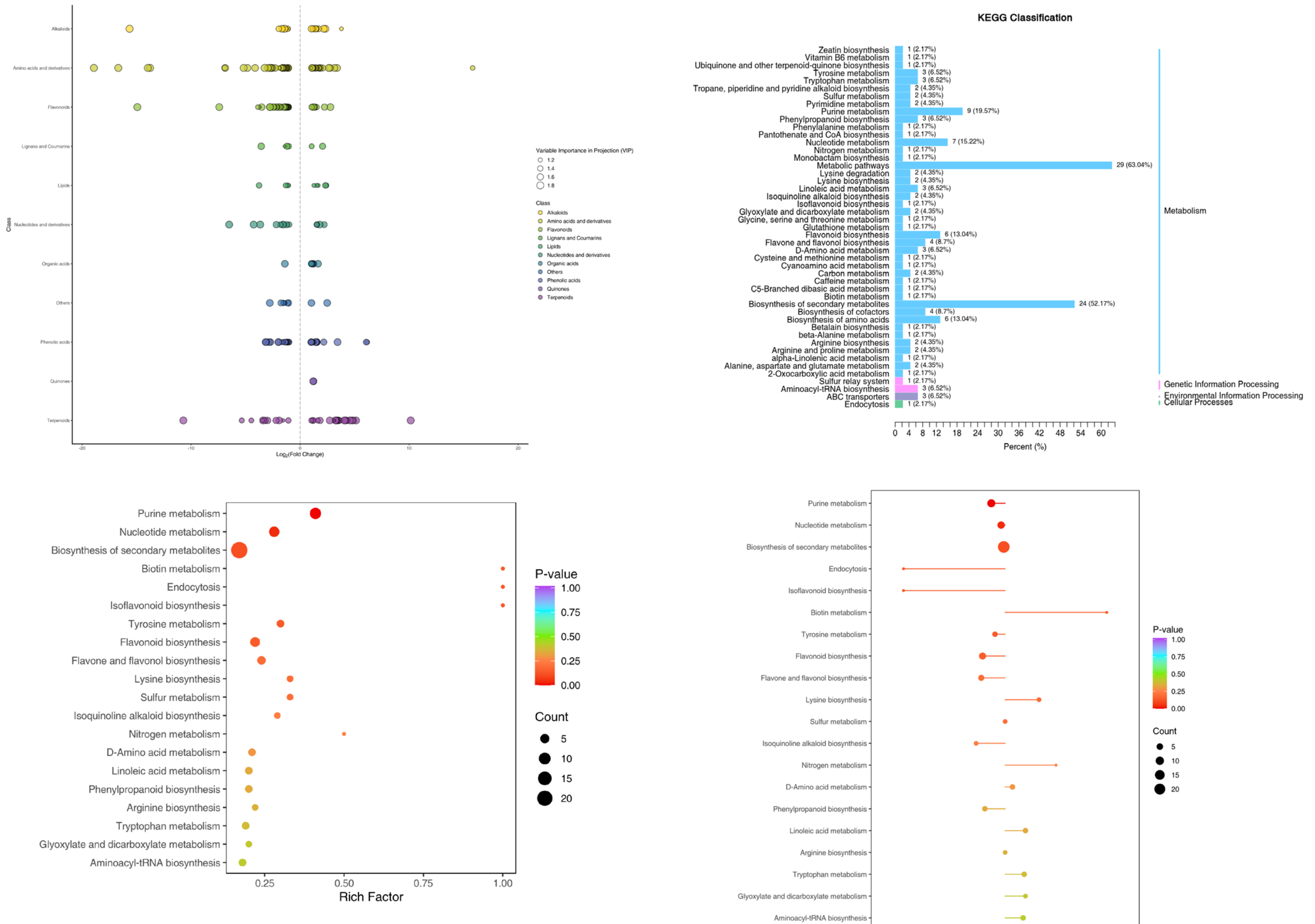


KFC_vs_Maya

**Figure S5 | Pairwise differential metabolite analysis between *Ziziphus jujuba* cultivars. Scatter plot of differential metabolites shown on top, left**. Each dot in the graph indicates a metabolite, and different colors indicate different metabolite subclasses; the horizontal coordinate indicates the logarithmic value of the multiplicative difference in the content of a substance in two groups of samples ($\log_2 2FC$), the larger the absolute value of the horizontal coordinate, the greater the difference in the content of the substance between the two groups of samples, and the size of the dot represents the VIP value. **KEGG classification of differential metabolites on the top right**. The Y-axis shows the name of the KEGG pathway. The number of significant differential metabolites and the proportion of the total significant differential metabolites are shown next to the bar plot. **KEGG enrichment diagram of differential metabolites on bottom left.** The X-axis represents the Rich Factor and the Y-axis represents the pathway. The color of points reflects the p-value. The darker the red, the more significant the enrichment. The size of the dot represents the number of enriched differential metabolites. **Analysis of overall changes in KEGG metabolic pathway on bottom right.** The Y-axis represents the name of differential pathway, and the X-axis represents DA Score. DA Score reflects the overall change of all metabolites in the metabolic pathway. A Score of 1 indicates that the expression trend of all identified metabolites in this pathway is up-regulated, and -1 indicates that the expression trend of all identified metabolites in this pathway is down-regulated. The length of the line represent the absolute value of DA-score while the size of the dot at the end of the line represent the number of differential metabolites. A dot on the left of the line represent the pathway is down-regulated; a dot on the right of the line represents the pathway is up-regulated. The color of the line and dot represent the p-value. The darker the red, the smaller the p-value and the darker the purple, the larger the p-value.

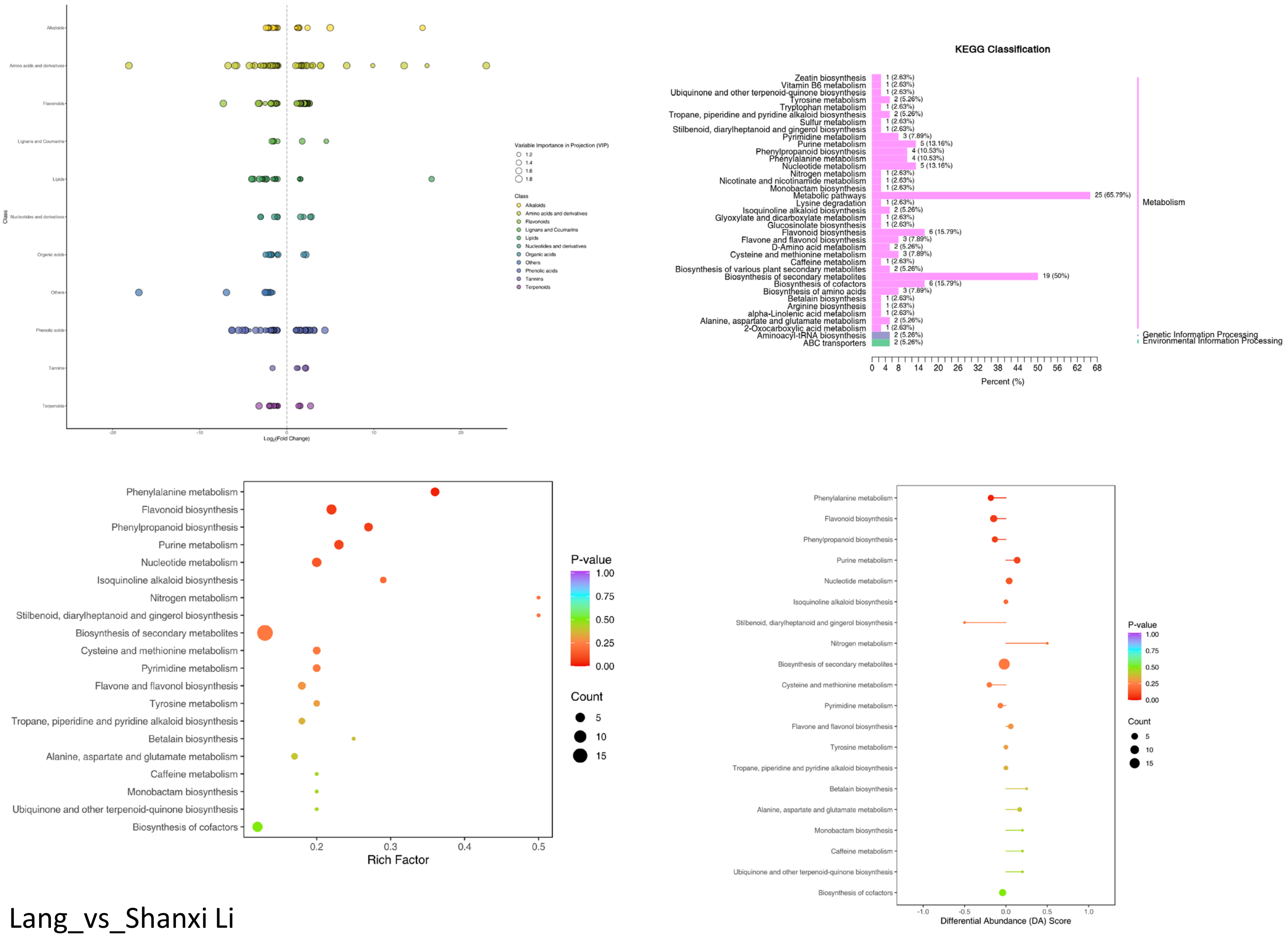


Lang_vs_Shanxi Li

**Figure S5 | Pairwise differential metabolite analysis between *Ziziphus jujuba* cultivars. Scatter plot of differential metabolites shown on top, left**. Each dot in the graph indicates a metabolite, and different colors indicate different metabolite subclasses; the horizontal coordinate indicates the logarithmic value of the multiplicative difference in the content of a substance in two groups of samples (log22FC), the larger the absolute value of the horizontal coordinate, the greater the difference in the content of the substance between the two groups of samples, and the size of the dot represents the VIP value. **KEGG classification of differential metabolites on the top right**. The Y-axis shows the name of the KEGG pathway. The number of significant differential metabolites and the proportion of the total significant differential metabolites are shown next to the bar plot. **KEGG enrichment diagram of differential metabolites on bottom left.** The X-axis represents the Rich Factor and the Y-axis represents the pathway. The color of points reflects the p-value. The darker the red, the more significant the enrichment. The size of the dot represents the number of enriched differential metabolites. **Analysis of overall changes in KEGG metabolic pathway on bottom right.** The Y-axis represents the name of differential pathway, and the X-axis represents DA Score. DA Score reflects the overall change of all metabolites in the metabolic pathway. A Score of 1 indicates that the expression trend of all identified metabolites in this pathway is up-regulated, and -1 indicates that the expression trend of all identified metabolites in this pathway is down-regulated. The length of the line represent the absolute value of DA-score while the size of the dot at the end of the line represent the number of differential metabolites. A dot on the left of the line represent the pathway is down-regulated; a dot on the right of the line represents the pathway is up-regulated. The color of the line and dot represent the p-value. The darker the red, the smaller the p-value and the darker the purple, the larger the p-value.

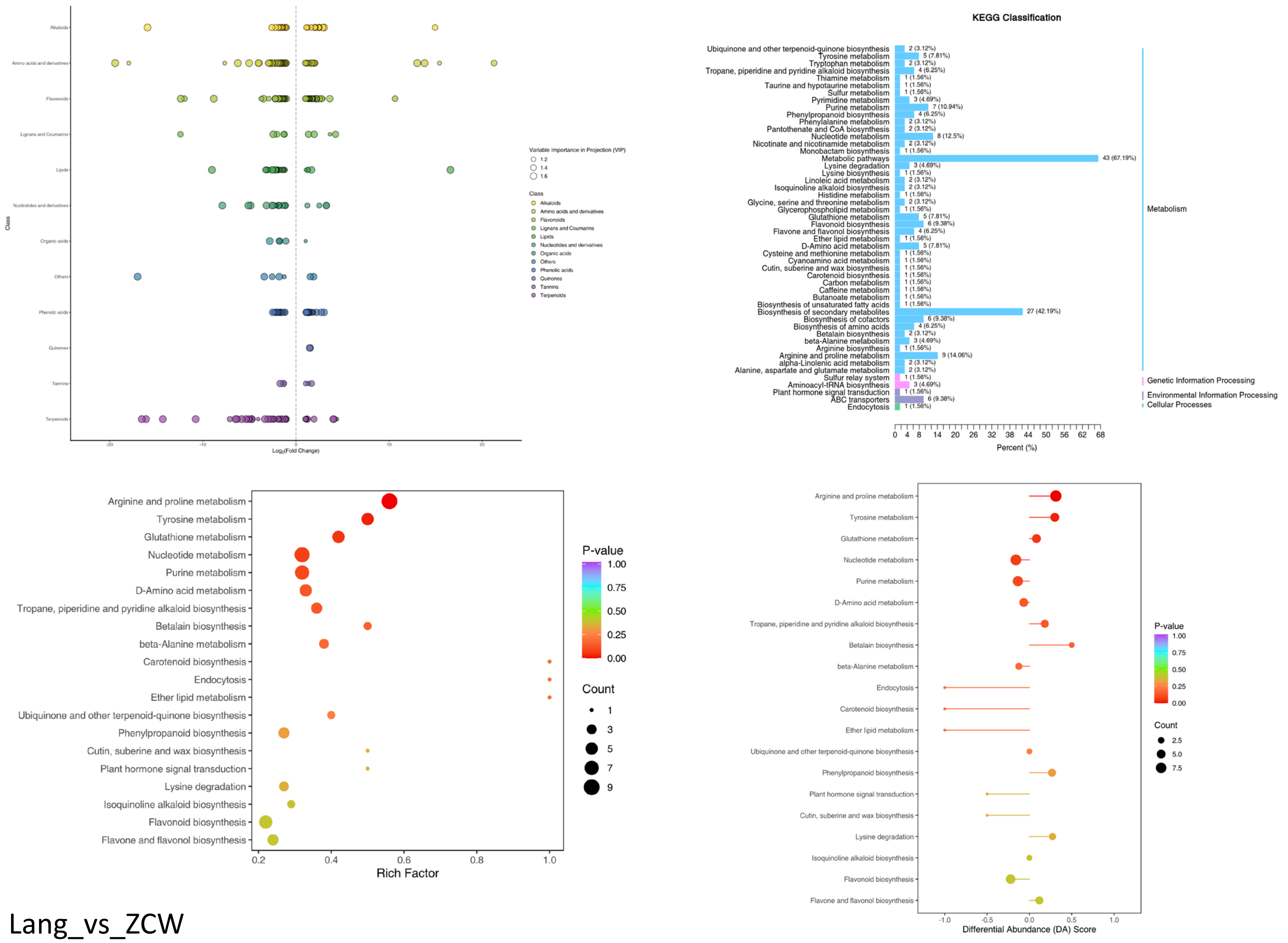


Lang_vs_ZCW

**Figure S5 | Pairwise differential metabolite analysis between *Ziziphus jujuba* cultivars. Scatter plot of differential metabolites shown on top, left**. Each dot in the graph indicates a metabolite, and different colors indicate different metabolite subclasses; the horizontal coordinate indicates the logarithmic value of the multiplicative difference in the content of a substance in two groups of samples ($log_2$2FC), the larger the absolute value of the horizontal coordinate, the greater the difference in the content of the substance between the two groups of samples, and the size of the dot represents the VIP value. **KEGG classification of differential metabolites on the top right**. The Y-axis shows the name of the KEGG pathway. The number of significant differential metabolites and the proportion of the total significant differential metabolites are shown next to the bar plot. **KEGG enrichment diagram of differential metabolites on bottom left.** The X-axis represents the Rich Factor and the Y-axis represents the pathway. The color of points reflects the p-value. The darker the red, the more significant the enrichment. The size of the dot represents the number of enriched differential metabolites. **Analysis of overall changes in KEGG metabolic pathway on bottom right.** The Y-axis represents the name of differential pathway, and the X-axis represents DA Score. DA Score reflects the overall change of all metabolites in the metabolic pathway. A Score of 1 indicates that the expression trend of all identified metabolites in this pathway is up-regulated, and -1 indicates that the expression trend of all identified metabolites in this pathway is down-regulated. The length of the line represent the absolute value of DA-score while the size of the dot at the end of the line represent the number of differential metabolites. A dot on the left of the line represent the pathway is down-regulated; a dot on the right of the line represents the pathway is up-regulated. The color of the line and dot represent the p-value. The darker the red, the smaller the p-value and the darker the purple, the larger the p-value.

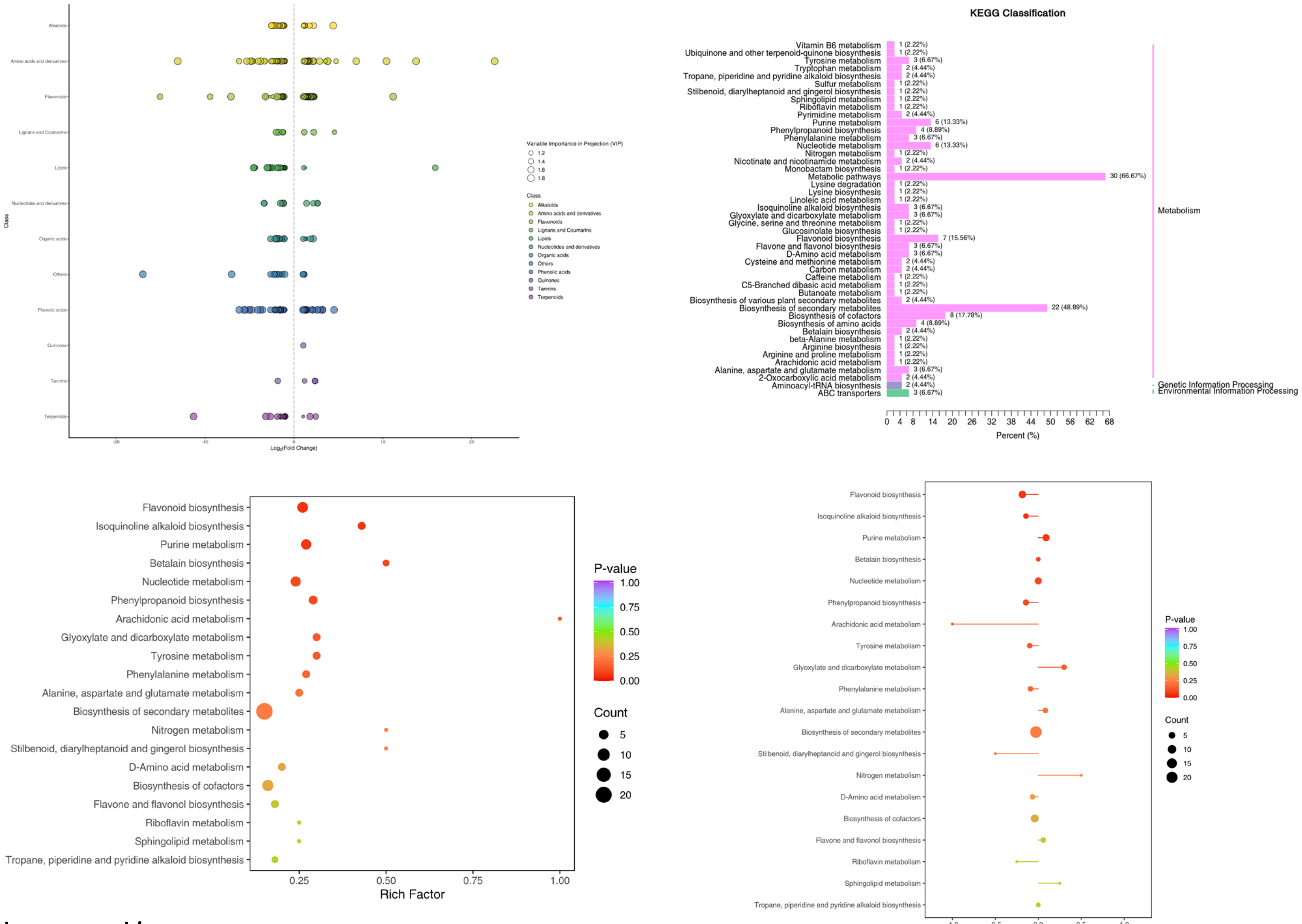


Lang_vs_Li

**Figure S5 | Pairwise differential metabolite analysis between *Ziziphus jujuba* cultivars. Scatter plot of differential metabolites shown on top, left**. Each dot in the graph indicates a metabolite, and different colors indicate different metabolite subclasses; the horizontal coordinate indicates the logarithmic value of the multiplicative difference in the content of a substance in two groups of samples (log22FC), the larger the absolute value of the horizontal coordinate, the greater the difference in the content of the substance between the two groups of samples, and the size of the dot represents the VIP value. **KEGG classification of differential metabolites on the top right**. The Y-axis shows the name of the KEGG pathway. The number of significant differential metabolites and the proportion of the total significant differential metabolites are shown next to the bar plot. **KEGG enrichment diagram of differential metabolites on bottom left.** The X-axis represents the Rich Factor and the Y-axis represents the pathway. The color of points reflects the p-value. The darker the red, the more significant the enrichment. The size of the dot represents the number of enriched differential metabolites. **Analysis of overall changes in KEGG metabolic pathway on bottom right.** The Y-axis represents the name of differential pathway, and the X-axis represents DA Score. DA Score reflects the overall change of all metabolites in the metabolic pathway. A Score of 1 indicates that the expression trend of all identified metabolites in this pathway is up-regulated, and -1 indicates that the expression trend of all identified metabolites in this pathway is down-regulated. The length of the line represent the absolute value of DA-score while the size of the dot at the end of the line represent the number of differential metabolites. A dot on the left of the line represent the pathway is down-regulated; a dot on the right of the line represents the pathway is up-regulated. The color of the line and dot represent the p-value. The darker the red, the smaller the p-value and the darker the purple, the larger the p-value.

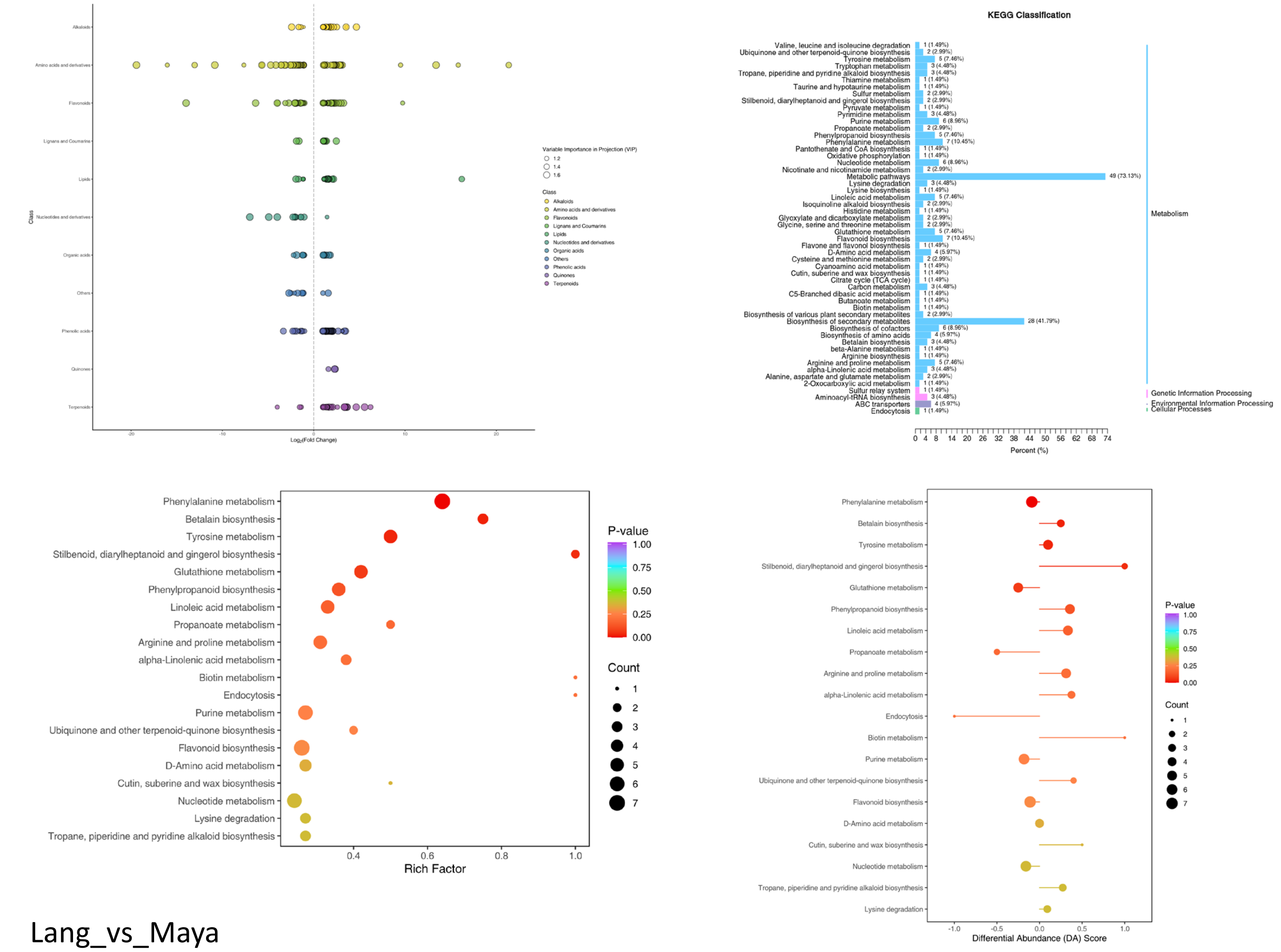


Lang_vs_Maya

**Figure S5 | Pairwise differential metabolite analysis between *Ziziphus jujuba* cultivars. Scatter plot of differential metabolites shown on top, left**. Each dot in the graph indicates a metabolite, and different colors indicate different metabolite subclasses; the horizontal coordinate indicates the logarithmic value of the multiplicative difference in the content of a substance in two groups of samples (log22FC), the larger the absolute value of the horizontal coordinate, the greater the difference in the content of the substance between the two groups of samples, and the size of the dot represents the VIP value. **KEGG classification of differential metabolites on the top right**. The Y-axis shows the name of the KEGG pathway. The number of significant differential metabolites and the proportion of the total significant differential metabolites are shown next to the bar plot. **KEGG enrichment diagram of differential metabolites on bottom left.** The X-axis represents the Rich Factor and the Y-axis represents the pathway. The color of points reflects the p-value. The darker the red, the more significant the enrichment. The size of the dot represents the number of enriched differential metabolites. **Analysis of overall changes in KEGG metabolic pathway on bottom right.** The Y-axis represents the name of differential pathway, and the X-axis represents DA Score. DA Score reflects the overall change of all metabolites in the metabolic pathway. A Score of 1 indicates that the expression trend of all identified metabolites in this pathway is up-regulated, and -1 indicates that the expression trend of all identified metabolites in this pathway is down-regulated. The length of the line represent the absolute value of DA-score while the size of the dot at the end of the line represent the number of differential metabolites. A dot on the left of the line represent the pathway is down-regulated; a dot on the right of the line represents the pathway is up-regulated. The color of the line and dot represent the p-value. The darker the red, the smaller the p-value and the darker the purple, the larger the p-value.

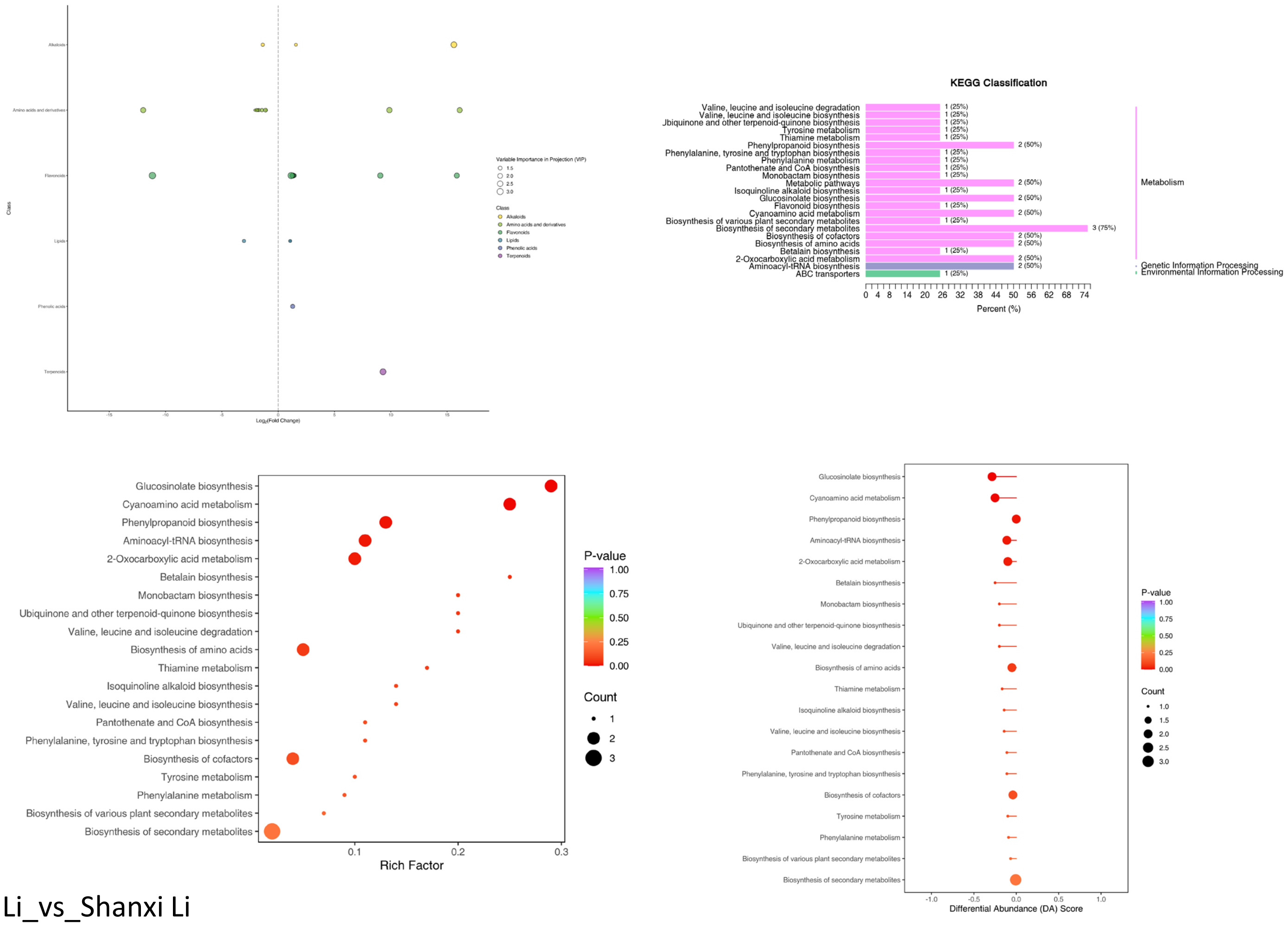


**Figure S5 | Pairwise differential metabolite analysis between *Ziziphus jujuba* cultivars. Scatter plot of differential metabolites shown on top, left**. Each dot in the graph indicates a metabolite, and different colors indicate different metabolite subclasses; the horizontal coordinate indicates the logarithmic value of the multiplicative difference in the content of a substance in two groups of samples (log2FC), the larger the absolute value of the horizontal coordinate, the greater the difference in the content of the substance between the two groups of samples, and the size of the dot represents the VIP value. **KEGG classification of differential metabolites on the top right**. The Y-axis shows the name of the KEGG pathway. The number of significant differential metabolites and the proportion of the total significant differential metabolites are shown next to the bar plot. **KEGG enrichment diagram of differential metabolites on bottom left.** The X-axis represents the Rich Factor and the Y-axis represents the pathway. The color of points reflects the p-value. The darker the red, the more significant the enrichment. The size of the dot represents the number of enriched differential metabolites. **Analysis of overall changes in KEGG metabolic pathway on bottom right.** The Y-axis represents the name of differential pathway, and the X-axis represents DA Score. DA Score reflects the overall change of all metabolites in the metabolic pathway. A Score of 1 indicates that the expression trend of all identified metabolites in this pathway is up-regulated, and -1 indicates that the expression trend of all identified metabolites in this pathway is down-regulated. The length of the line represent the absolute value of DA-score while the size of the dot at the end of the line represent the number of differential metabolites. A dot on the left of the line represent the pathway is down-regulated; a dot on the right of the line represents the pathway is up-regulated. The color of the line and dot represent the p-value. The darker the red, the smaller the p-value and the darker the purple, the larger the p-value.

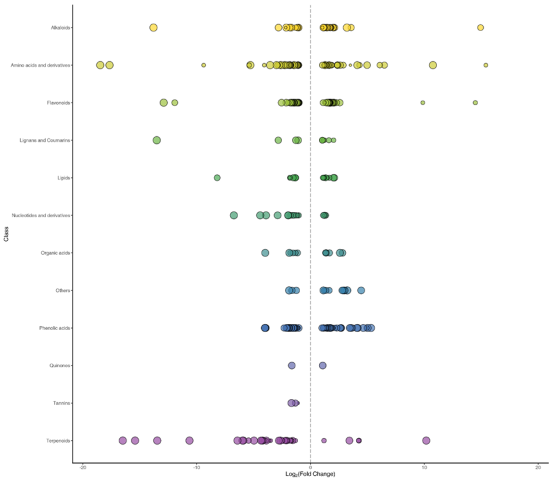


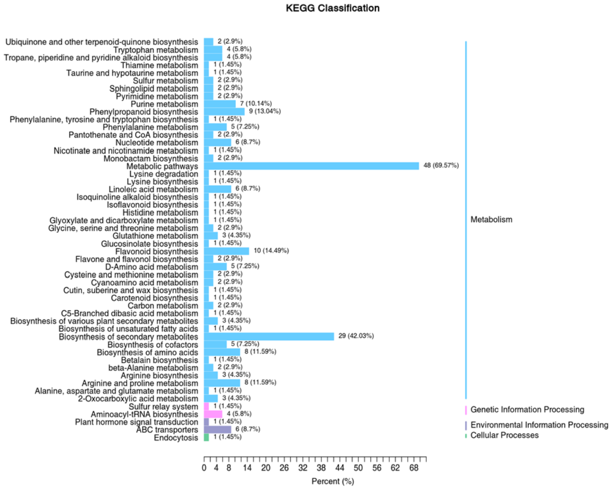


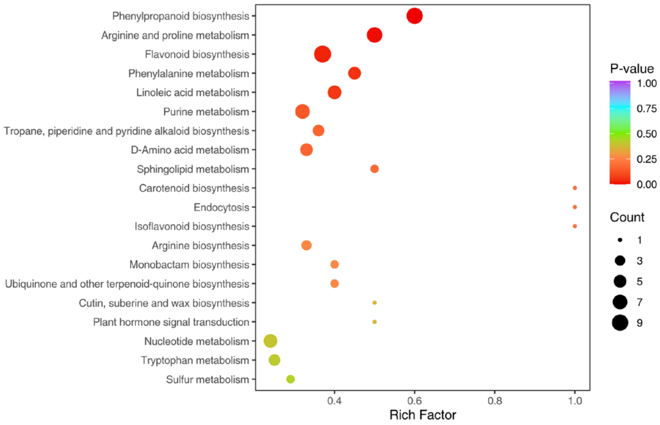


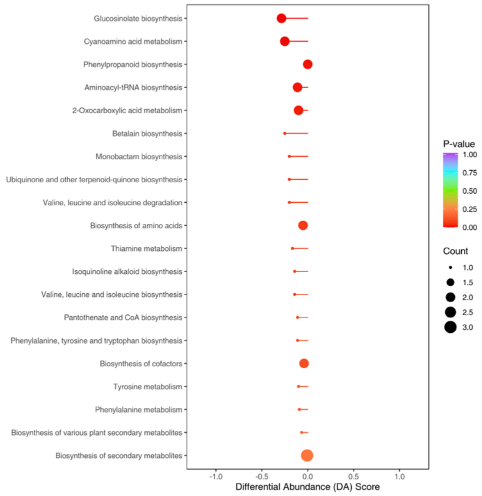


Li_vs_ZCW

**Figure S5 | Pairwise differential metabolite analysis between *Ziziphus jujuba* cultivars. Scatter plot of differential metabolites shown on top, left**. Each dot in the graph indicates a metabolite, and different colors indicate different metabolite subclasses; the horizontal coordinate indicates the logarithmic value of the multiplicative difference in the content of a substance in two groups of samples (log22FC), the larger the absolute value of the horizontal coordinate, the greater the difference in the content of the substance between the two groups of samples, and the size of the dot represents the VIP value. **KEGG classification of differential metabolites on the top right**. The Y-axis shows the name of the KEGG pathway. The number of significant differential metabolites and the proportion of the total significant differential metabolites are shown next to the bar plot. **KEGG enrichment diagram of differential metabolites on bottom left.** The X-axis represents the Rich Factor and the Y-axis represents the pathway. The color of points reflects the p-value. The darker the red, the more significant the enrichment. The size of the dot represents the number of enriched differential metabolites. **Analysis of overall changes in KEGG metabolic pathway on bottom right.** The Y-axis represents the name of differential pathway, and the X-axis represents DA Score. DA Score reflects the overall change of all metabolites in the metabolic pathway. A Score of 1 indicates that the expression trend of all identified metabolites in this pathway is up-regulated, and -1 indicates that the expression trend of all identified metabolites in this pathway is down-regulated. The length of the line represent the absolute value of DA-score while the size of the dot at the end of the line represent the number of differential metabolites. A dot on the left of the line represent the pathway is down-regulated; a dot on the right of the line represents the pathway is up-regulated. The color of the line and dot represent the p-value. The darker the red, the smaller the p-value and the darker the purple, the larger the p-value.

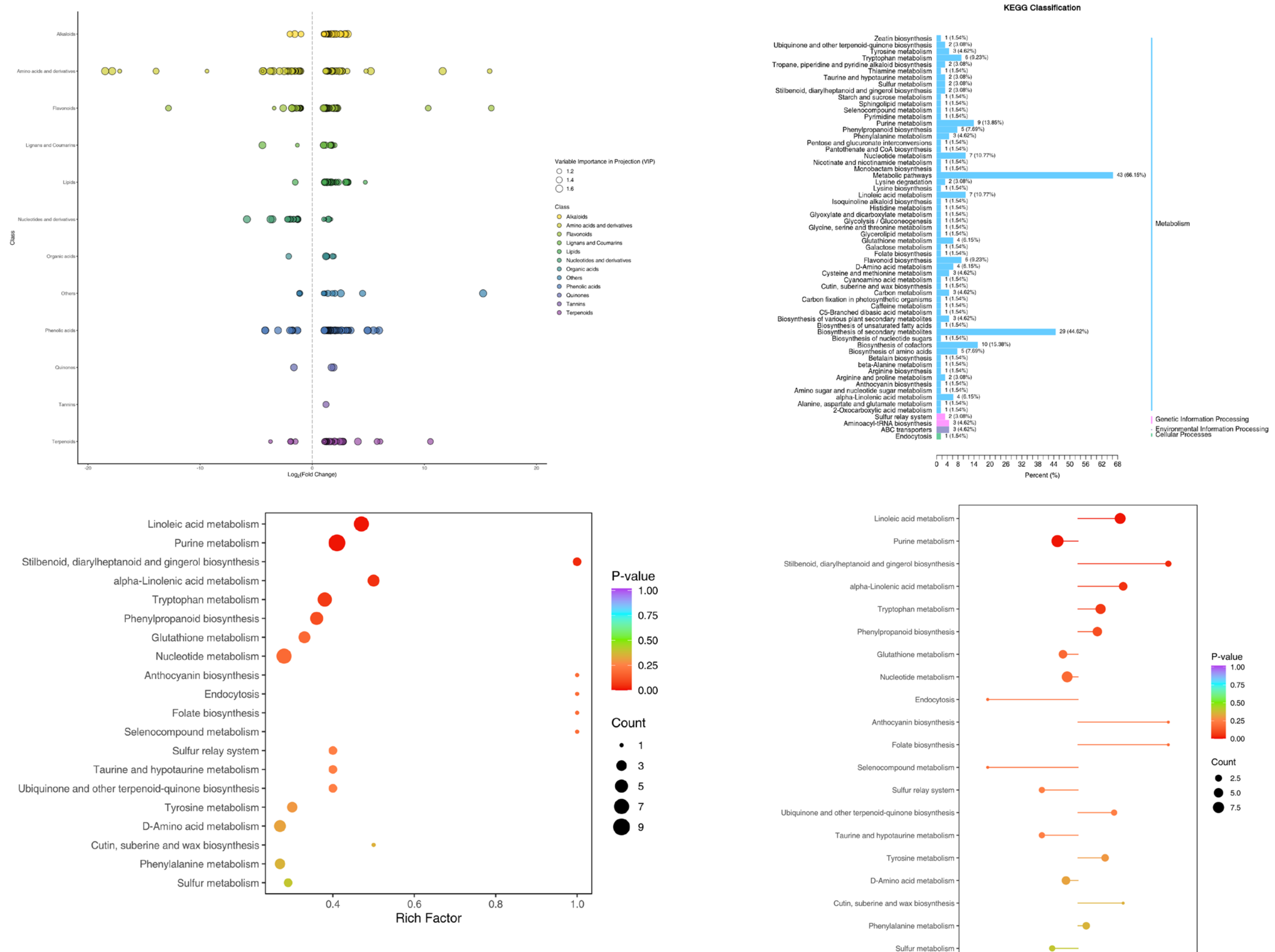


Li_vs_Maya

**Figure S5 | Pairwise differential metabolite analysis between *Ziziphus jujuba* cultivars. Scatter plot of differential metabolites shown on top, left**. Each dot in the graph indicates a metabolite, and different colors indicate different metabolite subclasses; the horizontal coordinate indicates the logarithmic value of the multiplicative difference in the content of a substance in two groups of samples (log22FC), the larger the absolute value of the horizontal coordinate, the greater the difference in the content of the substance between the two groups of samples, and the size of the dot represents the VIP value. **KEGG classification of differential metabolites on the top right**. The Y-axis shows the name of the KEGG pathway. The number of significant differential metabolites and the proportion of the total significant differential metabolites are shown next to the bar plot. **KEGG enrichment diagram of differential metabolites on bottom left.** The X-axis represents the Rich Factor and the Y-axis represents the pathway. The color of points reflects the p-value. The darker the red, the more significant the enrichment. The size of the dot represents the number of enriched differential metabolites. **Analysis of overall changes in KEGG metabolic pathway on bottom right.** The Y-axis represents the name of differential pathway, and the X-axis represents DA Score. DA Score reflects the overall change of all metabolites in the metabolic pathway. A Score of 1 indicates that the expression trend of all identified metabolites in this pathway is up-regulated, and -1 indicates that the expression trend of all identified metabolites in this pathway is down-regulated. The length of the line represent the absolute value of DA-score while the size of the dot at the end of the line represent the number of differential metabolites. A dot on the left of the line represent the pathway is down-regulated; a dot on the right of the line represents the pathway is up-regulated. The color of the line and dot represent the p-value. The darker the red, the smaller the p-value and the darker the purple, the larger the p-value.

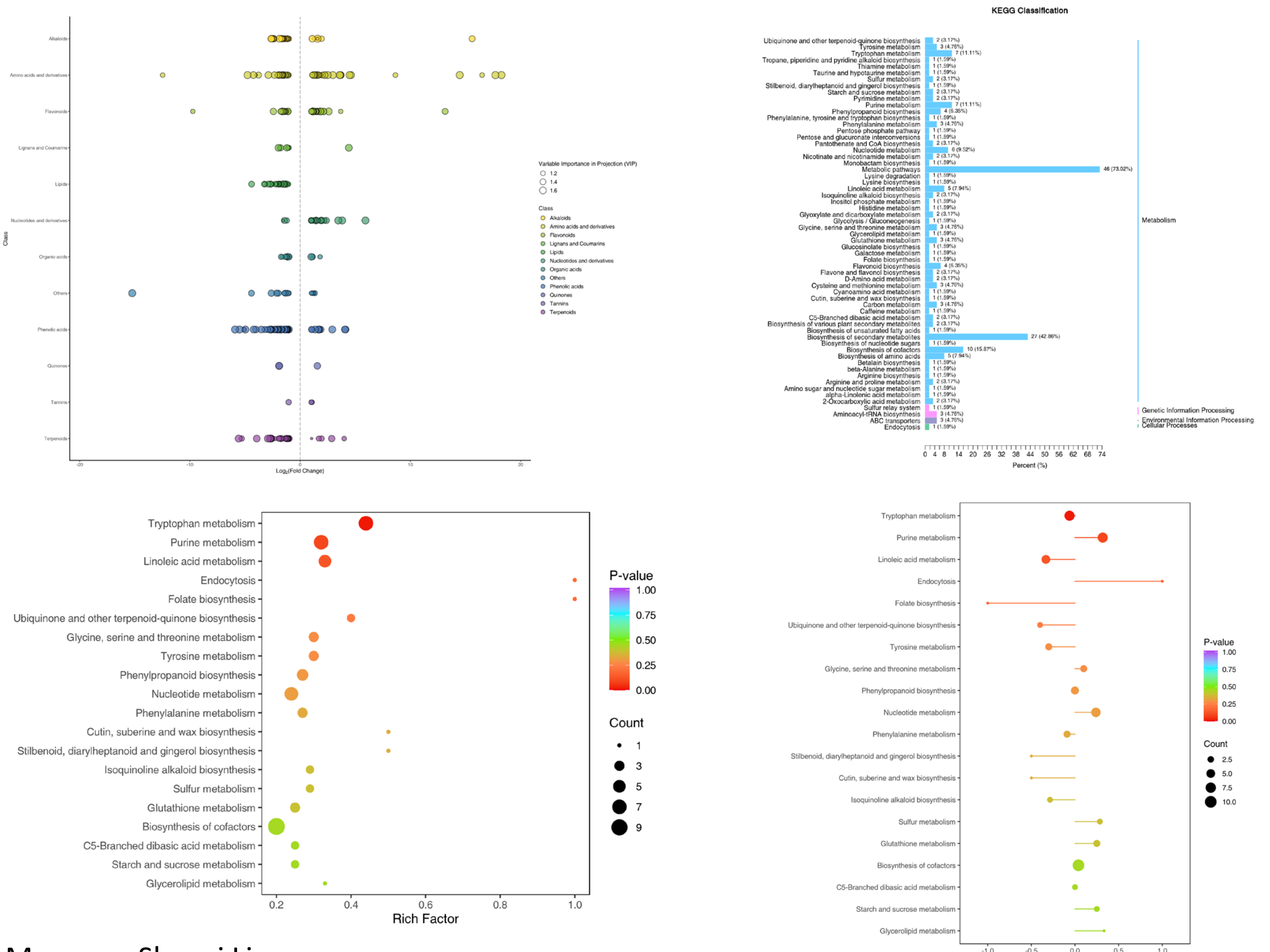


Maya_vs_Shanxi Li

**Figure S5 | Pairwise differential metabolite analysis between *Ziziphus jujuba* cultivars. Scatter plot of differential metabolites shown on top, left**. Each dot in the graph indicates a metabolite, and different colors indicate different metabolite subclasses; the horizontal coordinate indicates the logarithmic value of the multiplicative difference in the content of a substance in two groups of samples (log22FC), the larger the absolute value of the horizontal coordinate, the greater the difference in the content of the substance between the two groups of samples, and the size of the dot represents the VIP value. **KEGG classification of differential metabolites on the top right**. The Y-axis shows the name of the KEGG pathway. The number of significant differential metabolites and the proportion of the total significant differential metabolites are shown next to the bar plot. **KEGG enrichment diagram of differential metabolites on bottom left.** The X-axis represents the Rich Factor and the Y-axis represents the pathway. The color of points reflects the p-value. The darker the red, the more significant the enrichment. The size of the dot represents the number of enriched differential metabolites. **Analysis of overall changes in KEGG metabolic pathway on bottom right.** The Y-axis represents the name of differential pathway, and the X-axis represents DA Score. DA Score reflects the overall change of all metabolites in the metabolic pathway. A Score of 1 indicates that the expression trend of all identified metabolites in this pathway is up-regulated, and -1 indicates that the expression trend of all identified metabolites in this pathway is down-regulated. The length of the line represent the absolute value of DA-score while the size of the dot at the end of the line represent the number of differential metabolites. A dot on the left of the line represent the pathway is down-regulated; a dot on the right of the line represents the pathway is up-regulated. The color of the line and dot represent the p-value. The darker the red, the smaller the p-value and the darker the purple, the larger the p-value.

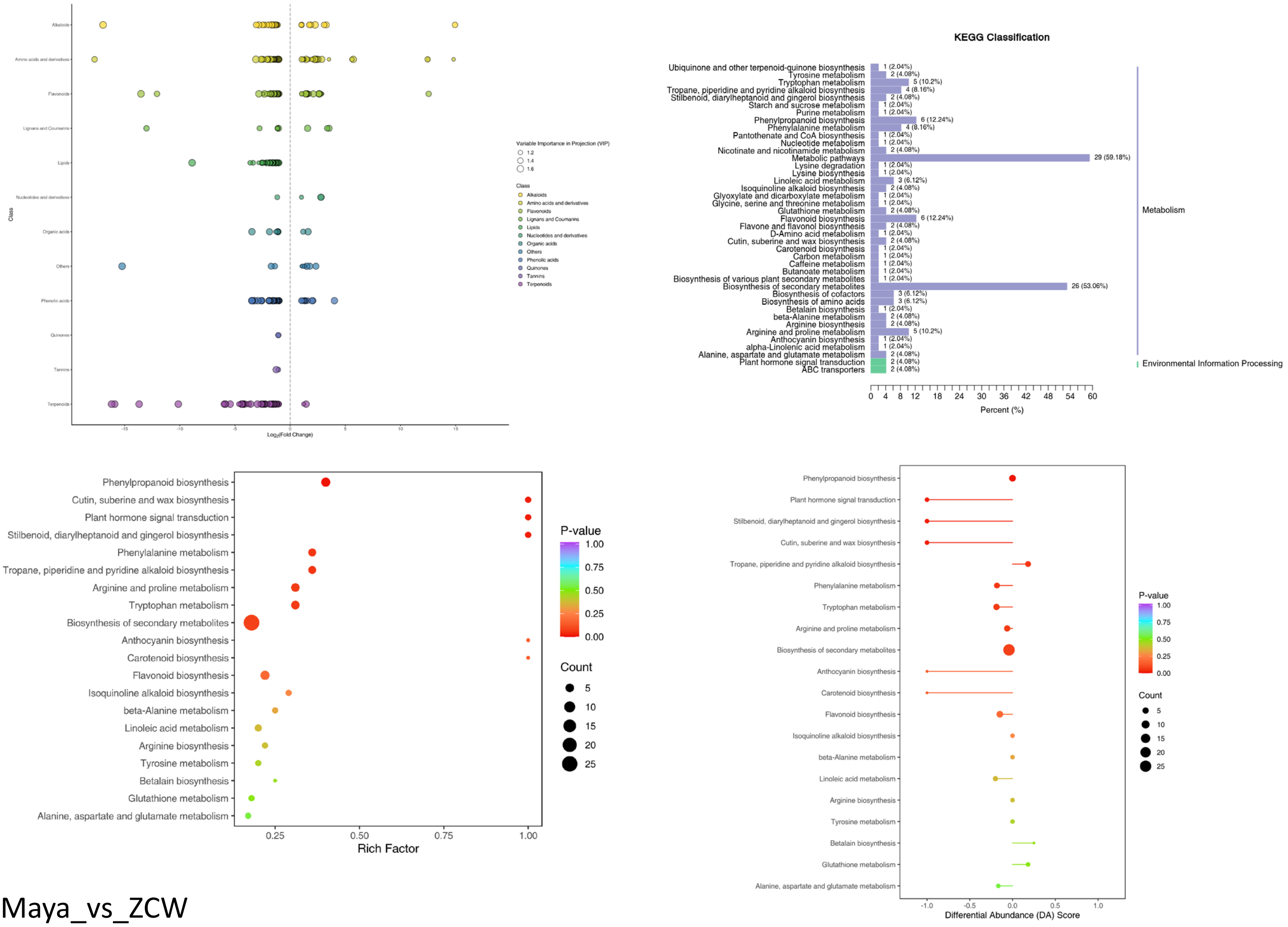


Maya_vs_ZCW

**Figure S5 | Pairwise differential metabolite analysis between *Ziziphus jujuba* cultivars. Scatter plot of differential metabolites shown on top, left**. Each dot in the graph indicates a metabolite, and different colors indicate different metabolite subclasses; the horizontal coordinate indicates the logarithmic value of the multiplicative difference in the content of a substance in two groups of samples (log22FC), the larger the absolute value of the horizontal coordinate, the greater the difference in the content of the substance between the two groups of samples, and the size of the dot represents the VIP value. **KEGG classification of differential metabolites on the top right**. The Y-axis shows the name of the KEGG pathway. The number of significant differential metabolites and the proportion of the total significant differential metabolites are shown next to the bar plot. **KEGG enrichment diagram of differential metabolites on bottom left.** The X-axis represents the Rich Factor and the Y-axis represents the pathway. The color of points reflects the p-value. The darker the red, the more significant the enrichment. The size of the dot represents the number of enriched differential metabolites. **Analysis of overall changes in KEGG metabolic pathway on bottom right.** The Y-axis represents the name of differential pathway, and the X-axis represents DA Score. DA Score reflects the overall change of all metabolites in the metabolic pathway. A Score of 1 indicates that the expression trend of all identified metabolites in this pathway is up-regulated, and -1 indicates that the expression trend of all identified metabolites in this pathway is down-regulated. The length of the line represent the absolute value of DA-score while the size of the dot at the end of the line represent the number of differential metabolites. A dot on the left of the line represent the pathway is down-regulated; a dot on the right of the line represents the pathway is up-regulated. The color of the line and dot represent the p-value. The darker the red, the smaller the p-value and the darker the purple, the larger the p-value.

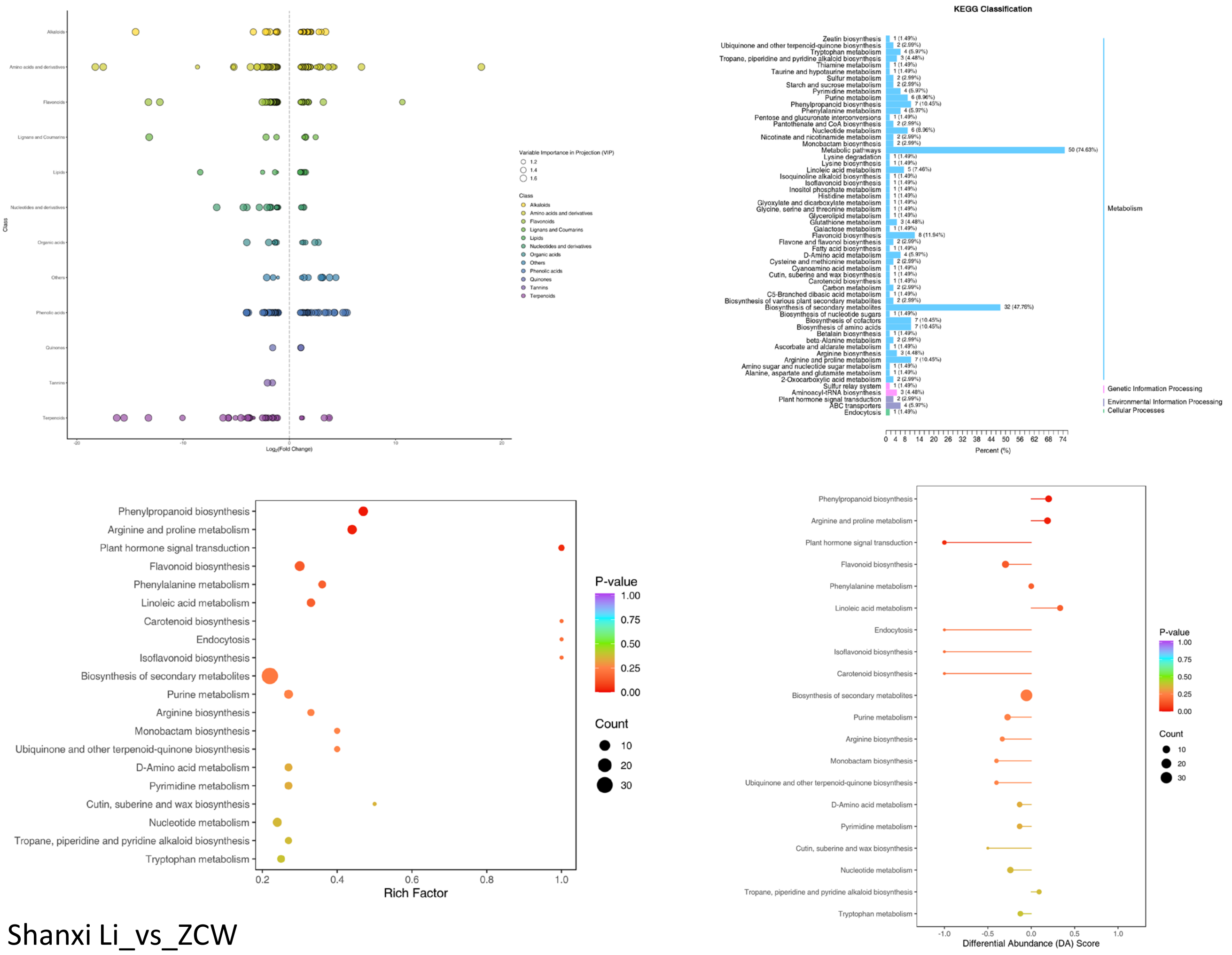


**Figure S5 | Pairwise differential metabolite analysis between *Ziziphus jujuba* cultivars. Scatter plot of differential metabolites shown on top, left**. Each dot in the graph indicates a metabolite, and different colors indicate different metabolite subclasses; the horizontal coordinate indicates the logarithmic value of the multiplicative difference in the content of a substance in two groups of samples (log22FC), the larger the absolute value of the horizontal coordinate, the greater the difference in the content of the substance between the two groups of samples, and the size of the dot represents the VIP value. **KEGG classification of differential metabolites on the top right**. The Y-axis shows the name of the KEGG pathway. The number of significant differential metabolites and the proportion of the total significant differential metabolites are shown next to the bar plot. **KEGG enrichment diagram of differential metabolites on bottom left.** The X-axis represents the Rich Factor and the Y-axis represents the pathway. The color of points reflects the p-value. The darker the red, the more significant the enrichment. The size of the dot represents the number of enriched differential metabolites. **Analysis of overall changes in KEGG metabolic pathway on bottom right.** The Y-axis represents the name of differential pathway, and the X-axis represents DA Score. DA Score reflects the overall change of all metabolites in the metabolic pathway. A Score of 1 indicates that the expression trend of all identified metabolites in this pathway is up-regulated, and -1 indicates that the expression trend of all identified metabolites in this pathway is down-regulated. The length of the line represent the absolute value of DA-score while the size of the dot at the end of the line represent the number of differential metabolites. A dot on the left of the line represent the pathway is down-regulated; a dot on the right of the line represents the pathway is up-regulated. The color of the line and dot represent the p-value. The darker the red, the smaller the p-value and the darker the purple, the larger the p-value.

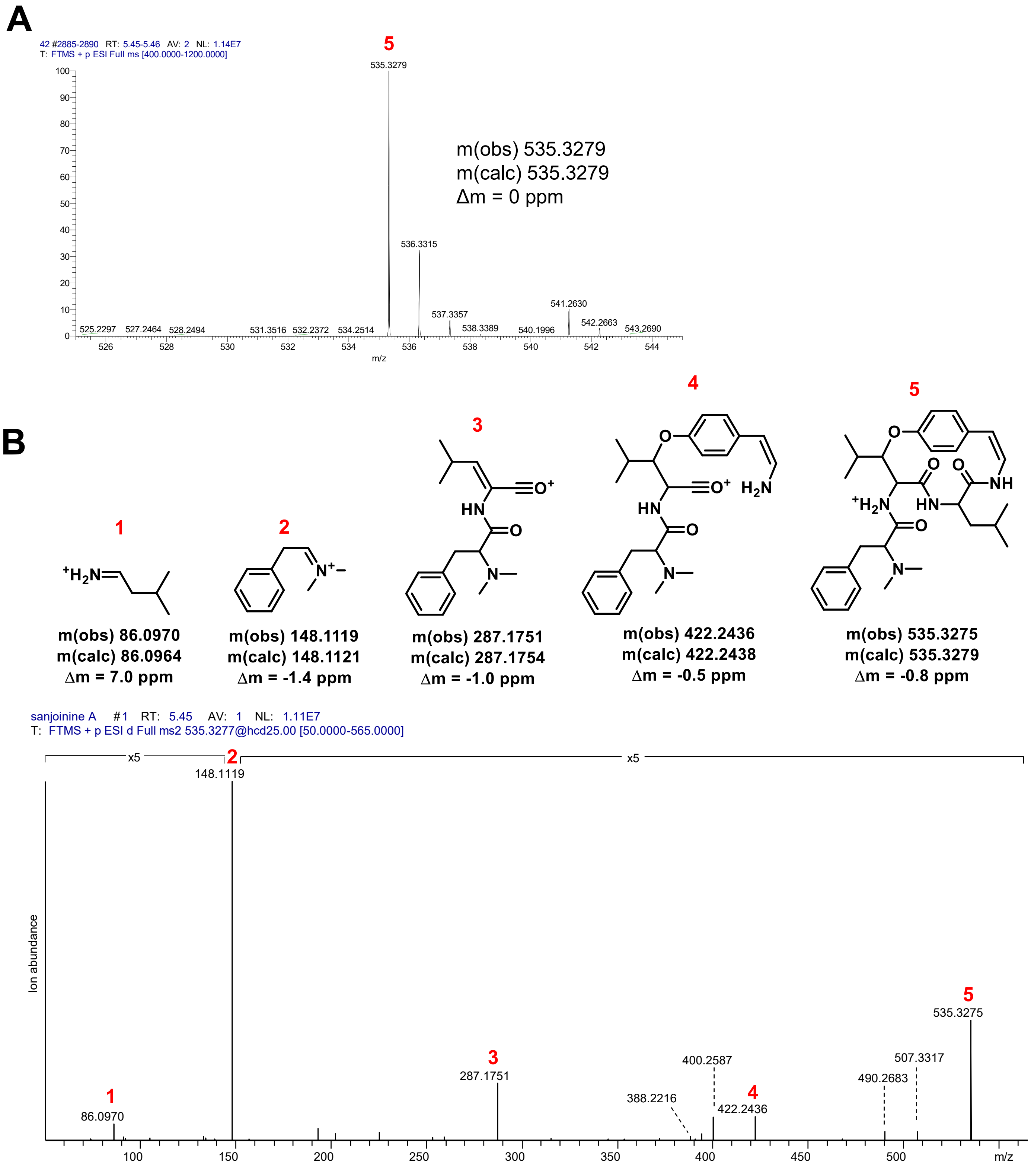


**Figure S6 | MS analysis of a sanjoinine A.** (**A**) MS1 analysis of sanjoinine A analyte and (**B**) MS/MS analysis of sanjoinine A analyte.

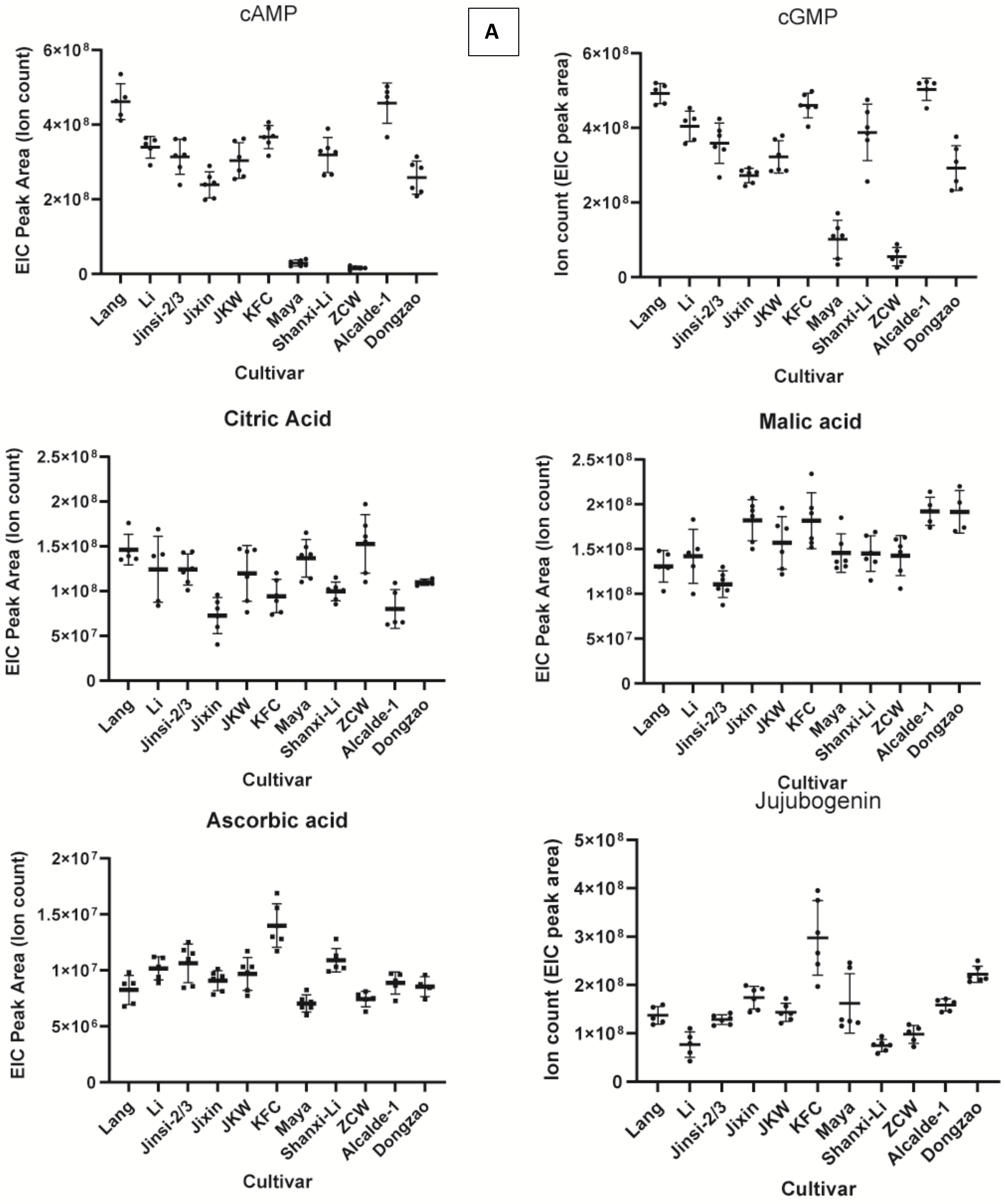
A
cAMP
EIC Peak Area (Ion count)
6×10⁸
4×10⁸
2×10⁸
0
Lang
Li
Jinsi-2/3
Jixin
JKW
KFC
Maya
Shanxi-Li
ZCW
Alcalde-1
Dongzao
Cultivar
cGMP
Ion count (EIC peak area)
6×10⁸
4×10⁸
2×10⁸
0
Lang
Li
Jinsi-2/3
Jixin
JKW
KFC
Maya
Shanxi-Li
ZCW
Alcalde-1
Dongzao
Cultivar
Citric Acid
EIC Peak Area (Ion count)
2.5×10⁸
2×10⁸
1.5×10⁸
1×10⁸
5×10⁷
0
Lang
Li
Jinsi-2/3
Jixin
JKW
KFC
Maya
Shanxi-Li
ZCW
Alcalde-1
Dongzao
Cultivar
Malic acid
EIC Peak Area (Ion count)
2.5×10⁸
2×10⁸
1.5×10⁸
1×10⁸
5×10⁷
0
Lang
Li
Jinsi-2/3
Jixin
JKW
KFC
Maya
Shanxi-Li
ZCW
Alcalde-1
Dongzao
Cultivar
Ascorbic acid
EIC Peak Area (Ion count)
2×10⁷
1.5×10⁷
1×10⁷
5×10⁶
0
Lang
Li
Jinsi-2/3
Jixin
JKW
KFC
Maya
Shanxi-Li
ZCW
Alcalde-1
Dongzao
Cultivar
Jujubogenin
Ion count (EIC peak area)
5×10⁸
4×10⁸
3×10⁸
2×10⁸
1×10⁸
0
Lang
Li
Jinsi-2/3
Jixin
JKW
KFC
Maya
Shanxi-Li
ZCW
Alcalde-1
Dongzao
Cultivar

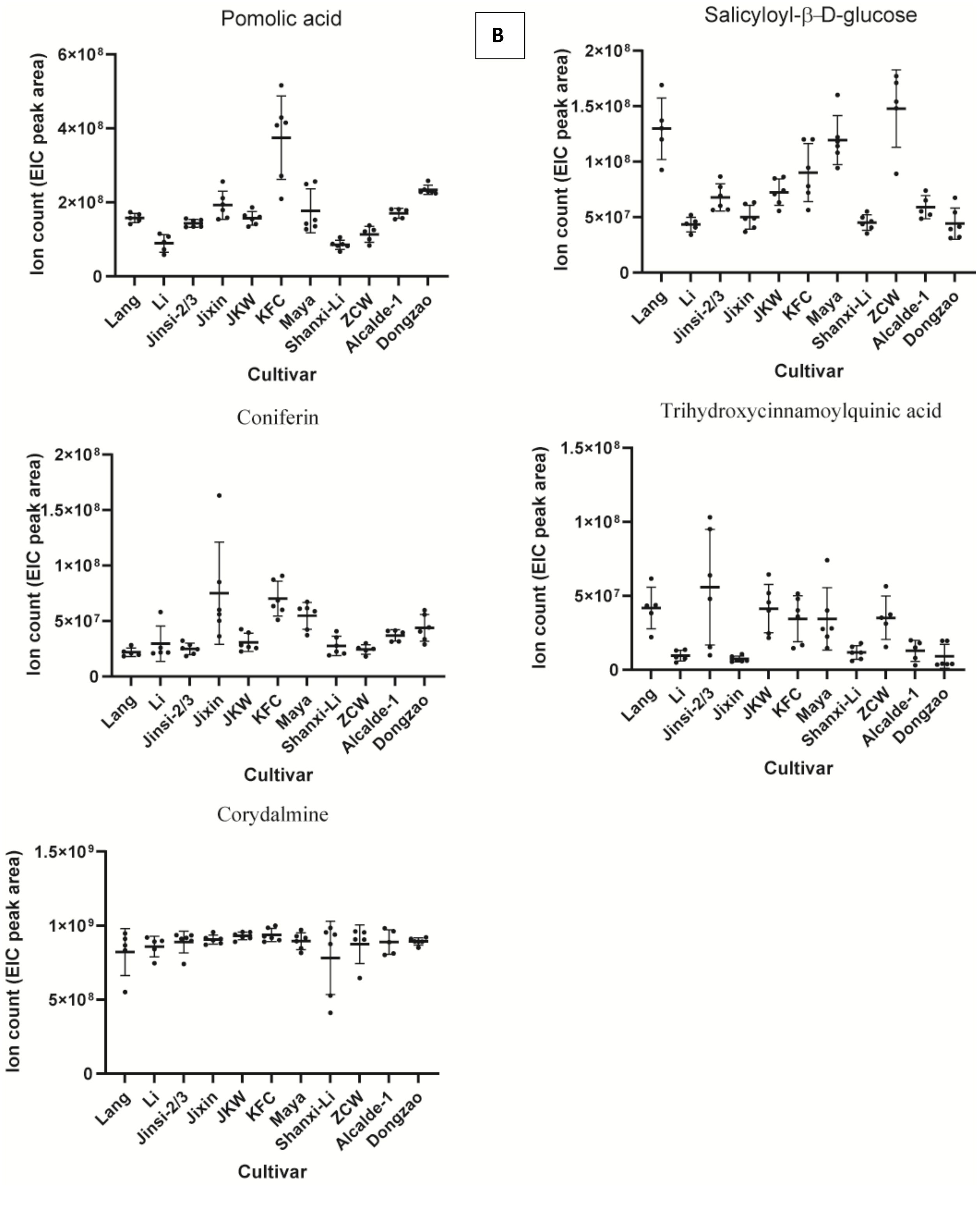


**Figure S7.** Significant metabolites of 11 jujube cultivars in different categories. A: cAMP, cGMP, citric acid, malic acid, ascorbic acid and jujubogenin, B: pomolic acid, salycolyl-β-D-glucose, coniferin, trihydroxycinnamoylquinic acid, and corydalmine.The horizontal lines in cultivar plots represent the mean (n=5-6) and the error bars represent one standard deviation.
